# Network Modeling Identifies Molecular Functions Targeted by miR-204 to Suppress Head and Neck Tumor Metastasis

**DOI:** 10.1371/journal.pcbi.1000730

**Published:** 2010-04-01

**Authors:** Younghee Lee, Xinan Yang, Yong Huang, Hanli Fan, Qingbei Zhang, Youngfei Wu, Jianrong Li, Rifat Hasina, Chao Cheng, Mark W. Lingen, Mark B. Gerstein, Ralph R. Weichselbaum, H. Rosie Xing, Yves A. Lussier

**Affiliations:** 1 Section of Genetic Medicine and Center for Biomedical Informatics, Department of Medicine, The University of Chicago, Chicago, Illinois, United States of America; 2 Department of Pathology, The University of Chicago, Chicago, Illinois, United States of America; 3 Department of Genetics, Yale University School of Medicine, New Haven, Connecticut, United States of America; 4 Department of Cellular and Radiation Oncology, The University of Chicago, Chicago, Illinois, United States of America; 5 Ludwig Center for Metastasis Research, The University of Chicago, Chicago, Illinois, United States of America; 6 Institute for Genomics and Systems Biology, The University of Chicago, Chicago, Illinois, United States of America; Washington University in Saint Louis, United States of America

## Abstract

Due to the large number of putative microRNA gene targets predicted by sequence-alignment databases and the relative low accuracy of such predictions which are conducted independently of biological context by design, systematic experimental identification and validation of every functional microRNA target is currently challenging. Consequently, biological studies have yet to identify, on a genome scale, key regulatory networks perturbed by altered microRNA functions in the context of cancer. In this report, we demonstrate for the first time how phenotypic knowledge of inheritable cancer traits and of risk factor loci can be utilized jointly with gene expression analysis to efficiently prioritize deregulated microRNAs for biological characterization. Using this approach we characterize miR-204 as a tumor suppressor microRNA and uncover previously unknown connections between microRNA regulation, network topology, and expression dynamics. Specifically, we validate 18 gene targets of miR-204 that show elevated mRNA expression and are enriched in biological processes associated with tumor progression in squamous cell carcinoma of the head and neck (HNSCC). We further demonstrate the enrichment of bottleneckness, a key molecular network topology, among miR-204 gene targets. Restoration of miR-204 function in HNSCC cell lines inhibits the expression of its functionally related gene targets, leads to the reduced adhesion, migration and invasion in vitro and attenuates experimental lung metastasis in vivo. As importantly, our investigation also provides experimental evidence linking the function of microRNAs that are located in the cancer-associated genomic regions (CAGRs) to the observed predisposition to human cancers. Specifically, we show miR-204 may serve as a tumor suppressor gene at the 9q21.1–22.3 CAGR locus, a well established risk factor locus in head and neck cancers for which tumor suppressor genes have not been identified. This new strategy that integrates expression profiling, genetics and novel computational biology approaches provides for improved efficiency in characterization and modeling of microRNA functions in cancer as compared to the state of art and is applicable to the investigation of microRNA functions in other biological processes and diseases.

## Author Summary

MicroRNAs regulate the expression of genes in cells and are important in cancer development and progression. Designing new microRNA-based treatments requires the understanding of their mechanisms of action. Previous biological studies lack in depth since only a few genes are confirmed as microRNA targets. Additionally, key biological systems perturbed by altered microRNA functions in the context of cancer remain to be identified. Here, we demonstrate for the first time how genetic knowledge about the inheritance of cancer can be utilized jointly with data about the expression of genes in cancer samples to model deregulated microRNAs and their functions at multiple scales of biology. Our approach further uncovers previously unknown connections between microRNAs, their regulated genes, and their dynamics. Using head and neck cancer as a model, we predict the presence, functions, and gene targets of a new tumor suppressor microRNA in a cancer-associated chromosomal region where a candidate gene has not been identified. We then confirm their validity with extensive and thorough biological characterization and show attenuation of lung metastasis in mice. The discovery of molecular networks regulated by microRNAs could be exploited for the design of new treatments as an alternative to the single-gene target paradigm.

## Introduction

Since the discovery of microRNAs as important regulators of broad biological processes [1]–[5], characterization of their functions in cancer has been hindered by lack of microRNA profiling information in tumors such as squamous cell carcinoma of the head and neck (HNSCC). Previous reports show that only one or a few gene targets, identified among predicted or differentially expressed genes, were directly targeted by the microRNA under investigation [6]–[8]. While sequence-based computational algorithms have been applied for predicting all potential microRNA gene targets; false positive rates remains relatively high [9],[10]. Further, sequence-based predictions are unable, by design, to account for biological contexts (e.g. cell and tissue types, normal or disease conditions) and thus are not optimized for predicting the biological function of genes targeted by cancer microRNAs. Moreover, genome-scale and biological studies have yet to identify key regulatory networks perturbed by altered microRNA functions in cancer.

To investigate microRNA function in HNSCC, we sought to develop an effective computational approach that is complementary to microRNA profiling and, in addition, is capable of simultaneously predicting tumor suppressor microRNAs as well as their functional targets from gene expression. In this report we illustrate how phenotypic knowledge of genetic disorders (OMIM database) can be utilized jointly with gene expression analyses to achieve this goal. Using this approach, we selected miR-204 among ten prioritized microRNAs for biological characterization, as miR-204 is located at the cancer-associated genomic region (CAGR) 9q21.1–q22.3 locus exhibiting high frequency of Loss of heterozygosity (LOH) in human HNSCC [11]–[15], and a CAGR for which candidate tumor suppressor gene targets have not been identified. Additionally, we report the first computationally predicted and biologically validated microRNA-regulated network that is dependent on the epidermal growth factor receptor (EGFR) whose overexpression occurs in over 80% of head and neck cancer. We further demonstrate that gene targets of miR-204 exhibit enriched bottleneck and hub network topology properties in a predicted protein-protein interaction network (PPIN). Moreover, we confirm the validity of our computational predictions of a microRNA function, as well as its gene targets and system's properties through conducting extensive and thorough biological characterization using a clinically relevant *in vivo* metastatic model of head and neck cancer.

In summary, we show how such a high throughput system's strategy can accelerate the investigation of microRNA function in cancer by illustrating altered complex biological processes and regulatory pathways associated with microRNA dysfunction in cancer, by identifying among all putative microRNA gene targets only those that are dysregulated, and by elucidating molecular interactions underpinning microRNA regulation of malignant transformation and progression. The ability to characterize tumor suppressor microRNAs through a network analysis of mRNA expression datasets would be a major advance with potentially wide application. Further, we provide experimental evidence linking microRNA function to the genetic risk of HNSCC. We show at the LOH 9q21.1–22.3 locus, miR-204 could serve as a tumor suppressor of HNSCC oncogenesis and progression.

## Results

A figure summarizing the main results and experimental approaches of this paper is included as Supporting Figure 1 in Text S1.

### Combining genome-scale predictive strategies to prioritize candidate microRNAs for biological characterization in HNSCC

At the time of initiating this study, comprehensive analysis of microRNA expression profile in head and neck cancer (HNSCC) was not available and would have required time-consuming accruement of tumor tissues for conducting such analysis, a situation that is not limited to HNSCC research. We hypothesized that the development of a computational capability to simultaneously predict tumor suppressor microRNAs as well as their functional targets from more widely available genome-wide gene expression datasets could be an efficient reverse engineering approach for identifying deregulated microRNAs and their functional gene targets.

We first developed IMRE, a statistical method to predict altered expression of microRNAs from genome-wide mRNA expression and putative microRNA targets databases (Supporting Figure 2 in Text S1, Materials and Methods). This strategy is based, in part, on the observations that at genome scale the expression of microRNAs and their direct mRNA targets are, in general, inversely correlated [16],[17]. To conduct this analysis, we integrated five complementary microRNA target databases to generate “miRNOME” that contains 534 human microRNAs and 17,343 microRNA gene targets (Materials and Methods, and Table 1 in Text S2). We validated this method using two independent cancer expression profiling experiments in GEO comprised of paired mRNA and microRNA expressions for tumors and normal tissue (GSE2564 [18]: multiple epithelial cancer; GSE8126 [19]: prostate cancer). IMRE-predicted downregulated microRNAs that are exclusively inferred from mRNA expression and microRNA targets datasets (Materials and Methods) were enriched in the expression analysis of the corresponding microRNA array dataset (GSE2564: *P* = 0.014; GSE8126: *P* = 0.0002 respectively, cumulative hypergeometric test, data not shown). A recent study also demonstrated the increased prediction specificity of microRNA and its gene target relationship via intersecting the results of multiple prediction algorithms [20].

**Figure 1:**
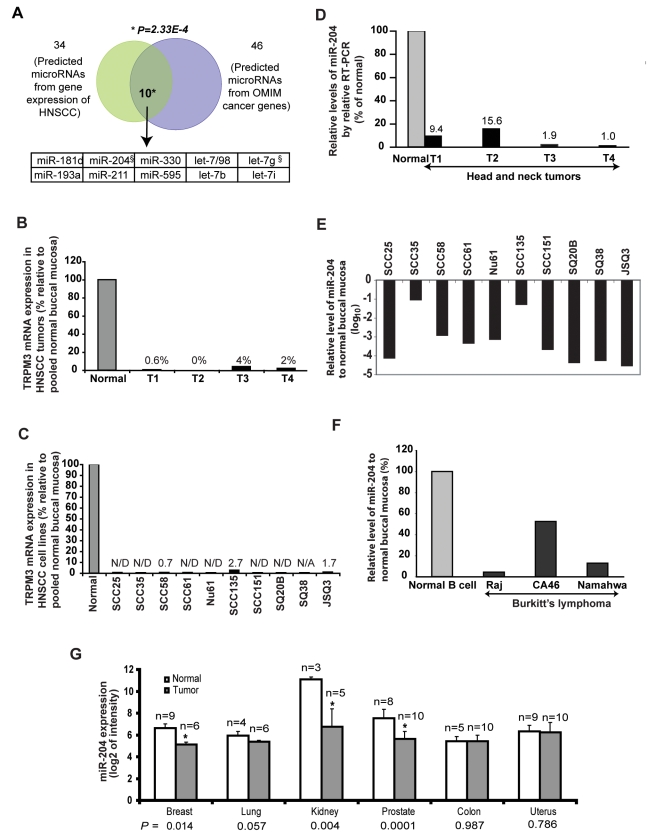
Combining genome-scale predictive strategies to predict and prioritize candidate microRNAs in HNSCC. (**A**) Enriched gene targets of 46 microRNAs among inheritable cancer genes in OMIM are significantly overlapping with 34 predictions of deregulated microRNAs based on HNSCC expression arrays (GSE6631, GSE2379; Figure S3; Table S2 and Table S3), yielding ten prioritized microRNAs (*P* = 2.33×10^−4^). *P*: Cumulative hypergeometric Statistics. §: miR-204 and let-7g are located in chromosomal regions with known increased genetic risk of HNSCC (9q21.1–22.1 for miR-204 and 3p21 for let-7g, respectively) [15]. (**B–C**) mRNA expression of TRPM3, the host gene of miR-204, is significantly downregulated and is barely detectable in four microdissected head and neck tumors (B) and in a panel of ten low passage HNSCC cell lines [26] (C). TRPM3 mRNA expression was determined by qRT-PCR and normalized with the TBP endogenous gene control. Triplicate real time PCR measurements were obtained and the mean Ct (cycle threshold) was used to calculate RQ values. Standard deviation of the triplicate measurement was less than 0.15 Ct. Shown are relative tumor TRPM3 mRNA expression levels compared with five pooled normal buccal mucosa. (**D–F**) Quantification of miR-204 expression by Taqman qPCR in four laser micro-captured head and neck tumors (**D**), in ten HNSCC cell lines (**E**) and in three Burkitt's B-cell lymphoma cell lines (**F**). Five equally pooled normal buccal mucosa RNAs were used as the normal control for HNSCC tumors and cell lines. Normal B cell RNA was used as the normal control for Burkitt's B-cell lymphoma. (**G**) Comparison of miR-204 expression between six types of human epithelial cancers tissues and their respective normal tissues was conducted using the microRNA profiling dataset GSE2564 [18] (*P-values* were calculated using two-tail unpaired t-test; “n” indicates number of patients; error bars represent mean ± standard error of the mean, Materials and Methods).

Subsequently, we applied the IMRE method to analyze two independent HNSCC mRNA microarray datasets for predicting deregulated microRNAs from genome-wide mRNA expression (Supporting Figure 2 in Text S1, Materials and Methods): first, the GSE6631 set that provides differential mRNA gene expression between 22 HNSCC non-microdissected patient tumor samples and their paired normal squamous tissues [21], and second, the GSE2379 [22] set that contains 34 micro-dissected node-positive HNSCC tumors of the hypopharynx. We noted that vast majority of the known microRNAs had at least one putative target in the top 500 deregulated genes of the HNSCC expression arrays (GSE6631), with a median of 19 targets. Therefore, it is unfeasible to manually select microRNA candidates from their deregulated targets for biological validation. Applying IMRE method to each dataset, we predicted a set of down-regulated microRNAs in HNSCC (113 and 43, respectively; FDR ≤0.05, Materials and Methods), of which 34 were consistently found in both prediction sets (*P-value* = 2.0×10^−16^, Fisher's exact test, Figure 1A and Table 2 in Text S2, FDR <0.05, Materials and Methods). Predictions of up-regulated microRNAs did not reach reproducible statistical significance (not shown).

To further reduce the number of microRNAs to the most promising candidates for HNSCC, we conducted a statistical enrichment analysis of putative microRNA targets among inheritable cancer genes in the OMIM human disease gene database [Online Mendelian Inheritance in Man, http://www.ncbi.nlm.nih.gov/omim/ (downloaded Dec. 1, 2006)]. OMIM contains 610 biologically validated cancer genes among which 586 (96%) are predicted targets of 527 microRNAs in miRNOME. We observed that each of the 527 microRNAs could, on average, target 30 OMIM cancer genes (not shown). Thus, it is also unfeasible to manually select microRNA candidates from OMIM cancer genes for biological validation. Our analyses identified 46 microRNAs significantly enriched in the inheritable cancer gene subset of OMIM in the miRNOME (Figure 1A; Table 3 in Text S2, Materials and Methods, Protocol S1/Section A, and Dataset S1). Since microRNAs can be deregulated across cancers of different tissue origin [23], we performed a review of literature and confirmed the validity of these 46 predictions (OMIM; Supporting Figure 3 in Text S1, *P* = 0.039; cumulative hypergeometric test, Table 4 in Text S2, Materials and Methods).

Thereafter, we reduced the list of candidates in HNSCC to ten microRNAs (Figure 1A) that were predicted in the HNSCC gene expression (34 microRNAs; Table 2 in Text S2) as well as in inheritable cancer genes (46 microRNAs; Table 3 in Text S2). Among the ten prioritized microRNAs, four belong to the let-7 tumor suppressor microRNA family (Figure 1A).

### miR-204 is located at the genomic imbalanced 9q21.1-22.3 locus associated with genetic predisposition for head and neck cancer

We chose miR-204 among the ten prioritized microRNAs for thorough biological characterization based on the following considerations. First, miR-204 is located within the sixth intron of the host gene transient receptor potential melastatin 3 cation channel (TRPM3, NM_020952) and is transcribed in the same direction as TRPM3 [24]. TRPM3 is located on human chromosome 9q21.11 that is within the 9q21.1–q22.3 locus exhibiting high frequency of Loss of heterozygosity (LOH) in human HNSCC [11]–[15]. LOH at 9q21.1–q22.3 occurs in 37% of premalignant head and neck lesions, and increases to 67% in HNSCC [14]. Second, in addition to the genomic imbalance at 9q21.1–q22.3 locus, chromosomal aberrations occur most frequently at 3p, 5q, 9p, 11q and 17p in HNSCC [11],[12],[25]. With the exception of let-7g that is located at the 3p21 locus (note that let7g is also included in the class of microRNAs with related mature sequence “Let7/98”), the other 7 prioritized microRNAs are not in the cancer associated genomic regions (CAGRs). Third, while potential tumor suppressor gene candidates have been identified for other CAGRs in HNSCC, gene candidates possessing tumor suppressor activity associated with the 9q21 locus have not been identified. Thus the mechanisms by which changes at this locus affecting HNSCC oncogenesis remain uncharacterized. Fourth, the role of miR-204 in human cancer has not been established.

We first examined miR-204 host gene TRPM3 expression by quantitative PCR (qPCR) and observed near complete TRPM3 suppression in four micro-dissected HNSCC tumors (Figure 1B) and in a panel of 10 low passage HNSCC cell lines generated from tumors of diverse head and neck locations (Figure 1C and Table 5 in Text S2) [26]. We subsequently measured miR-204 expression in HNSCC tumors and cell lines. Consistent with the observed near complete loss of TRPM3 (Figures 1B–C), miR-204 expression was inhibited in all four tumors by 85% to 99% (Figure 1D), and by more than 90% in all ten HNSCC cell lines (Figure 1E) compared to samples of pooled normal buccal mucosa. The frequent allelic loss at 9q21.1–q22.3 in HNSCC [11]–[14] provides genetic evidence that loss of miR-204 microRNA function may occur as a result of genomic imbalance at this site and that miR-204 may be a potential candidate associated with the tumor suppressor activity of 9q21.1–q22.3.

Since miR-204 was also predicted in the OMIM analysis to be associated with lymphoma (Table 3 in Text S2), we quantified miR-204 expression in immortalized “normal B cell 11365” and three Burkitt B-cell lymphoma cell lines and found its expression significantly reduced (Figure 1F). Further, paired comparison of miR-204 expression between 6 types of adenocarcinomas and their respective normal tissues was conducted using the microRNA array dataset GSE2564 [18]. miR-204 was significantly down-regulated in breast (*P* = 0.014), kidney (*P* = 0.004) and prostate (*P* = 0.0001) tumors (Figure 1G). Additionally, significant miR-204 down-regulation was recently reported in a subtype of acute myeloid leukemia bearing cytoplasmic mutated nucleophosmin [27]. Here, we demonstrate for the first time, the accuracy and efficiency of joint analyses of mRNA expression, inheritable disease genes, and microRNA target databases to prioritize deregulated microRNAs for biological characterization. Collectively, these biological findings support the validity of our computational predictions of miR-204 downregulation in HNSCC and suggest that it may possess tumor suppressor activity.

### Predicted miR204 gene targets are significantly related through their biological functions

**Figure 2:**
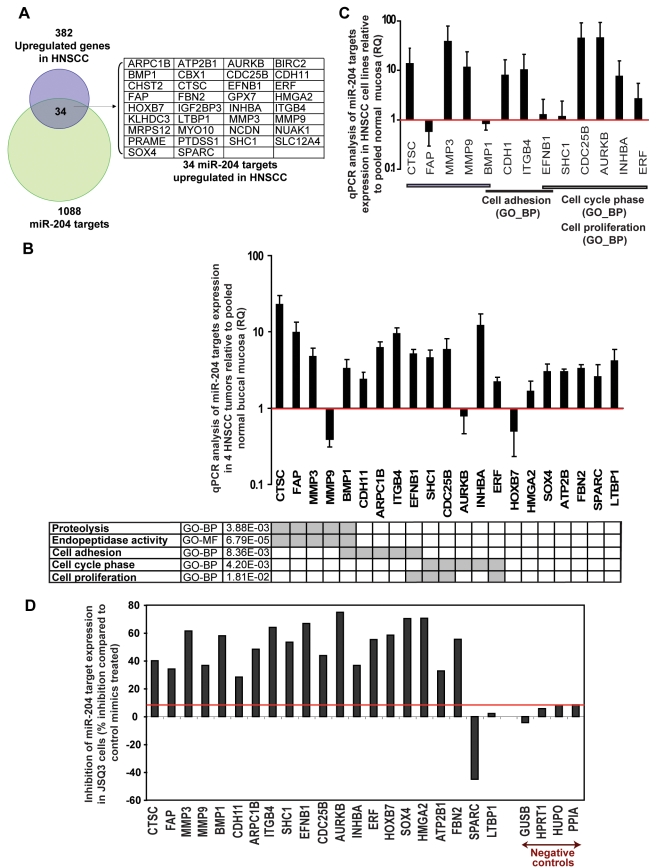
Predicted targets of miR-204 in HNSCC are significantly related via their molecular or biological functions. (**A**) Enrichment of 34 miR-204 gene targets between 382 differentially upregulated HNSCC genes (GSE6631) and 1088 putative miR-204 targets predicted by sequence-based microRNA target prediction databases (miRNOME); (**B–C**) Determination of mRNA expression of “functionally prioritized miR-204 targets” in four laser-microdissected HNSCC tumor samples (**B**) and in 10 HNSCC cell lines (**C**) by qPCR as described above in Figure 1 and in Materials and Methods). Five equally pooled normal buccal mucosa RNAs were used as the normal control. Legend: red line (RQ = 1) is the expression of the TBP endogenous gene control; error bars represent standard error of the mean; shaded squares in the GO table indicate gene targets in the corresponding “biological processes” (BP) and “molecular functions” (MF) of Gene Ontology (GO); adjusted *P-values* indicate the combined statistical enrichment of these genes in GO (Materials and Methods). (**D**) Ectopic enhancement of miR-204 function inhibited its predicted gene targets mRNA expression in JSQ3 HNSCC cell line. TBP expression was used as an endogenous gene control for normalization (red line) in the qPCR analysis.

Among the 1,088 putative miR-204 targets predicted in the miRNOME, 34 mRNA transcripts that were significantly upregulated in HNSCC (GSE6631) led to the enrichment of miR-204 (Figure 2A and Table 6 in Text S2). We first conducted statistical functional enrichment analyses using Gene Ontology (GO) [28] and found a number of biological processes (BP) and molecular functions (MF) of GO were significantly enriched among 32 of the 34 miR-204 gene targets (referred to as “functionally prioritized miR-204 targets”) (Table 7 in Text S2, Materials and Methods, and Protocol S1/Section C). We next examined mRNA expression status of 21 representative “functionally prioritized miR-204 targets” in four laser capture microdissected HNSCC tumor samples and observed increased expression of 18 of these genes compared with their respective expression in five pooled normal buccal mucosa (Figure 2B). Additionally analysis of thirteen “functionally prioritized miR-204 targets”, those enriched with the listed GO functions (the table in Figure 2B, Materials and Methods), showed overexpression of nine targets in ten HNSCC cell lines (Figure 2C). These results indicate that predicted miR-204 targets, upregulated in HNSCC, share similar functions and may participate in similar biological processes.

### miR-204 suppresses the expression of its functionally prioritized targets

To provide evidence that miR-204 can directly suppress the expression of its predicted targets in HNSCC, examination of the 3′UTR confirmed that all 34 predicted target genes contain at least one miR-204 binding site as expected by our predictions using sequence homology databases of the miRNOME (Table 8 in Text S2, and Materials and Methods). Thereafter, we selected 21 “functionally prioritized miR-204 targets” overexpressed in HNSCC (Figure 2B) for biological validation. We conducted *in vitro* miR-204 gain-of-function analyses by transiently transfecting JSQ3 and SQ38 HNSCC cells with mature miR-204 mimics (Dharmacon) to enhance miR-204 function in these two cell lines. Restoration of miR-204 function achieved significant inhibition (between 30% to 75%) of endogenous mRNA expression in 18 out of 21 predicted targets examined for both cell lines, while non-specific control mimics had no significant effect (Figure 2D and Supporting Figures 4–5 in Text S1). The specificity of miR-204 mimics was further confirmed by unaltered expression of four endogenous housekeeping genes (GUSB, HPRT1, HUPO and PPIA) that lack target homology to miR-204 (Figure 2D and Supporting Figure 4 in Text S1). Comparing with sequence-based microRNA gene target prediction algorithms that have true positive rates of about 40% [9],[10], the accuracy of our prediction methods is higher (∼90%). Collectively, these observations indicate that down-regulation of functionally related miR-204 targets upon miR-204 mimics treatment was sequence specific and was not due to artifacts of transfection or the “off target” effect of miR-204 mimics.

### miR-204 gene targets exhibit significant topological properties in a HNSCC protein interaction network predicted by network modeling

**Figure 3:**
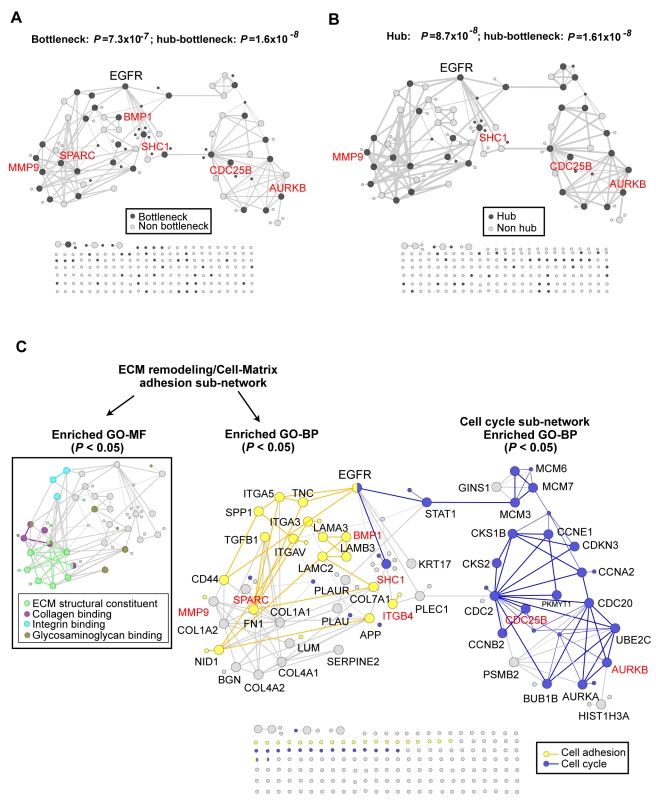
miR-204 gene targets exhibit significant topological properties in a predicted protein interaction network of HNSCC based on single protein network modeling. (**A–B**) A 56-gene “prioritized HNSCC PPIN” was predicted from single protein network modeling and was significantly enriched with bottleneck (*P* = 7.3×10^−7^), hub (*P* = 8.7×10^−8^) and hub-bottleneck genes (*P* = 1.6×10^−8^). *P-values* were calculated using one-tailed cumulative hypergeometric tests. Genes colored in red: miR-204 gene targets. (**C**) Gene Ontology enrichment analysis of the “biological processes” (BP) and “molecular functions” (MF) identified two EGFR-dependent sub-networks in the “prioritized HNSCC PPIN” (adjusted *P*<0.05). Different BPs and MFs were coded by colors as indicated. Every gene analyzed in the network are represented as circles, the majority do not reach statistical significance and remain as unnamed grey dots on the bottom of the figure (statistical details and names are provided in Table S11, and their interactions in Table S12).

Following functional enrichment analysis of upregulated miR-204 targets in HNSCC, we next examined the role of miR-204 targets in modulating the function of a protein-protein interaction network (PPIN). To identify genome-wide changes in PPINs associated with altered microRNA functions in HNSCC, we first integrated seven protein-protein interaction databases (Materials and Methods) and generated a “genome-scale PPIN” that contains 44,695 protein-protein interactions and 7,321 predicted human genes targets for the 532 microRNAs in the miRNOME. We subsequently could map 260 out of 382 (68%) up-regulated genes in GSE6631 to the PPIN (refer to as “HNSCC PPIN”), of which 24 were miR-204 targets predicted in miRNOME. We next computed the empirical probability of interactions among these 260 genes in the network using permutation resampling (Materials and Methods). To identify the most important interactions in the HNSCC PPIN, we retained proteins for which the number of observed interactions was significantly increased in single protein network modeling as compared to those found in the empirical distribution (Materials and Methods). As a result, we identified a protein regulatory network in HNSCC consisting of 56 prioritized upregulated genes in GSE6631 at a low false discovery rate of 7% (Figure 3 and, Materials and Methods) (referred to as “prioritized HNSCC PPIN”). Among the 24 miR-204 targets mapped to the genome-scale PPIN, seven were present in the “prioritized HNSCC PPIN” (Figure 3, shown in red). Further, six of the seven-miR-204 targets remained prioritized when computed using different network modeling conditions demonstrating the robustness of our analyses (not shown, and Materials and Methods).

We next analyzed two topological features of the PPIN: the “hub” and “bottleneck” properties. “Hubs”, the highly connected node proteins, and “bottlenecks”, the key connector proteins, are central to controlling the connectivity of biological sub-networks to one another [29]. Further, our prior studies showed proteins possessing both properties (hub-bottleneck) as essential and efficient network components to alter the functional output of a PPIN upon their dynamic changes in gene expression [30]. Here, we observed significant enrichment of hubs, bottlenecks, and hub-bottleneck proteins in the 56-gene “prioritized HNSCC PPIN” as compared to either the “genome-scale PPIN” or to the “HNSCC PPIN” (hub: *P* = 8.7×10^−8^; bottleneck: *P* = 7.31×10^−7^; hub-bottleneck: *P* = 1.61×10^−8^; Materials and Methods). Additionally, the proportion of hub-bottleneck genes was further enriched among the seven miR-204 targets present in the “prioritized HNSCC PPIN” (*P* = 0.002; Fisher's exact test, MMP9, SHC1, CDC25B and AURKB in Figures 3A–B). Moreover, in a genome-scale analysis, we observed a statistically significant association between the proteins that exhibit PPIN network topology, such as hub and bottleneck properties, and the number of predicted microRNA targets. Indeed, bottleneck proteins and hub-bottleneck protein of the “genome-scale PPIN” were both targeted on average by more microRNAs than those that are neither bottleneck nor hub-bottleneck (bottleneck: *P* = 0.0009; hub-bottleneck: *P* = 0.022, Materials and Methods). These results indicate that the enrichment of bottleneck and hub-bottleneck properties among miR-204 gene targets in the “prioritized HNSCC PPIN” is a system's property of microRNAs. They also suggest that the efficiency and specificity of microRNAs in regulating biological functions is further strengthened through alteration of the translation of these bottleneck proteins.

In a protein-protein interaction network, proteins that are tightly linked are likely to function in the same biological process or pathways [31],[32]. To characterize functional relationships among the 56 interacting proteins in the “prioritized HNSCC PPIN”, we conducted statistical enrichment analysis using Gene Ontology (Materials and Methods, Protocol S1/Section C). The biological processes (BP) and molecular functions (MF) enriched in this network (Figure 3C, Materials and Methods) overlapped with our findings of functional enrichment among 34 predicted miR-204 targets (Figures 2B–C). Two EGFR-dependent regulatory sub-networks were identified: cell cycle regulation and extracellular matrix (ECM) remodeling/Cell-matrix adhesion (Figure 3C). Based on the importance of hub-bottleneck genes in regulating the function of a PPIN [33], the enrichment of four hub-bottlenecks miR-204 targets in the EGFR-dependent “prioritized HNSCC PPIN” predicts that their up-regulation upon miR-204 suppression in HNSCC could significantly augment cell cycle and extracellular matrix remodeling.

### miR-204 suppressed HNSCC cell migration, adhesion and invasion *in vitro* and lung colonization *in vivo*

**Figure 4:**
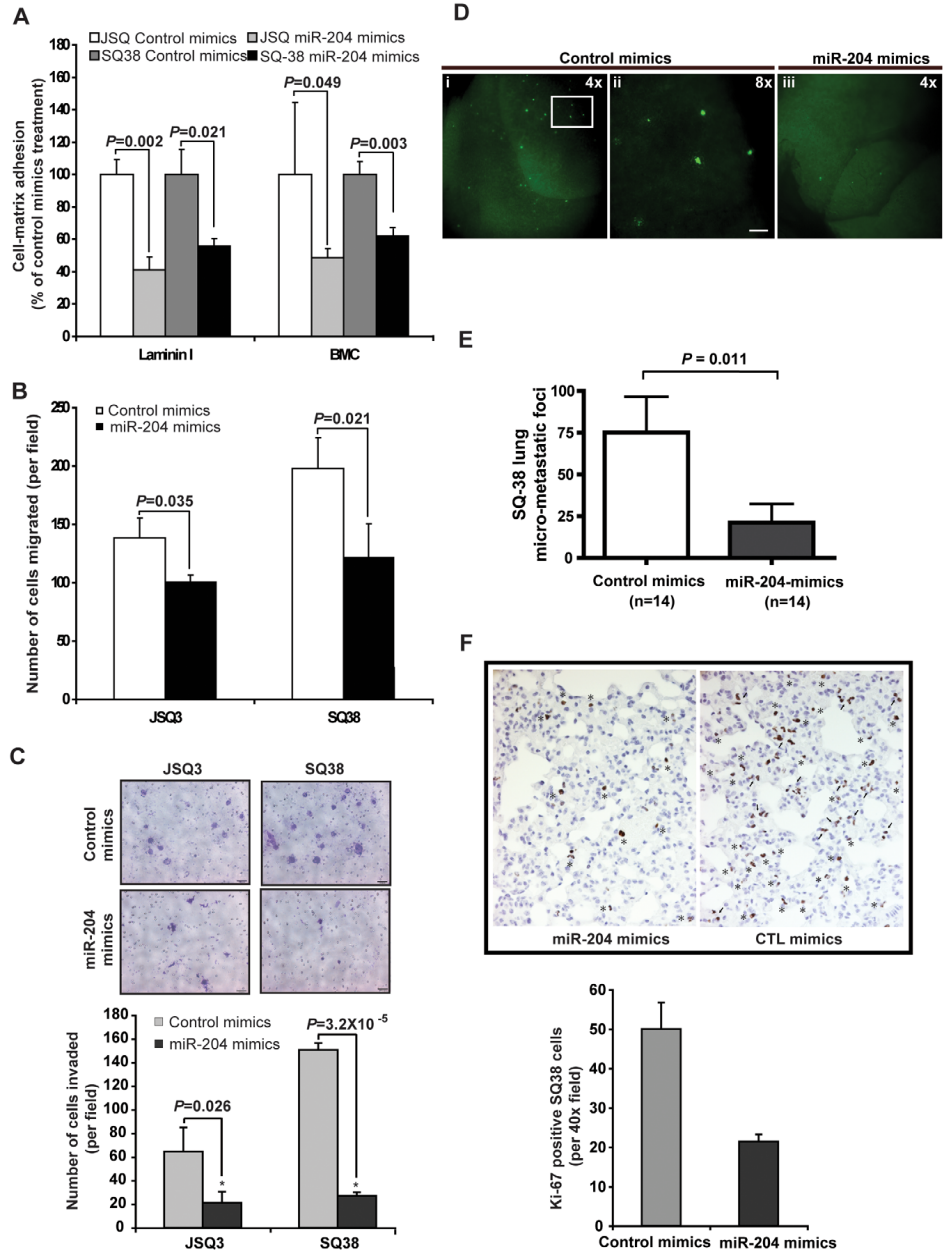
miR-204 suppressed HNSCC cell migration, adhesion and invasion *in vitro* and lung colonization *in vivo*. (**A–C**) Ectopic enhancement of miR-204 function inhibited JSQ3 and SQ38 adhesion to laminaI or basement membrane complex (BMC) (**A**), migration through the porous membrane in Transwell (**B**), and invasion through Matrigel (**C**). Triplicate repeats were conducted at experimental point for **A** (Methods). For **B** and **C**, cells migrated to the basal side of the porous membrane was visualized with a Zeiss Axiovert microscope at ×20 magnification. 10 random fields from three replicate wells were counted and the number of cells that had migrated or invaded was presented as number of cells counted per field of the porous membrane (Materials and Methods). Error bars represent mean±standard error of the mean (SEM); *P*-*values* were obtained using a one-tail t-test with unequal variance. (**D–E**) Restoration of miR-204 function by miR-204 mimics treatment significantly attenuated GFP-SQ38 tumor lung colonization. Total number of GFP-SQ38 lung surface foci was counted lobe by lobe using a Leica fluorescent stereoscope under 4× magnification [**D(i)** and **E(iii)**] in a total of 28 mice. A magnified view (8×) of the insert in **E(i)** is shown in **D(ii)** (top). Scale bar: 100 µm, Error bars represent mean±SEM. (**F**) Restoration of miR-204 function significantly decreased Ki-67 positive SQ38 cells in the lung. Ki-67 positive cells in each section were counted in 10 randomly chosen fields (40×) and six specimens in each experimental group were used (left panel). Error bars represent mean ± SEM; In (**E**) and (**F**), *P*-*values* were calculated based on unpaired one-tailed Mann-Whitney test. The image is a representation of a microscopic field (right panel). * indicates single-cell GFP-SQ38 foci; arrows indicate multi-cell GFP-SQ38 foci.

Among miR-204 gene targets that are potential regulators of cell-matrix interaction and proteolysis, overexpression of APRC1B [34], CTSC [35], FAP [36], MMPs [37], BMP1 [38], CDH11 [39] and ITGB4 [40] is associated with cancer metastasis and/or poor prognosis. Therefore, we evaluated the role of miR-204 in HNSCC tumor progression. For these studies, we selected JSQ3 and SQ38 HNSCC cell lines for *in vitro* and SQ38 for *in vivo* characterization. The two cell lines were derived from nasal cavity and sinus HNSCC tumors, respectively (Table 5 in Text S2) [26]. *In vitro*, ectopic restoration of miR-204 function by miR-204 mimics had no effect on the viability and proliferation of the two cell lines (Supporting Figure 6 in Text S1). In contrast, increased miR-204 function led to a significant inhibition (*P*<0.05) of the ability of JSQ3 and SQ38 cells to adhere to laminin-rich basement membrane (Figure 4A), to migrate through porous Transwell (Figure 4B), and to invade through Matrigel-coated basement membrane (Figure 4C). These results demonstrate that increased miR-204 function via its synthetic mimics is sufficient to suppress cell-matrix interaction, motility and invasiveness *in vitro*.

To assess whether miR-204 could inhibit HNSCC tumor metastasis *in vivo*, we increased miR-204 function in SQ38 with miR-204 mimics treatments for three days prior to tumor transplantation. We employed an experimental model of lung metastasis by tail vein injection of tumor cells allowing characterization of tumor cell extravasation and colonization in the lung. For conducting *in vivo* fluorescent imaging analysis, we generated SQ38 cells stably expressing high levels of GFP fluorescent protein (Materials and Methods). To initiate the study, one million of GFP-SQ38 cells transfected with either control mimics or miR-204 mimics were transplanted into athymic mice via tail-vein injection. GFP-SQ38 micrometastatic foci developed in the lung over a period of three weeks were scored lobe by lobe for each freshly isolated lung under fluorescent stereoscope (Materials and Methods). Control mimics-treated SQ38 cells efficiently extravasated, established micro-metastases in 100% of animals and produced a mean number of lung metastatic foci of 75 on the whole lung surface. In drastic contrast, 50% of animals (7 out of 14) receiving miR-204 mimics treated SQ38 cells failed to develop any lung metastasis (Figure 4D–E and not shown), while the other 50% of animals developed significantly less GFP-SQ38 lung foci at this early three-week time point (*P* = 0.011 Figure 4E). Moreover, consistent with the predicted role of miR-204 targets AURKB and CDC25B as hub/bottleneck regulators of the cell cycle sub-network (Figure 3), restoration of miR-204 function *in vivo* significantly decreased the number of Ki-67 positive proliferating single SQ38 cells (indicated by *) and micro-foci (indicated by arrows) in the paraffin embedded lung sections (*P* = 0.001, Figure 4F). Moreover, Ki-67 positive SQ38 cells that received miR-204 mimics treatment were mostly single-cell foci and were in striking contrast to the multi-cell foci observed in the lungs of control mimics treatment group (Figure 4F). Taken together, these observations indicate that miR-204 can significantly suppress experimental lung metastasis of SQ38 HNSCC tumors, thereby acting as a potent suppressor of metastasis.

The novelty of our illustration of metastatic suppressor functions of miR-204 in head and neck cancer and its relevance to metastasis stems from our demonstration of miR-204 function at multiple scales of biology that collectively show its potential as a key regulator microRNA. Definitive demonstration of the role of miR-204 in head and neck progression requires future studies using cohorts of head and neck tumors.

### Expression pattern of 19 miR-204 targets identified a subtype of HNSCC tumors exhibiting an EGFR-pathway signature and predicted earlier relapse

**Figure 5:**
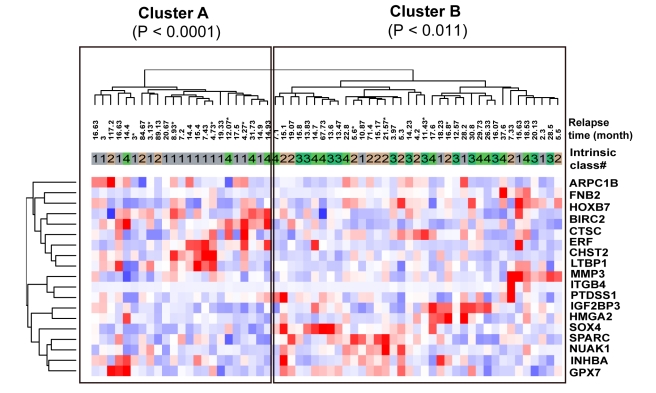
Expression pattern of miR-204 targets identified a subtype of HNSCC tumors exhibiting an EGFR-pathway signature and miR-204 was deregulated in other squamous and epithelial tumors. miR-204 functional targets classified 60 HNSCC tumors in (GSE686) [41] microarray based on their intrinsic properties (Methods). *P*-*values* were obtained using a Fisher's exact test; *: censored data.

To explore the clinical relevance of miR-204 down regulation in HNSCC, we conducted an unbiased hierarchical clustering analysis of 60 HNSCC tumors harvested from representative anatomical sites of HNSCC in GSE686 [41] based on the mRNA expression pattern of 34 miR-204 targets identified in GSE6631 [21] (Materials and Methods). The original study reported a 582-gene signature set in GSE686 that classified this set of tumors into four distinct groups: (1) an EGFR-pathway signature subtype, (2) a mesenchymal-enriched subtype, (3) a normal epithelial-like subtype, and (4) a subtype with a high level of antioxidant enzymes [41]. Hierarchical clustering using 19-upregulated genes, a subset of miR-204 targets that could be mapped to this dataset, identified two clusters (Figure 5). Tumors in Cluster A were enriched with the EGFR signature and correspond to Group 1 of the classification of Chung et al. (*P*<0.0001). In comparison, tumors in Cluster B were enriched with the Group 3 “normal epithelium-like subtype” tumors (*P*<0.011) [41]. This is consistent with our observation that miR-204 targets were hub-bottleneck regulators of an EGFR-dependent regulatory network in HNSCC (Figure 3). Further, consistent with the prognostic capability of a 582-gene signature set reported by Chung et al. [41], Cluster A showed overall earlier relapse than Cluster B (Figure S7). The fact that very comparable prognostic predictions can be derived using only 19 miR-204 gene targets suggest a potentially important role of miR-204 in HNSCC prognosis and merits further investigation and validation using a larger cohort of HNSCC tumor samples with well-characterized clinical outcomes.

## Discussion

Here, we developed an efficient combined computational and biological approach to predict and to prioritize cancer microRNAs for biological investigation. We demonstrated this strategy as an effective economical alternative to comprehensive microRNA analysis in cancers such as HNSCC for which prior genomic array datasets (mRNA or microRNA) are less abundant. This approach also allowed the identification of functional gene targets of the deregulated microRNAs that would otherwise require paired profiling of mRNA and microRNA expression for which the feasibility is often limited by the additional costs, or by the lack of access to the tissue. Employing this method that integrates the analysis of microRNA target predictions, differential HNSCC gene expression and the cancer genes in the OMIM genetic dataset, we identified and characterized miR-204, located within its host gene TRPM3 at the 9q21.1–q22.3 region frequently incurring allelic loss [11]–[15], as a potential tumor suppressor microRNA of HNSCC and possibly of other epithelial cancers. The high propensity of LOH at 9q21.1–q22.3 that occurs in 37% HNSCC pre-malignant conditions, further increases to 67% in cancer state [14] suggesting the presence of tumor suppressor gene candidates. While tumor suppressor genes at other frequent allelic loss loci in HNSCC have been identified, gene candidates responsible for the tumor suppressor activity associated with the 9q21 locus remain elusive. Here, we provided a plausible mechanism that loss of tumor suppressor function of miR-204 as a result of allelic imbalance at 9q21.1–q22.3 may significantly increases the genetic susceptibility to HNSCC oncogenesis and progression. LOH at this locus is also seen in the squamous cell carcinoma (SCC) of the esophagus [42] and SCC of the lung [43] suggesting a common somatic genetic lesion underlies the development of SCC of diverse tissue origin. The highly coordinated and nearly complete suppression of miR-204 and its host gene TRPM3 (Figure 1B–E) raises the possibility that TRPM3 mRNA expression may serve as a marker to indicate miR-204 expression status in HNSCC or other tumors, and also potentially LOH at 9q21.1–q22.3. Since a small variation in the expression of a specific microRNA is expected to affect the expression of tens or hundreds of target mRNAs, genetic variations in a microRNA expression at the chromosomal break point, as we observed with miR-204 at the 9q21.1–q22.3 locus, could represent an effective mechanism of cancer predisposition, a hypothesis that is supported by emerging experimental evidences [44],[45]. A few recent studies have reported genome-wide microRNA expression changes using HNSCC cancer cell lines [46]–[49] or tumor tissues [49]–[51]. While similar miR-204 downregualtion was reported in head and neck cancer cell lines based on microarray analysis [46],[48], its expression status was not further confirmed by PCR or other methods and its biological functions were not explored. Additionally, since its identification [24] biological characterization of miR-204 functions in normal development remain limited. Thus far, miR-204 was implicated in affecting global mRNA expression levels in the retina [52]; and was shown to regulate mesenchymal progenitor cell differentiation [53].

Through enrichment analysis and network modeling using mRNA gene expression profile, we identified a set of functionally related miR-204 targets that showed increased mRNA expression in HNSCC upon miR-204 suppression (Figures 2A–C). The presence of miR-204 binding sites (Table 8 in Text S2), the coordinated up-regulation and the ability of increased miR-204 function to specifically inhibit the expression of 18 out of 21 gene targets (86%) (Figure 2D, Supporting Figures 4–5 in Text S1) suggest that these predicted genes are very likely selective and direct miR-204 targets in HNSCC. This finding is consistent with the genome-wide association between microRNA binding sites and the ability of corresponding targeting microRNAs to alter their gene expression [54]. This is the first report of a large set of functionally related cancer microRNA targets that was identified via high throughput computational approaches and confirmed biologically. In addition, the joint analyses of sequence-base information and mRNA expression arrays yielded an accuracy rate of 86% of miR-204 target predictions which surpasses the published accuracy (about 40%) of each sequence-based method when used alone [10],[55],[56].

More broadly, we demonstrated a computational framework for predicting altered regulatory networks and biological functions associated with differentially expressed microRNA targets. Indeed, our combined systems biology approach uncovered previously unknown connections between microRNA regulation, network topology, and expression dynamics for which we obtained thorough biological validations. While genome-scale analyses of interactions among microRNA gene targets in the context of a cellular or protein-protein interaction networks have been conducted computationally [57]–[59], such methods and observations await biological confirmation. Here we significantly extended the observations of two recent reports on network modeling [31],[32] and demonstrated the feasibility and validity of deploying statistical and bioinformatics approaches to derive regulatory networks corresponding to altered expression of proteins targeted by microRNAs (Figure 3). Further, combining functional enrichment analysis with network modeling leads to the unbiased prioritization of an EGFR-dependent protein regulatory network connected via up-regulated gene targets of microRNAs in human HNSCC (Figure 3C). Topological analyses of hub and bottleneck properties further identified key regulatory proteins within the EGFR network (Figures 3A–B). miR-204 appeared critical to regulate the function of this “prioritized HNSCC PPIN” as its gene targets exhibited significant enrichment of hub and bottleneck properties (Figures 3A–B). Since the EGFR network was derived from overexpressed genes in HNSCC, the functional enrichment of its 56 proteins suggests their positive regulation of cell cycle, cell/matrix adhesion and extracellular matrix modeling. Using this approach, the biological effect of altering the function of a specific microRNA, such as miR-204, can be accurately predicted via its gene targets that are key regulators of a protein network. Accordingly, enhancement of miR-204 function inhibited the expression of its functionally related gene targets (Figure 2D, Supporting Figures 4–5 in Text S1) in the “prioritized HNSCC PPIN” and lead to the reduced adhesion, migration and invasion *in vitro* (Figures 4A–C) and experimental lung metastasis *in vivo* (Figures 4D–F). Further, the strong association of overexpression of functional miR-204 gene targets with an earlier relapse in a sub-type of HNSCC tumors expressing an EGFR-pathway signature (Figure 5) suggests that miR-204 expression and its deregulated gene targets could be potentially used for mechanism-based prognostic stratification of HNSCC patients to complement the conventional clinical-pathological tumor diagnosis. In fact, the feasibility of employing microRNA as sensitive and informative biomarkers for molecular diagnosis has recently been demonstrated [60].

Collectively, these findings show that single protein network modeling and statistical functional enrichment of a PPIN can illuminate altered complex biological processes and regulatory pathways associated with microRNA dysfunction in cancer with high precision. Complementary approaches have been developed to analyze gene expression changes in the molecular and biological context for candidate gene prioritization and for deriving mechanistic understandings that are most relevant to cancer biology [61]–[64]. The system's properties and microRNA-regulated molecular networks we discovered could be exploited for the design of “network mechanism”-based therapies to specifically restore tumor suppressor microRNA functions as an alternative to the single-gene target paradigm and merits further investigation.

## Materials and Methods

### Ethics statement

All animal works have been conducted according to IACUC guidelines and were approved at the IACUC committee at the University of Chicago. All research involving human participants have been approved by the authors' institutional review board. Informed consent has been obtained.

### Gene expression analysis of microarray data and subsequent statistical analyses (Figure 1A, Figure 1G, Figure 5 and Supporting Figure 3 in Text S1)

Microarray datasets were downloaded from NCBI GEO database. The .cel file of HNSCC mRNA transcription array sets GSE6631 [21] and GSE2379 [22] were processed using the Bioconductor Package [65] implementation of GCRMA in R Software [66]. To identify differentially expressed genes, SAM analysis [67] was performed using paired T-test between the HNSCC tumor and its corresponding paired normal tissue obtained from the same patient. The criteria for gene selection were fold change ≥2 and False Discovery Rate (FDR) ≤0.0006 (Figure1A and Supporting Figure 3 in Text S1).

The association of miR-204 targets with clinical parameters was analyzed using HNSCC mRNA array set GSE686 [41]. The intensity ratios of red to green channel of the predicted miR204 targets were retrieved from GSE686 dataset. Missing values were assigned a constant value of 0. Redundant probes representing an identical gene were reduced to a single one using the mean expression value. The miR-204 targets predicted in Figure 2A and filtered by coefficient of variation >0.3 were used for hierarchical clustering. In Figure 5, the two-way hierarchical clustering was conducted with the dChip software using its default parameters (distance metric: 1-Pearson correlation; centroid linkage clustering) [68], while the significance of the association between the hierarchical clusters and molecular groups of HNSCC samples [41] was determined by two-tailed Fisher's exact test adjusted with Bonferroni correction. The sample information file was obtained from the Table S1 of Chung et al [41]. The time to recurrence (termed relapse time), shown in Supporting Figure 7 in Text S1, was analyzed with the Kaplan-Myer method using the Logrank test of GraphPad Prism software (version 4) [69], and right censoring was conducted for subjects alive at the end of the study (subjects identified by “*” in Figure 5).

To determine the miR-204 expression status in epithelial tumors (Figure 1G), the expression values of miR-204 were extracted from microRNA array set GSE2564 [18]. Only six solid tumor types, colon, kidney, prostate, uterus, lung and breast that contained more than one samples in both tumor and the respective norm tissue were included in the analysis. Comparisons between tumors and their respective normal tissues were performed by unpaired two-tail t-test with unequal variances.

### Prediction of HNSCC down-regulated microRNAs from public gene expression profiles and putative microRNA target genes (Table 1 in Text S2, Table 9 in Text S2)

#### Generation of an integrated human microRNA target database-*miRNOME*

We generated an integrated and comprehensive human microRNA target database, “miRNOME”, by merging five microRNA target datasets: TargetScan [10], PicTar4way [70], miRBase [56], miRanda [55] and TarBase [71] (integration details provided in Table 9 in Text S2). Downloaded versions are described in Table 1 in Text S2. miRNOME contains 534 distinct human microRNAs, 17,343 predicted putative microRNA gene targets and 444,558 distinct microRNA-Target relationships. Specifically, 5110 distinct genes in GSE6631 and 5131 distinct genes in GSE2379 are respectively targeted by 531 and 530 microRNAs in the miRNOME.

#### Imputed microRNA regulation based on weighted ranked expression and putative microRNA targets (IMRE) (Figure 1A, Supporting Figure 2 in Text S1, and Table 2 in Text S2)

We developed a novel prioritization method (Supporting Figure 2 in Text S1) to predict microRNA regulation from genome-wide gene expression and microRNA putative targets predicted by the miRNOME database. Using the expression of putative targets of microRNAs in miRNOME, we calculate a *P-value* for each microRNA representing their potential deregulation between the cancer and normal tissue conditions. The method development and procedures for conducting IMRE analysis are detailed below:

*Filtering*. After GCRMA normalization, about half the genes were filtered out according to the following criteria: i) probes whose average expression intensity are below the average background intensity, or ii) the probes whose inter quartile range (**IQR**) are lower than the median of the rest probe-set's IQR because they are less likely to be differentially expressed, and/or iii) to control for the bias of multiple probes per gene, the probe-sets with the largest IQR value were retained and the others were removed for the genes with multiple probe-sets.

*Expression processing*. In sample *j* containing a total of *G* genes, each gene *x* is ranked by expression as 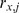 ∈ {1, 2…*G*}, and scored according to an exponential weighted 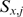 (**Equation 1**) using an approach that we previously described to compare gene expression lists (*OrderedList* [72] method of Bioconductor [73]).
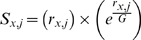
(1)


*Prediction of microRNA target regulation*. The contribution of a microRNA 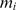 regulation of gene expression to a single sample *j* is imputed by calculating the difference between the centroid of weighted rank expression (**WRE**) of its targets according to the miRNOME (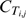, Equation 2) with that of non-targeted genes (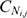, Equation 2), referred to as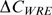 (Equation 3); where T***_i,j_*** is the target gene-set of microRNA (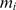), and 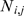 is the non-target gene-set of this microRNA. Further, the scores are adjusted for the cardinality (count of genes) of each gene-set (*e.g.* cardinality of T***_i,j_*** is | T***_i,j_*** |; Equation 3). 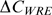 follows a normal distribution in both GSE6631 and GSE2379 (data not shown). An empirical Student T-test for unequal variances was performed to compare Δ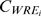 for each microRNA 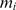 between cancer and normal tissue (Bioconductor package *twilight*, 1,000 permutation resampling, “paired t-test”for GSE6631, “unpaired t-test” for GSE2379 [74]). To adjust for multiple comparisons (different microRNAs on the same dataset), we calculate a Benjamini and Hochberg false discovery rate (**FDR**) from the *P-values* of Equation 4 and used a 5% threshold for significance [75]. The IMRE algorithm written in R language and bioconductor is made available at http://www.lussierlab.org/IMRE.
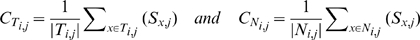
(2)

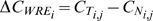
(3)


### Prediction of microRNAs deregulated in cancers from enrichment analysis of inheritable cancer genes in OMIM (Figure 1A, and Table 3 in Text S2)

MicroRNAs most likely to regulate a large number of specific inheritable cancer genes were predicted using an enrichment statistics. The Online Mendelian Inheritance in Man (**OMIM**) [76] is a semistructure database in which we computationally coded cancer genes (OncoMIM, Protocol S1/Section A) to a clinical nomenclature and mined with statistical enrichment to predict microRNAs that could deregulate a large number of genes, each associated with a certain type of cancer. OncoMIM contains 610 biologically validated or clinically demonstrated inheritable cancer genes among which 586 (96%) are predicted targets of 527 microRNAs in the miRNOME, from which we can calculate significantly enriched microRNAs.

The cumulative hypergeometric distribution (**Equation 4**) was applied to identify significantly enriched microRNAs. We calculated the *P-values* based on the **Equation 4** with the following variables: *N* is the number of OMIM genes also found in the miRNOME (3232 for anatomy, 2181 for disease), *M* is the number of genes associated to a specific cancer term in OncoMIM and also targeted by any microRNAs in the miRNOME, *n* represents the number of genes targeted by a specific microRNA in the miRNOME and also found in OncoMIM associated to any cancer term, *m* is the number of genes associated to both a specific cancer term in OncoMIM and to a specific microRNA in the miRNOME (*m = M∩n*).
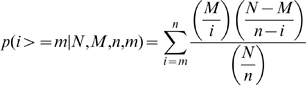
(4)


To control *p* in **Equation 4** for multiple comparisons, we applied the Bonferroni-type adjustment method known as Šidák single-step adjusted *P-value* for multiple comparisons (**Equation 5**) [77]. Significant correlations are first refined to remove false positive signals inherited in the hierarchies of the clinical nomenclature (Protocol S1/Section B) and then adjusted *P*-*values* (*p′*) are less than 0.05 (*n* = number of comparisons, *p* taken from **Equation 4**)
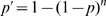
(5)


### Refinement of the hierarchical *P-values* in enrichment of GO or of SNOMED terms (Supporting Figure 8 in Text S1, Protocol S1/Section B)

We developed an algorithm to identify and filter out false positive *P-values* derived from enrichment studies in ontologies (hierarchical classifications) due to the inheritance of genes in ancestry classes of a significantly enriched class [78],[79] (Supporting Figure 8 in Text S1, Protocol S1/ection B).

### Review of literature of deregulated microRNA in cancer (Supporting Figure 3 in Text S1, Table 4 in Text S2)

A gold standard of microRNAs deregulated in cancers was derived from the literature and was used to evaluate microRNA predictions in from OMIM cancer genes (Supporting Figure 3 in Text S1).

### Prioritized HNSCC PPIN using Single Protein Network Modeling in a protein-protein interaction network (Figure 3 and Tables 10–12 in Text S2)

#### Datasets used to construct the protein-protein interaction network (Figure 3, Table 10 in Text S2, Protocol S1/Section D)

The protein-protein interaction network (**PPIN**) was generated by integrating seven protein interactions and signaling datasets. Protein interactions from each dataset were standardized to a two-column list of pair wise interactions between SwissProt accession IDs, with an additional column providing the source dataset and references to the literature when available. An overview of the seven datasets used in this study is provided in Table 10 in Text S2, while the details of the integration process is provided in Protocol S1/Section D.

#### Generation of protein-protein interaction network

Homo sapiens data was retained from BIND, BioGRID, DIP, HPRD, KEGG, MINT, and Reactome. Postulated interactions in Homo sapiens based on Yeast Two-Hybrid experiments were excluded. Identifiers were converted to a common SwissProt standard coding using translation tables from HGNC (Table 9 in Text S2) and the data sources' own cross-mappings.

#### Conservative permutation re-sampling of the PPIN

Permuted PPIN networks were generated using a link randomization approach [80]. Proteins are considered as nodes, and interactions between proteins are links. Since biological networks are scale free rather than random [81],[82], link-randomization can create conservative “permuted networks” as controls, from which we can derive an empirical distribution of interactions between a subset of proteins. Furthermore, our implementation of a link-randomization conserves the number of “connections” of each protein (node-degree [81]–[83]). Thus the scale free properties of the original distribution are preserved in every permutation as well as the node degree of each specific protein, while the interactions (links) between these proteins vary. Self- interactions, such as those formed by homomultimers, were ignored to avoid introducing bias into the network. Duplicate protein interaction pairs were also excluded in the permutation. 10,000 of these permuted networks were generated from the original amalgamated interaction network consisting of real datasets.

#### Single Protein Network Modeling and Prioritized HNSCC PPIN (Figure 3 and Tables 11–12 in Text S2)

Additionally, we developed a model that estimates the probability of occurrence of an observed Single Protein Network arising from the upregulated gene list between HNSCC and normal paired tissue in GSE6631. Each of these unregulated HNSCC gene was translated to its corresponding protein identifier in the network (HNSCC protein). Each HNSCC protein was mapped to each of the rest HNSCC proteins according to existing pairs of protein interactions in the original PPIN yielding an Observed number of distinct Protein Interactions (Observed count of PI). Thereafter, the same procedure was applied to the 10,000 permuted PPINs yielding control counts of distinct protein interactions for each of the UG (Control count of PI). Since each HNSCC protein had a constant node degree in each permutation (see the previous paragraph), this procedure controlled properly for HNSCC proteins having more protein interactions than others thus providing no statistical advantage to those better connected proteins (such as hub or bottleneck proteins). For each HNSCC protein, a *P-value* was assigned by measuring the frequency at which the “Observed count of PI” of that HNSCC protein occurred in the empirical distribution of 10,000 “Control count of PI” for these specific HNSCC proteins (Table 11 in Text S2). Each HNSCC proteins were subsequently ranked according to its *P-value*. At each cutoff *P-value*, a certain number of HNSCC proteins were prioritized. Consequently, a FDR of the prioritized HNSCC proteins (FDR of prioritized proteins) was calculated by dividing the median number of proteins prioritized at that cutoff in the empirical distributions of permuted PPINs divided by the observed number of prioritized HNSCC proteins in the real PPIN. We refer to this approach as single protein analysis in the network (SPAN).

A similar procedure was developed to calculate the FDR over a pair of protein interactors among the observed prioritized HNSCC proteins (FDR of links). A “Prioritized HNSCC PPIN” (Figure 3) was predicted from SPAN in the “genome-scale PPIN” with a FDR of 7.14% for the links between labeled genes and of 10.15% for upregulated HNSCC genes in GSE6631. The resulting network was drawn using Cytoscape [84]. Details on the protein interaction dataset supporting each pair of protein interactions are provided in Table 12 in Text S2. Hubs in the PPIN are defined as the top 20% of proteins' node degree (grey nodes in Figure 3A). Similarly, the bottlenecks (grey nodes in Figure 3B) are defined as proteins are the top 20% betweenness score calculated using the “betweenness.c” program we developed (http://www.gersteinlab.org/proj/bottleneck/) [30]. 10.4% of the PPIN proteins were observed to have both hub and bottleneck properties. Enrichment studies of hub, bottleneck and hub-bottleneck proteins presented in Figure 3 have been conducted using one-tailed cumulative hypergeometric distribution.

#### Enrichment of Hub and Bottleneck Proteins in the HNSCC PPIN associated with microRNA targeting (Figure 3A–B)

To determine whether microRNA targets in the HNSCC PPIN exhibit the genome-wide systems' properties of “hub” and “bottleneck” and their enrichment, we calculated the proportion of hub and of bottleneck proteins among microRNA targets present in the HNSCC PPIN for each microRNAs in the miRNOME. Thereafter, we conducted a non-parametric Wilcoxon Signed Rank Test comparing this frequency with the theoretic expectation that derives from a random draw (as defined in the previous paragraph: 20%).

Using the miRNOME, we also calculated the number of distinct microRNAs that could potentially target the genes encoding for each protein of the PPIN. We subsequently obtained enrichment statistics of the hub and bottleneck properties related to microRNA regulation by comparing the count of microRNAs between hub proteins and non-hub proteins, between bottleneck and non-bottleneck proteins, as well as between hub-bottleneck and non hub-bottleneck proteins using the non-parametric Mann Whitney test. Calculations of these statistics were conducted with the GraphPad Prism software (version 4) [69].

### Functional enrichment analysis of miR-204 targets and PPIN using Gene Ontology (Figures 2B–C, Figure 3C, Table 7 in Text S2, Protocol S1/Section C)

To provide insights into biological functions and processes potentially regulated by miR-204 in HNSCC, we conducted standard statistical enrichment analyses based on the functional assignments of gene in Gene Ontology (GO) [28] to infer significantly deregulated functions associated with altered miR 204 target expression in the HNSCC according to their presence in the miRNOME and/or the PPINs (details in Protocol S1/Section C).

### Generation of Head and neck cancer cell lines and cell culture

We previously established 10 low passage human head and neck squamous cell carcinoma lines (HNSCC) (SCC25, SCC35, SCC58, SCC61, SCC135, SCC151, SQ20B, SQ38, and JSQ3), from head and neck tumor specimens of different head and neck primary sites [26]. This panel of cell lines was established from head and neck tumor specimens of different primary sites and most of the patients quickly developed local failure and eventually died of the disease [26]. Nu61 was derived from SCC61 tumors that developed radioresistance after serial passage and radiation treatment *in vivo* [85]. All cell lines were cultured and maintained in 1∶1 DME/F12 supplemented with high glucose and 10% fetal bovine serum. GFP-SQ38 cells were established via retroviral-mediated gene transfer using pLEGFP-N1 retroviral vector (Clontech).

### Quantitative RT-PCR Analysis of microRNA and mRNA expression (Figure 1B–F, Supporting Figures 4–6 in Text S1, and Table 13 in Text S2)

Total RNA from normal and tumor tissues of esophagus, lung and cervix were obtained from the Ambion FirstChoice collection of RNA that is compatible with both mRNA and microRNA analysis.

Total RNA from HNSCC cell lines, tumors and normal tissues was extracted and purified using TRIzol (Gibco/BRL) according to manufacturer's instructions. Tissues from primary HNSCC tumors were obtained from surgical procedures performed at our institution. Samples were snap frozen immediately in liquid nitrogen and stored at −80 °C. Laser micro-dissection was performed on frozen sections and approximately 10,000 cells were captured for RNA extraction. Normal buccal mucosa was obtained from healthy volunteers with no history of smoking and drinking according to an approved open IRB protocol.

miR-204 expression was measured using TaqMan MicroRNA quantitative PCR (qPCR) assay (Applied Biosystems) according to manufacturer's instructions.. Real-time PCR was carried out using the Applied Biosystems 7900 Sequence Detector System (Applied Biosystems). All qPCR reactions were run in triplicate. Human TATA-binding protein (TBP) (Applied Biosystems) was used as an endogenous control for miR-204 expression normalization. The fold changes of miR-204 expression between normal and tumor tissues or cell lines were calculated using the ΔΔCt method of relative comparison.

For mRNA expression quantification, First-strand cDNA synthesis was carried out as above described except that random primers were used for reverse transcription (High Capacity cDNA Reverse Transcription Kit, Cat#4368814). Amplification of predicted miR-204 targeted genes was performed by Sybr Green qPCR assays using custom designed primers. Specific primers for each gene were designed using Invitrogen D-LUX Designer (https://orf.invitrogen.com/lux/) and sequences provided in **Table 13 in Text S2**. The mean Ct (cycle threshold) was calculated from the triplicates and used for the calculation of RQ values. qPCR condition for each gene was optimized that so that the standard error among the triplicates was <0.15 Ct. TBP was also used as endogenous control for data normalization. The fold changes of target gene were calculated using the ΔΔCt method of relative comparison. In addition, as negative controls for the off target effect of miR-204 mimics treatment, real time qPCR was performed to include three additional endogenous controls: PPIA (AB, Cat#4333763), GUSB (AB, Cat# 4333767) and, HPRT1 (Cat#4333768) using commercially designed Taqman gene expression assays (Applied Biosystems). Quantitative mRNA expression data were acquired and analyzed in either 96- or 384-well-plate format using an Applied Biosystems 7900 Sequence Detector System (Applied Biosystems).

### Increase miR-204 function by miRIDIAN mimics treatment (Figure 2D and Figure 4)

40% confluent JSQ3 and SQ38 cells were transfected with 50–200 nM Control [Cat#110CN-001000-01] or miR-204 miRIDIAN mimics [Cat#110C-300069-02](Dharmacon) using Oligofectamine (Invitrogen). Transfection efficiency was optimized and estimated to be >90%. Proliferation assay, cell adhesion assay. Migration assay and Matrigel invasion assay were conducted at 72 hours after transfection. *In vivo* tail-vein injection of mimics treated GFP-SQ38 cells was performed at 48h after transfection.

### InnoCyte ECM Cell adhesion assay (Figure 4A)

Cell adhesion was measured using the InnoCyte ECM cell adhesion assay kit (Calbiochem, Cat#CBA025) according to manufacturer's instructions. Control or miR-204 miRIDIAN mimics treated JSQ3 and SQ38 cells were trypsinized and re-suspended in fully supplemented medium. 20,000 cells and 15,000 cells were added to each well for JSQ3 and SQ38 cell lines, respectively. Cells were incubated for 2h at 37 °C. The plates were then washed with PBS to remove non-adherent cells. 100 µl Calcein-AM was added to each well, incubated with cells for 1h at 37 °C, and read with a fluorescent plate reader at an excitation wavelength of ∼485 nm and an emission wavelength ∼520 nm. Results were expressed as percent of cell adhesion compared to that of control mimics treated controls±standard error (SE) of 3 replicates.

### Trans-well migration and invasion assays (Figure 4B,C)

Control or miR-204 miRIDIAN mimics treated JSQ3 and SQ38 cells were trypsinized and re-suspended in fully supplemented medium. Cells were then seeded at 10,000 cells per well for migration assay or at 20,000 cells per well for invasion assay into trans-well inserts (8 µm pore size, BD Falcon). For invasion assay, the trans-well inserts were coated with 60 µg/45 µl/well of Matrigel (BD Falcon). Complete culture medium was used as chemo-attractant in the lower chamber. The assays were taken down with three PBS washes followed by fixation with 10% formalin and staining with 1% crystal violet after 6h for migration assay and 18h for invasion assay. The cells migrated to the basal side of the porous membrane was visualized with a Zeiss Axiovert microscope at ×20 magnification. 10 random fields from three replicate wells were counted and the number of cells that had migrated or invaded was presented as number of cells counted per field of the porous membrane.

### Determination of cell proliferation (Supporting Figure 6 in Text S1)

Cell proliferation assays were conducted in 96-well format by the MTT assay. Specifically, HNSCC cell lines were seeded at 5×10^3^ cells/well in 96-well plates and let incubated for 24 hours prior to treatment with control or miR-204 miRIDIAN mimics. After drug or siRNA exposure, 10 µl of MTT reagent was added to each well and incubated for 4h. The precipitates were dissolved in 100 µl of stop solution overnight and proliferation rate was determined by absorbance at 570 nm wavelengths with 690 nm as the reference wavelength using a spectrophotometer.

### Animal studies (Figure 4D)

Animal work was conducted in accordance with an approved protocol. Age and weight-matched (4–6 weeks old weigh 18–20 g) NCI athymic female mice were used for induction of experimental lung metastasis via the tail-vein injection of tumor cells. GFP-SQ38 cells were treated with miR-204 miRIDIAN mimics or non-specific control mimics for 2 days prior to tumor cell inoculation. 1×10^6^ viable cells were re-suspended in 100 µl of PBS and injected into the lateral tail vein. Metastatic colonization of lung by GFP-SQ-38 cells was determined at 3 weeks post tumor injection.

### Characterization and quantification of lung metastasis (Figure 4E)

28 Mice were sacrificed on day 21 after tumor cell inoculation. Lungs were perfused through tracheal with 2–3 ml of PBS, excised and then fixed in 10% formalin for 12 hours. Prior to fixation with formalin, lungs were examined under 4× magnification using fluorescent stereoscope (Leica) and scored lobe by lobe for GFP-SQ38 lung foci on the whole lung surface. Thereafter, the University of Chicago Immunohistochemistry Core Facility performed paraffin embedding, sectioning and H and E staining. 5-micron sections were stained with Ki-67 and Ki-67 positive SQ-38 cells or micro-foci were scored under 40× magnifications in 10 randomly selected fields for each section. A total of 6 lungs from each treatment group were examined.

## Supporting Information

Text S1 Supplementary Figures 1–8
(0.43 MB PDF)

Text S2 Supplementary Tables 1–14
(0.19 MB PDF)

Protocol S1 Supplementary Protocols (sections A–D)
(0.10 MB PDF)

Dataset S1 OncoMIM Table: Cancer-related terms in OMIM
(2.46 MB XLS)
