# Supplementary material for: Network Modeling Identifies Molecular Functions Targeted by miR-204 to Suppress Head and Neck Tumor Metastasis
Source: PLoS Comput Biol. 2010 Apr 1;6(4):e1000730. doi: 10.1371/journal.pcbi.1000730 (PMC2848541; doi:10.1371/journal.pcbi.1000730)
Supplement: Text S1 — Supplementary Figures 1–8 (0.43 MB PDF) [file pcbi.1000730.s001.pdf]

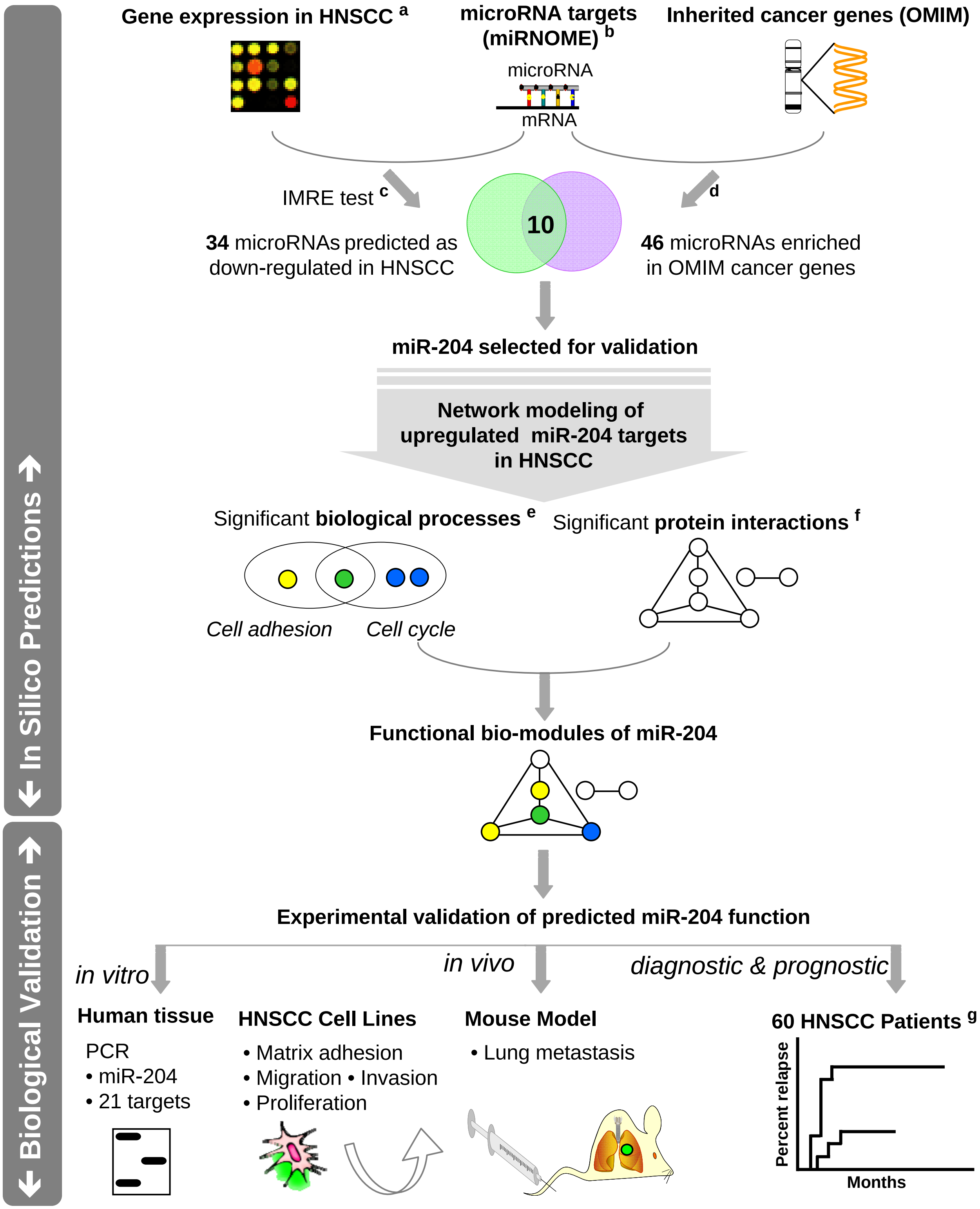

Supporting Figure 1. Outline for systematic computational approaches and biological experiments. **a** GSE6631 and GSE2379, **b** sequence-based microRNA target databases, **c** prediction of microRNA deregulated in HNSCC from weighted rank mRNA expression (IMRE), **d** cumulative hypergeometric distribution, **e** enrichment in Gene Ontology, **f** single protein network analysis, **g** GSE686.

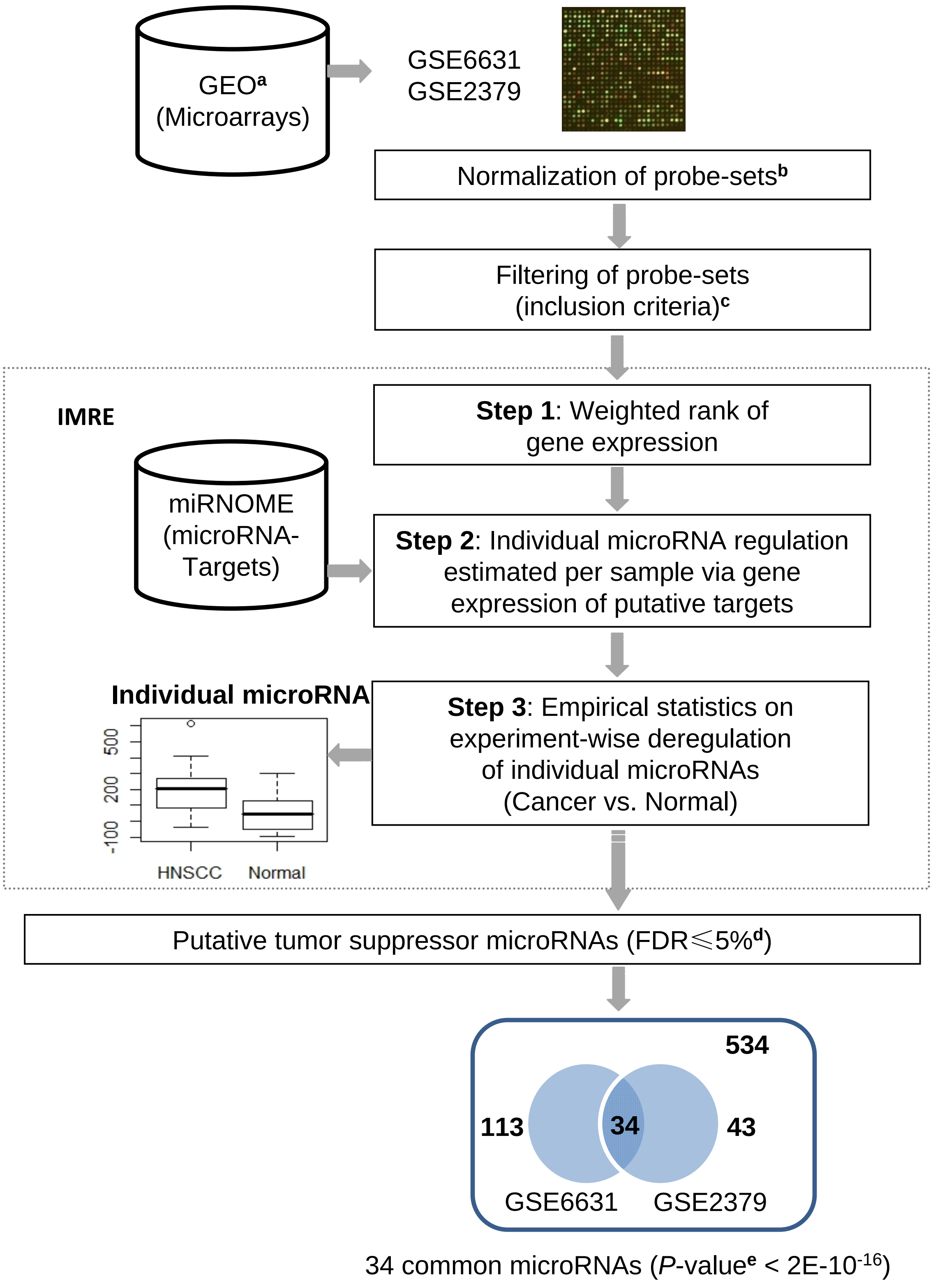

Supporting Figure 2. Illustration of predicted tumor suppressor microRNAs based on weighted rank expression and putative microRNA targets (IMRE, Methods, Figure 1A, Tables S2). **a**: HNSCC microarray, **b**: GCRMA normalization using R Software. **c:** filtering, resulted in 5110 genes targeted by 531 out of the 534 human microRNAs in the miRNOME; **d**: Empirical statistics (IMRE, **Methods**), **e:** Fisher's exact test.

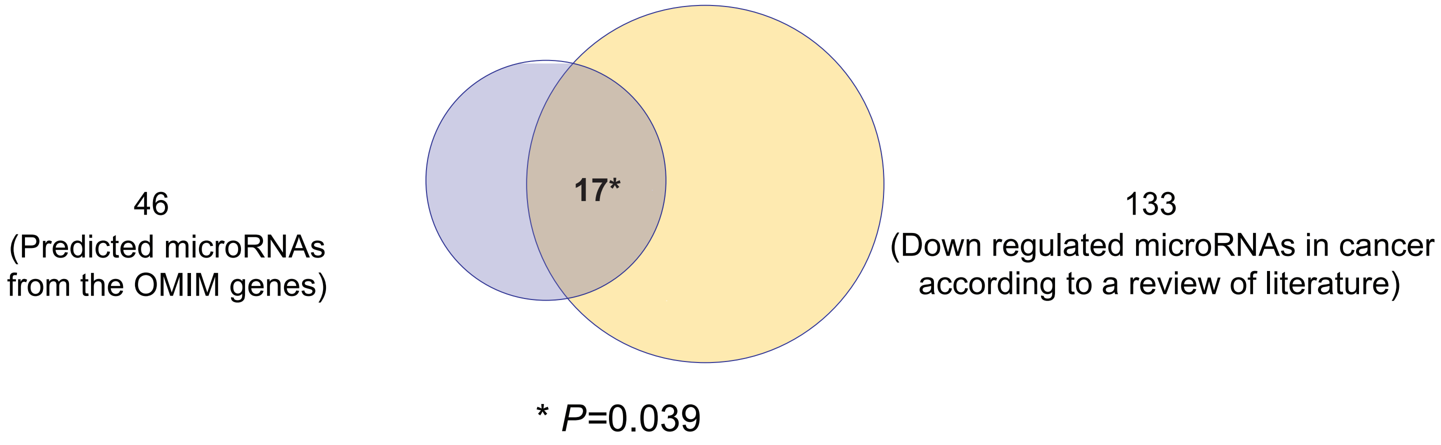

Supporting Figure 3. Evaluation of microRNAs predicted from OMIM cancer genes for biological validation using a review of literature. *P-value* was calculated by cumulative hypergeometric test.

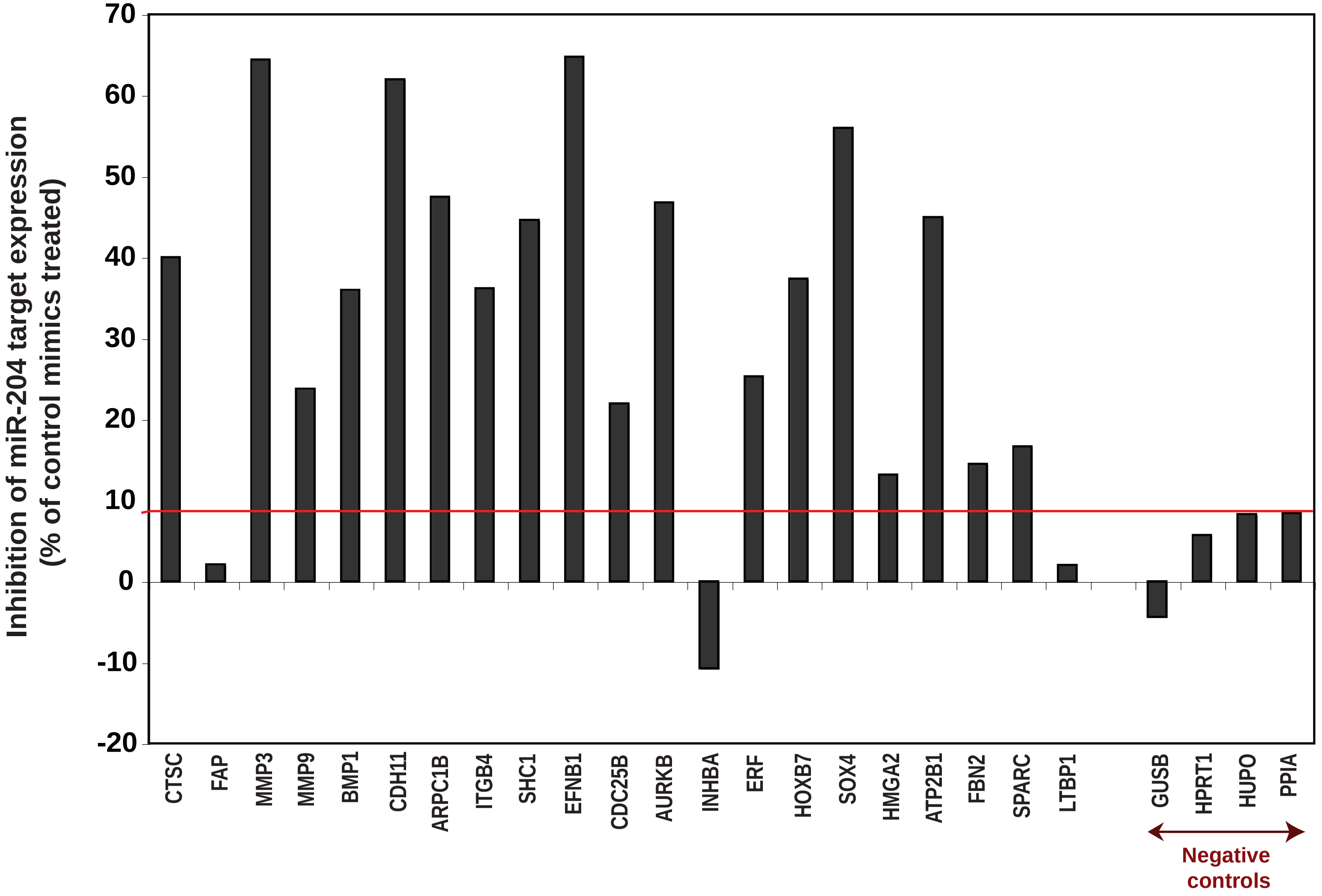

Supporting Figure 4. Restoration of miR-204 function by miR-204 miRIDIAN mimics inhibited its predicted mRNA targets expression in SQ38 HNSCC cell line. SQ38 cells were treated with 200nM of either control mimics or miR-204 specific mimics for 72h and total RNAs extracted with Trizol as described in **Methods**. Expression of predicted miR-204 targets were determined by qRT-PCR. TBP was used as an endogenous control for normalization. Triplicate real time PCR measurements were obtained and the mean PCR Ct was used to calculate RQ values. Standard deviation of the triplicate measurement was less than 0.15 PCR Ct. Shown are inhibition of miR-204 target mRNA expression levels upon miR-204 mimics treatment compared with that of control mimics treatment. While restoration of miR-204 function significantly inhibited the expression of 18 out of 21 predicted mIR-204 mRNA targets, the expression of 4 house keeping genes (negative controls) was largely unaffected upon miR-204 mimics treatment.
(Related to **Fig. 2D** of the manuscript).

A *JSQ3*

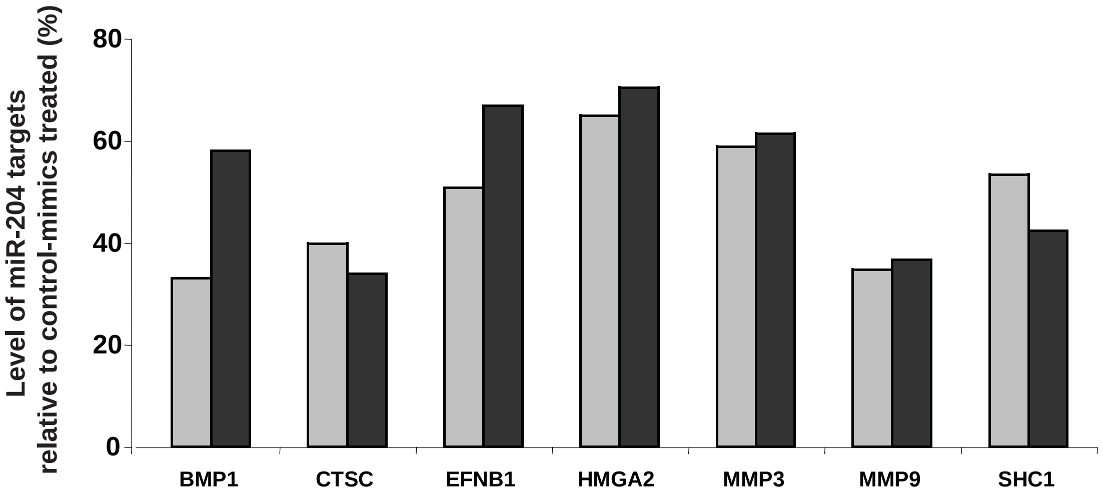

B *SQ38*

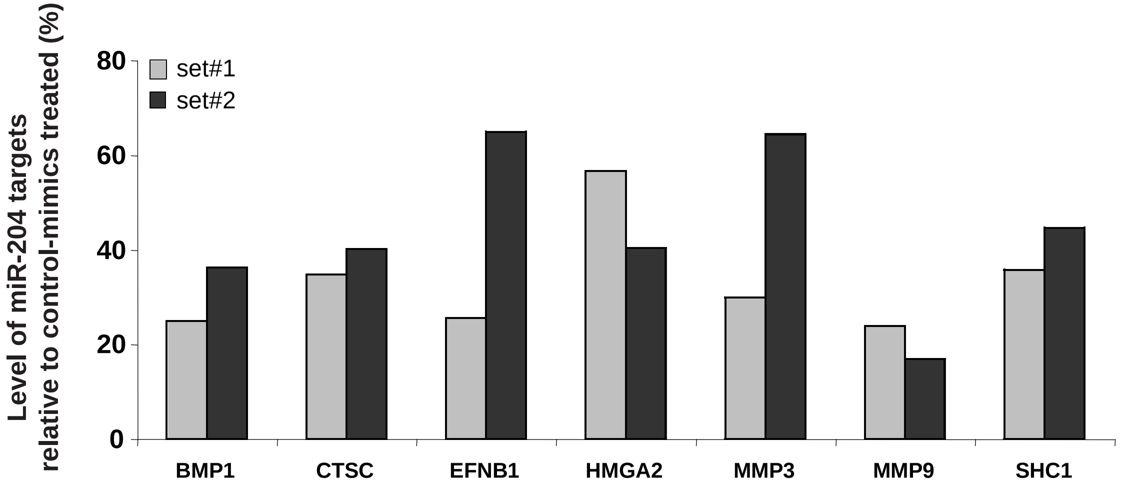

Supporting Figure 5. Predicted miR-204 targets were down-regulated to similar extent in JSQ3 and SQ38 cells treated with 200nM miR-204 miRIDIAN mimics in two independent sets of experiments. Total RNAs were extracted from JSQ3 and SQ38 cell lines with TRIzol as described in **Methods**. Expression of a sub-set of miR-204 targets were determined by qRT-PCR in two independent experimental repeats (Set#1 and Set#2). TBP was used as an endogenous control for normalization. Triplicate real time PCR measurements were obtained and the mean PCR Ct was used to calculate RQ values. Standard deviation of the triplicate measurement was less than 0.15 PCR Ct (cycle treshold). Shown are miR-204 target mRNA expression levels compared to control mimics treatment (Related to **Fig. 2D** of the manuscript).

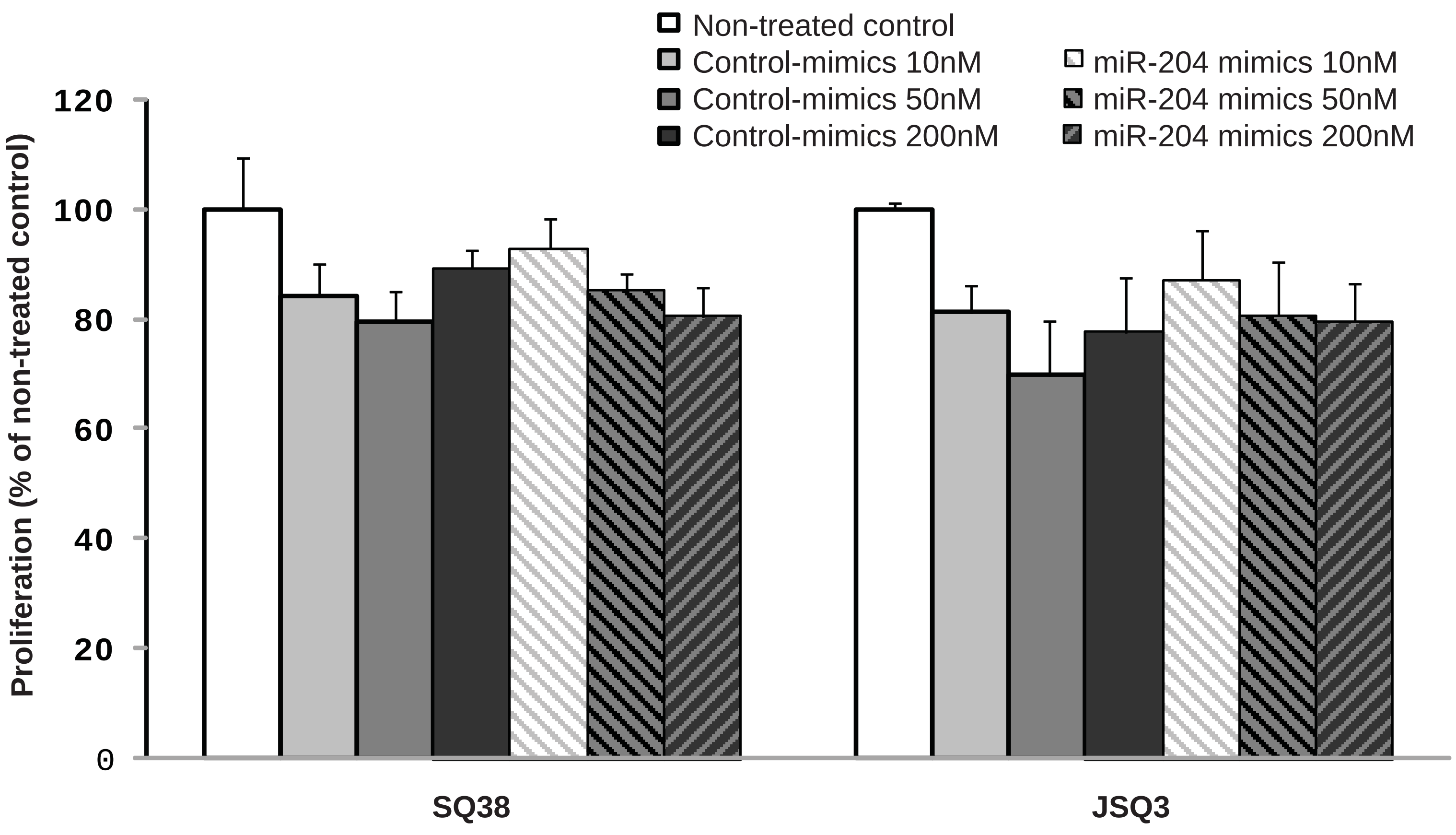

Supporting Figure 6. Restoration of miR-204 function by miR-204 miRIDIAN mimics had no significant effect on SQ38 and JSQ3 HNSCC cell proliferation. HNSCC cells were transfected with different concentrations of either a control or a miR-204 specific miRIDIAN mimics as described in **Methods**. Cell viability and growth was determined by MTT assay at 72h post-transfection. Error bar represents standard error of triplicate repeats of each experiment point.These observations represent one of the two experiments performed (Related to **Figs. 4A-C** of the manuscript).

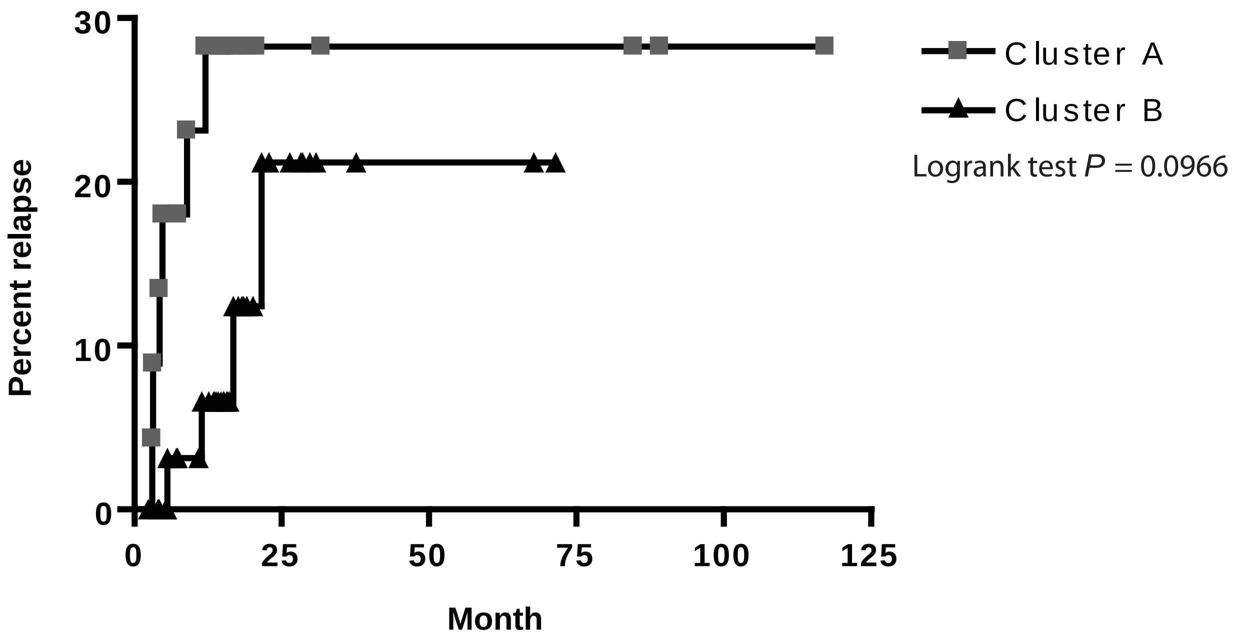

Supporting Figure 7. Kaplan-Meier of the relapse time for patients in Cluster A and B classified by 19 mir-204 targets. However, due to the small number of miR-204 targets (19 genes), the heterogeneous head and neck locations of tumor samples, and the small sample size, the level of statistical significance that we observed ($P = 0.0966$) is lower compared with that using the full 582 gene set ($P = 0.02$) reported by Chung. et al. (Related to **Fig. 5** of the manuscript).

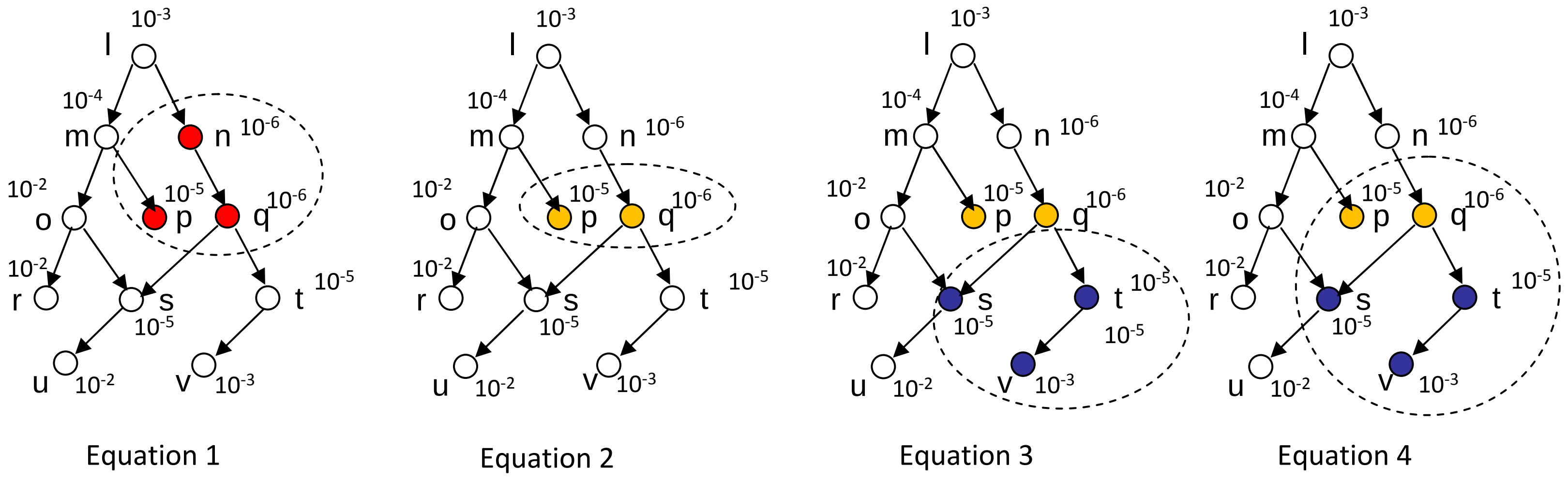

Supporting Figure 8. Refinement of enriched GO or SNOMED terms (related to **Figures 2B-C, 3C, Tables S3** and **S7, Protocol S2). Equations 1, 2** and **3** describe the refinement algorithm over the statistically significant results in an enrichment study. **Equation 1** searches the nodes with the lowest adjusted *P*-value, V_{RM}={v_n, v_p, v_q} (red nodes). **Equation 2** excludes parent nodes that have the same adjusted *P*-value as their child and retains the child nodes in V_{RM}, V_{RRM}={v_p, v_q} (yellow nodes). **Equation 3** identifies the subsumed significant associations of V_{RRM}, V_{SDRRM}={v_s, v_t, v_v} (blue nodes). **Equation 4** is $V_{included}$={v_p, v_q, v_s, v_t, v_v}, the union of V_{RRM} and V_{SDRRM} as the final subset of retained significant results.

***Legend**:* Entities of an ontology (GO terms or SNOMED concepts) are illustrated as circular "nodes" with an associated adjusted *P*-value from the gene enrichment study (e.g. 10^{-3}) and a symbol (e.g. letter "k"). Hierarchical relationships between nodes are illustrated as an arrow pointing from a parent node towards its child node. Nodes identified by an equation are highlighted with color and located inside a dotted ellipse.
