# Supplementary material for: Network Modeling Identifies Molecular Functions Targeted by miR-204 to Suppress Head and Neck Tumor Metastasis
Source: PLoS Comput Biol. 2010 Apr 1;6(4):e1000730. doi: 10.1371/journal.pcbi.1000730 (PMC2848541; doi:10.1371/journal.pcbi.1000730)
Supplement: Text S2 — Supplementary Tables 1–14 (0.19 MB PDF) [file pcbi.1000730.s002.pdf]

Supporting Table 1. Generation of miRNOME by merging five microRNA target databases (related to Figure 1A). Five microRNA gene target databases were downloaded and parsed to set up the comprehensive human microRNA target genes (miRNOME). MiRNOME contains 534 human microRNAs and 17,343 predicted putative microRNA gene targets allowing predictions of more microRNAs targets than any of the individual microRNA target dataset.

Database	Version	Downloaded file	Downloaded date
TargetScan	3.1	Conserved_Family_Conserved_Targets_Info.txt	July-17-2007
PicTar	hg17<br>server version 4.0.24	picTarMiRNA4Way.txt	July-13-2007
miRBase	4<br>based on ensembl 40 and microRNA regisgry release 9.0	v4.txt.homo_sapiens	July-17-2007
miRanda	Old version	by_gene140.xls	July-13-2007
TarBase	TarBase_V4.0	miRNA_Human_Table.txt	July-13-2007

Supporting Table 2. 34 putative tumor supressor microRNAs reproducibly predicted by IMRE in two HNSCC microarrays (related to Figure 1A). Note: the asterisk, "*", is part of the microRNA name and represents a passenger strand form of the microRNA (less dominant form)

microRNA (alphabetic order)	FDR	
	GSE6631	GSE2379
miR-29	8.00E-03	0.003
miR-29a	1.00E-03	0.001
miR-29b	1.00E-03	0.002
miR-29c	1.00E-03	0.004
miR-30e-3p	9.00E-03	0.045
miR-92b	1.00E-03	0.002
miR-98	7.00E-03	0.001
miR-132	1.00E-03	0.018
miR-126/126-3p	2.00E-03	0.026
miR-139	1.00E-03	0.001
miR-181d	1.00E-03	0.031
miR-182*	1.00E-03	0.008
miR-18b	9.00E-03	0.017
miR-193a	3.00E-03	0.017
miR-193b	3.50E-02	0.036
miR-196	1.00E-03	0.022
**miR-204**	**4.00E-03**	**0.029**
miR-211	1.20E-02	0.046
miR-330	2.00E-03	0.028
miR-361	1.00E-03	0.003
miR-376a	2.00E-03	0.002
miR-376b	1.00E-03	0.004
miR-486	1.00E-03	0.023
miR-489	1.00E-03	0.008
miR-518c	1.00E-02	0.008
miR-568	1.00E-03	0.001
miR-577	9.00E-03	0.013
miR-586	8.00E-03	0.002
miR-595	1.00E-03	0.001
miR-660	6.00E-03	0.017
miR-let-7/98	5.00E-03	0.001
miR-let-7b	2.00E-03	0.001
miR-let-7g	2.00E-03	0.001
miR-let-7i	1.00E-03	0.001

Supporting Table 3. **Statistical analysis of microRNA enrichment in the cancer gene subset of OMIM** (related to **Figure 1A,** approach described in **Methods** and in **Protocols S1-S2,** evaluated against a review of literature **Table S4**). To further reduce the number of microRNAs to the most promising candidates amenable for biological characterization, we conducted a statistical enrichment analysis in the OMIM human disease gene database (Online Mendelian Inheritance in Man) containing 610 biologically validated cancer genes among which 586 (96%) are predicted targets of 527 microRNAs in miRNOME. The validity of OMIM-based genetic analysis of cancer microRNA enrichment was confirmed by a review of literature showing that 35 out of 46 “OMIM-predicted cancer microRNAs” were indeed linked to cancer (**Table S4**), significantly more than expected by chance ($P = 1.6x10^{-9}$, one side cumulative hypergeometric test, total microRNAs in miRNOME=534, total reviewed microRNAs=183). The resulting *P*-values were adjusted to correct for multiple comparisons (**Methods**) and false positive P-values derived from the hierarchical classifications were contro

**microRNAs**	**Unadjusted *P-value***	**Adjusted *P-value***	**SNOMED CT concept (see Protocol S1)**
miR-106b	6.29E-08	2.75E-05	Malignant neoplasm, primary (morphologic abnormality)
	1.55E-07	6.77E-05	Malignant neoplasm of primary, secondary, or uncertain origin (morphologic abnormality)
miR-128	2.15E-05	1.29E-02	Primary malignant neoplasm (disorder)
	7.32E-05	4.34E-02	Neoplasm by body site (disorder)
miR-134	2.75E-08	1.20E-05	Synovial sarcoma (morphologic abnormality)
	2.80E-08	1.70E-05	Synovial sarcoma (disorder)
	1.91E-06	8.34E-04	Malignant neoplasm, primary (morphologic abnormality)
	2.88E-06	1.26E-03	Sarcoma, no ICD-O subtype (morphologic abnormality)
	3.85E-06	1.68E-03	Malignant neoplasm of primary, secondary, or uncertain origin (morphologic abnormality)
	3.69E-05	1.60E-02	Soft tissue tumor AND/OR sarcoma (morphologic abnormality)
	3.69E-05	1.60E-02	Sarcoma -category (morphologic abnormality)
miR-17_5p	5.10E-05	2.20E-02	Malignant neoplasm of primary, secondary, or uncertain origin (morphologic abnormality)
	5.15E-05	2.23E-02	Malignant neoplasm, primary (morphologic abnormality)
miR-17-5p/20/93.mr/106/519.d	3.42E-05	1.48E-02	Malignant neoplasm, primary (morphologic abnormality)
	5.70E-05	2.46E-02	Malignant neoplasm of primary, secondary, or uncertain origin (morphologic abnormality)
miR-181	1.32E-05	5.75E-03	Hematopoietic neoplasm (morphologic abnormality)
	4.56E-05	1.97E-02	Malignant hematopoietic neoplasm (morphologic abnormality)
miR-181a	3.64E-05	2.18E-02	Neoplasm of musculoskeletal system (disorder)
miR-181b	4.80E-08	2.91E-05	Neoplasm of musculoskeletal system (disorder)
	7.20E-07	3.15E-04	Synovial sarcoma (morphologic abnormality)
	1.16E-06	7.03E-04	Synovial sarcoma (disorder)
	3.33E-05	2.00E-02	Primary malignant neoplasm of soft tissues (disorder)
	4.79E-05	2.86E-02	Malignant tumor of stomach (disorder)
miR-181c	9.27E-07	4.05E-04	Synovial sarcoma (morphologic abnormality)
	1.20E-06	7.27E-04	Synovial sarcoma (disorder)
miR-181d	2.08E-05	9.05E-03	Synovial sarcoma (morphologic abnormality)
	1.90E-05	1.14E-02	Neoplasm of musculoskeletal system (disorder)
	2.62E-05	1.58E-02	Synovial sarcoma (disorder)
miR-183	4.34E-05	2.60E-02	Primary malignant neoplasm of soft tissues (disorder)
	7.50E-05	3.22E-02	Synovial sarcoma (morphologic abnormality)

microRNAs	Unadjusted *P-value*	Adjusted *P-value*	SNOMED CT concept (see Protocol S1)
	7.07E-05	4.19E-02	Synovial sarcoma (disorder)
miR-185	2.45E-06	1.07E-03	Synovial sarcoma (morphologic abnormality)
	2.48E-06	1.50E-03	Synovial sarcoma (disorder)
miR-187	1.26E-07	7.64E-05	Neoplasm of musculoskeletal system (disorder)
	3.06E-06	1.34E-03	Synovial sarcoma (morphologic abnormality)
	4.59E-06	2.78E-03	Synovial sarcoma (disorder)
miR-18a*	5.17E-07	2.26E-04	Synovial sarcoma (morphologic abnormality)
	3.91E-07	2.37E-04	Synovial sarcoma (disorder)
	1.16E-05	7.00E-03	Malignant neoplasm of soft tissue (disorder)
	1.87E-05	1.13E-02	Primary malignant neoplasm of soft tissues (disorder)
miR-193	4.76E-07	2.08E-04	Acute myeloid leukemia, no ICD-O subtype (morphologic abnormality)
	2.06E-06	9.00E-04	Myeloid leukemia, no ICD-O subtype (morphologic abnormality)
	5.22E-05	3.11E-02	Primary malignant neoplasm (disorder)
	9.63E-05	4.12E-02	Acute leukemia, morphology, including blast cell OR undifferentiated leukemia (morphologic abnormality)
miR-197	1.06E-09	4.63E-07	Synovial sarcoma (morphologic abnormality)
	1.70E-09	1.03E-06	Synovial sarcoma (disorder)
miR-20	7.13E-05	3.07E-02	Malignant neoplasm, primary (morphologic abnormality)
	1.03E-04	4.40E-02	Malignant neoplasm of primary, secondary, or uncertain origin (morphologic abnormality)
miR-204	1.07E-05	4.67E-03	Malignant lymphoma - category (morphologic abnormality)
	1.15E-05	5.01E-03	Malignant lymphoma, no ICD-O subtype (morphologic abnormality)
	4.78E-05	2.07E-02	Lymphoid neoplasm (morphologic abnormality)
miR-20a	4.89E-07	2.14E-04	Malignant neoplasm, primary (morphologic abnormality)
	1.04E-06	4.54E-04	Malignant neoplasm of primary, secondary, or uncertain origin (morphologic abnormality)
miR-211	4.09E-07	1.79E-04	Malignant lymphoma, no ICD-O subtype (morphologic abnormality)
	2.60E-06	1.14E-03	Malignant lymphoma - category (morphologic abnormality)
	4.19E-05	1.81E-02	Lymphoid neoplasm (morphologic abnormality)
miR-221/222	4.49E-05	1.94E-02	Leukemia, no ICD-O subtype (morphologic abnormality)
	4.84E-05	2.09E-02	Leukemia - category (morphologic abnormality)
	8.32E-05	4.92E-02	Leukemia, disease (disorder)
miR-27	8.85E-05	3.79E-02	Astrocytoma, no ICD-O subtype (morphologic abnormality)
miR-30-5p	2.10E-05	9.14E-03	Malignant hematopoietic neoplasm (morphologic abnormality)
	3.60E-05	1.56E-02	Myeloid leukemia, no ICD-O subtype (morphologic abnormality)
miR-320	2.42E-05	1.05E-02	Malignant neoplasm of primary, secondary, or uncertain origin (morphologic abnormality)
miR-381	8.19E-05	3.52E-02	Myeloid leukemia, no ICD-O subtype (morphologic abnormality)
miR-409-5p	1.30E-08	5.68E-06	Synovial sarcoma (morphologic abnormality)
	1.62E-08	9.82E-06	Synovial sarcoma (disorder)
	3.51E-07	2.13E-04	Primary malignant neoplasm of soft tissues (disorder)
	8.34E-06	5.04E-03	Malignant neoplasm of soft tissue (disorder)
	5.07E-05	2.19E-02	Sarcoma, no ICD-O subtype (morphologic abnormality)
miR-431	6.51E-05	2.80E-02	Synovial sarcoma (morphologic abnormality)
	5.87E-05	3.49E-02	Synovial sarcoma (disorder)
miR-452	1.99E-08	8.70E-06	Synovial sarcoma (morphologic abnormality)
	2.96E-08	1.79E-05	Synovial sarcoma (disorder)
	8.01E-05	3.44E-02	Sarcoma, no ICD-O subtype (morphologic abnormality)

microRNAs	Unadjusted *P-value*	Adjusted *P-value*	SNOMED CT concept (see Protocol S1)
miR-500	9.07E-13	5.50E-10	Synovial sarcoma (disorder)
	1.49E-12	6.51E-10	Synovial sarcoma (morphologic abnormality)
	5.98E-08	2.61E-05	Sarcoma, no ICD-O subtype (morphologic abnormality)
	1.12E-06	4.89E-04	Soft tissue tumor AND/OR sarcoma (morphologic abnormality)
	1.12E-06	4.89E-04	Sarcoma -category (morphologic abnormality)
miR-505	4.69E-08	2.05E-05	Synovial sarcoma (morphologic abnormality)
	3.52E-08	2.13E-05	Synovial sarcoma (disorder)
	1.03E-05	6.22E-03	Primary malignant neoplasm of soft tissues (disorder)
	1.63E-05	9.83E-03	Neoplasm of musculoskeletal system (disorder)
miR-574	2.18E-07	1.32E-04	Synovial sarcoma (disorder)
	3.33E-07	1.46E-04	Synovial sarcoma (morphologic abnormality)
miR-579	1.34E-09	5.86E-07	Synovial sarcoma (morphologic abnormality)
	2.18E-09	1.32E-06	Synovial sarcoma (disorder)
	4.12E-06	1.80E-03	Sarcoma, no ICD-O subtype (morphologic abnormality)
	2.35E-05	1.02E-02	Soft tissue tumor AND/OR sarcoma (morphologic abnormality)
	2.35E-05	1.02E-02	Sarcoma -category (morphologic abnormality)
miR-586	3.07E-06	1.86E-03	Synovial sarcoma (disorder)
	5.04E-06	2.20E-03	Synovial sarcoma (morphologic abnormality)
miR-595	3.07E-06	1.86E-03	Synovial sarcoma (disorder)
	4.68E-06	2.04E-03	Synovial sarcoma (morphologic abnormality)
	1.11E-05	6.70E-03	Primary malignant neoplasm of soft tissues (disorder)
	1.16E-05	7.00E-03	Malignant neoplasm of soft tissue (disorder)
miR-652	2.02E-06	8.82E-04	Synovial sarcoma (morphologic abnormality)
	3.88E-06	2.35E-03	Synovial sarcoma (disorder)
miR-93	1.70E-05	7.40E-03	Malignant neoplasm of primary, secondary, or uncertain origin (morphologic abnormality)
	1.87E-05	8.14E-03	Malignant neoplasm, primary (morphologic abnormality)
miR-93.hd/291-3p/294/295/302/372/373/520	1.01E-06	4.41E-04	Malignant hematopoietic neoplasm (morphologic abnormality)
	1.98E-05	1.19E-02	Leukemia, disease (disorder)
	3.58E-05	1.55E-02	Leukemia, no ICD-O subtype (morphologic abnormality)
	3.92E-05	1.70E-02	Leukemia - category (morphologic abnormality)
miR-let-7	8.83E-06	5.34E-03	Malignant tumor of urinary bladder (disorder)
	9.50E-05	4.07E-02	Acute leukemia, morphology, including blast cell OR undifferentiated leukemia (morphologic abnormality)
	1.14E-04	4.86E-02	Central nervous system tumor morphology (morphologic abnormality)
miR-let-7a	1.33E-06	5.81E-04	Synovial sarcoma (morphologic abnormality)
	1.16E-06	7.03E-04	Synovial sarcoma (disorder)
miR-let-7b	1.38E-05	6.01E-03	Synovial sarcoma (morphologic abnormality)
	1.44E-05	8.69E-03	Synovial sarcoma (disorder)
miR-let-7c	8.60E-07	3.76E-04	Synovial sarcoma (morphologic abnormality)
	8.65E-07	5.24E-04	Synovial sarcoma (disorder)
	2.45E-05	1.47E-02	Primary malignant neoplasm of soft tissues (disorder)
	5.16E-05	2.23E-02	Sarcoma, no ICD-O subtype (morphologic abnormality)
	4.09E-05	2.45E-02	Neoplasm of musculoskeletal system (disorder)
miR-let-7d	1.10E-05	4.80E-03	Synovial sarcoma (morphologic abnormality)
	1.40E-05	8.45E-03	Synovial sarcoma (disorder)
	4.59E-07	2.01E-04	Synovial sarcoma (morphologic abnormality)
	5.46E-07	3.31E-04	Synovial sarcoma (disorder)

**microRNAs**	**Unadjusted *P-value***	**Adjusted *P-value***	**SNOMED CT concept (see Protocol S1)**
miR-let-7e	6.32E-06	3.82E-03	Malignant neoplasm of soft tissue (disorder)
	1.51E-05	9.11E-03	Primary malignant neoplasm of soft tissues (disorder)
	2.18E-05	9.48E-03	Sarcoma, no ICD-O subtype (morphologic abnormality)
	2.53E-05	1.52E-02	Neoplasm of musculoskeletal system (disorder)
miR-let-7f	8.39E-07	3.67E-04	Synovial sarcoma (morphologic abnormality)
	6.38E-07	3.87E-04	Synovial sarcoma (disorder)
	7.70E-06	4.66E-03	Malignant neoplasm of soft tissue (disorder)
	1.78E-05	1.07E-02	Primary malignant neoplasm of soft tissues (disorder)
	2.98E-05	1.79E-02	Neoplasm of musculoskeletal system (disorder)
miR-let-7g	1.37E-06	5.99E-04	Synovial sarcoma (morphologic abnormality)
	1.00E-06	6.06E-04	Synovial sarcoma (disorder)
miR-let-7i	1.37E-06	5.99E-04	Synovial sarcoma (morphologic abnormality)
	1.04E-06	6.30E-04	Synovial sarcoma (disorder)
	5.63E-06	3.41E-03	Neoplasm of musculoskeletal system (disorder)
	2.97E-05	1.78E-02	Primary malignant neoplasm of soft tissues (disorder)

Supporting Table 4. Literature review of microRNAs down-regulated in cancer (related to Figure S3). A combinatorial text-based search using two keywords "miRNA" and "cancer" was conducted in PubMed (on Feb. 28 2008). 28 microRNA papers reporting microRNAs down-regulated in cancer compared to a normal tissue were selected and reviewed in details. 133 microRNAs were identified. 123 microRNAs among 133 microRNAs were identified by microRNA array analysis in various types of cancer and only the expression status of 30 out of 133 microRNAs was confirmed by PCR or northern. Note: the asterisk, "*", is part of the microRNA name and represents a passenger strand form of the microRNA (less dominant form)

microRNAs (alphabetic order)	Method of detection			Normal control		Cancer Type	Reference
	Charaterized by		Identified by microRNA Array				
	PCR	Northern		Tissue	Paired		
let-7	√			Adjacent normal tissue	Paired	Colon	Biol Pharm Bull (2006) 29, p903
		√		Normal adjacent sample		Lung	Cell (2005) 120, p635
let-7a	√		√	Non-cancerous lung tissue	Paired	Lung	Cancer Cell (2006) 9, p189
			√	Normal ovarian tissue	Unpaired	Ovarian	Cancer Res (2007) 18, p8699
			√	Normal		Breast	PNAS (2006) 103, p2257 [let-7a-2]
			√	Normal		Prostate	PNAS (2006) 103, p2257 [let-7a-2]
let-7b	√	√	√	Normal bone marrow control		Leukemia	PNAS (2007) 11, p19971
let-7c			√	Normal ovarian tissue	Unpaired	Ovarian	Cancer Res (2007) 18, p8699
			√	Normal adjacent thyroid tissue		Thyroid anaplastic carcinoma	Oncogene (2007) 26, p7590
let-7d			√	Normal ovarian tissue	Unpaired	Ovarian	Cancer Res (2007) 18, p8699
let-7f			√	Normal adjacent tissue or controlateral - normal thyroid lobe		Papillary thyroid carcinoma	Endocr Relat Cancer (2006) 13, p497
let-7g			√	Normal tissue		CNS	Cancer Res (2007) 67, p2456
let-7i			√	Normal tissue		CNS	Cancer Res (2007) 67, p2456
miR-1			√	Adjacent non-tumorous tissue	Paired	Colon	JAMA (2008) 299, p425
miR-100			√	Normal ovarian tissue	Unpaired	Ovarian	Cancer Res (2007) 18, p8699
			√	Normal		Breast	PNAS (2006) 103, p2257
miR-101			√	Corresponding non-cancerous lung tissue	Paired	Lung	Cancer Cell (2006) 9, p189
			√	Normal ovarian tissue	Unpaired	Ovarian	Cancer Res (2007) 18, p8699
miR-103			√	Normal tissue		CNS	Cancer Res (2007) 67, p2456
miR-105			√	Normal ovarian tissue	Unpaired	Ovarian	Cancer Res (2007) 18, p8699
miR-107			√	Normal tissue		CNS	Cancer Res (2007) 67, p2456
miR-10a			√	Normal tissue		Hematologic	Cancer Res (2007) 67, p2456

microRNAs (alphabetic order)	Method of detection			Normal control		Cancer Type	Reference
	Charaterized by						
	PCR	Northern	Identified by microRNA Array	Tissue	Paired		
miR-10b			√	Normal breast tissue	Unpaired	Breast	Cancer Res (2005) 65, p7065
			√	Normal		Breast	PNAS (2006) 103, p2257
miR-123			√	Normal breast tissue	Unpaired	Breast	Cancer Res (2005) 65, p7065
miR-124a			√	Corresponding non-cancerous lung tissue	Paired	Lung	Cancer Cell (2006) 9, p189
			√	Normal ovarian tissue	Unpaired	Ovarian	Cancer Res (2007) 18, p8699
			√	Normal tissue		CNS	Cancer Res (2007) 67, p2456
miR-124b			√	Normal tissue		CNS	Cancer Res (2007) 67, p2456
miR-125			√	Normal ovarian tissue	Unpaired	Ovarian	Cancer Res (2007) 18, p8699
miR-125a		√	√	Adjacent normal tissue	Paired	Hepatocellular carcinoma	Oncogene (2006) 25, p2537
			√	Corresponding non-cancerous lung tissue	Paired	Lung	Cancer Cell (2006) 9, p189
			√	Normal adjacent thyroid tissue		Thyroid anaplastic carcinoma	Oncogene (2007) 26, p7590
miR-125b		√	√	Normal breast tissue	Unpaired	Breast	Cancer Res (2005) 65, p7065
	√	√	√	Normal adjacent thyroid tissue		Thyroid anaplastic carcinomas	Oncogene (2007) 26, p7590
			√	Normal		Breast	PNAS (2006) 103, p2257 [miR-125b-1]
			√	Normal		Breast	PNAS (2006) 103, p2257 [miR-125b-2]
miR-126			√	Corresponding non-cancerous lung tissue	Paired	Lung	Cancer Cell (2006) 9, p189
			√	Normal ovarian tissue	Unpaired	Ovarian	Cancer Res (2007) 18, p8699
miR-126*			√	Normal		Lung	PNAS (2006) 103, p2257
miR-127		√		Adjacent normal tissue	Paired	Bladder	Cancer Cell (2006) 9, p435
		√		Adjacent normal tissue	Paired	Prostate	Cancer Cell (2006) 9, p435
			√	Normal ovarian tissue	Unpaired	Ovarian	Cancer Res (2007) 18, p8699
miR-128a		√	√	Normal brain	purchased	Glioblastoma	Biochem Biophys Res Commu (2005) 334, p1351
			√	Normal tissue		CNS	Cancer Res (2007) 67, p2456
			√	Normal		Prostate	PNAS (2006) 103, p2257
miR-128b			√	Normal tissue		CNS	Cancer Res (2007) 67, p2456
miR-130a			√	Normal		Breast	PNAS (2006) 103, p2257
miR-133a			√	Normal ovarian tissue	Unpaired	Ovarian	Cancer Res (2007) 18, p8699
miR-133b		√	√	Adjacent normal colorectal tissue	Paired	Colon	Mol Cancer (2006) 19, p29
miR-134			√	Normal tissue		CNS	Cancer Res (2007) 67, p2456
miR-135			√	Normal adjacent thyroid tissue		Thyroid anaplastic carcinoma	Oncogene (2007) 26, p7590
miR-135a			√	Normal tissue		CNS	Cancer Res (2007) 67, p2456

microRNAs (alphabetic order)	Method of detection: Charaterized by PCR	Method of detection: Charaterized by Northern	Identified by microRNA Array	Normal control: Tissue	Normal control: Paired	Cancer Type	Reference
miR-136			√	Normal		Stomach	PNAS (2006) 103, p2257
miR-137			√	Normal tissue		CNS	Cancer Res (2007) 67, p2456
miR-138			√	Normal tissue		CNS	Cancer Res (2007) 67, p2456
			√	Normal adjacent thyroid tissue		Thyroid anaplastic carcinoma	Oncogene (2007) 26, p7590
			√	Adjacent normal thyroid tissue	Paired	Papillary thyroid carcinoma	PNAS (2005) 102, p19075 [miR-138-1]
			√	Normal		Stomach	PNAS (2006) 103, p2257 [miR-138-2]
miR-140	√		√	Normal ovarian tissue		Ovarian	Cancer Res (2007) 18, p8699
			√	Corresponding non-cancerous lung tissue	Paired	Lung	Cancer Cell (2006) 9, p189
			√	Normal		Breast	PNAS (2006) 103, p2257
miR-140-as			√	Normal breast tissue	Unpaired	Breast	Cancer Res (2005) 65, p7065
			√	Normal adjacent tissue or contralateral normal thyroid lobe		Papillary thyroid carcinoma	Endocr Relat Cancer (2006) 13, p497
miR-142-as			√	Normal adjacent tissue or contralateral normal thyroid lobe		Papillary thyroid carcinoma	Endocr Relat Cancer (2006) 13, p497
miR-143		√	√	Normal tissue		Cervix	Cancer Res (2007) 67, p6031
		√		Normal mucosa	Paired	Colon	Mol Cancer Res (2003) 1, p882
			√	Corresponding non-cancerous lung tissue	Paired	Lung	Cancer Cell (2006) 9, p189
miR-143b			√	Normal ovarian tissue	Unpaired	Ovarian	Cancer Res (2007) 18, p8699
miR-144			√	Normal tissue from posterior myometrium		Uterine leiomyoma	Genes Chr Cancer (2007) 46, p336
miR-145		√	√	Normal breast tissue	Unpaired	Breast	Cancer Res (2005) 65, p7065
		√	√	Adjacent normal colorectal tissue	Paired	Colon	Mol Cancer (2006) 19, p29
		√		Normal mucosae	Paired	Colon	Mol Cancer Res (2003) 1, p882
			√	Corresponding non-cancerous lung tissue	Paired	Lung	Cancer Cell (2006) 9, p189
			√	Normal adjacent thyroid tissue		Thyroid anaplastic carcinoma	Oncogene (2007) 26, p7590
			√	Normal		Breast	PNAS (2006) 103, p2257
miR-147			√	Normal ovarian tissue	Unpaired	Ovarian	Cancer Res (2007) 18, p8699
miR-148			√	Normal adjacent tissue or contralateral normal thyroid lobe		Papillary thyroid carcinoma	Endocr Relat Cancer (2006) 13, p497

microRNAs (alphabetic order)	Method of detection: Charaterized by PCR	Method of detection: Charaterized by Northern	Method of detection: Identified by microRNA Array	Normal control: Tissue	Normal control: Paired	Cancer Type	Reference
miR-148a			√	Normal tissue		Colon	Cancer Res (2007) 67, p2456
miR-148b			√	Adjacent non-tumorous tissue	Paired	Colon	JAMA (2008) 299, p425
miR-149			√	Normal		Prostate	PNAS (2006) 103, p2257
miR-151			√	Normal adjacent tissue or contralateral normal thyroid lobe		Papillary thyroid carcinoma	Endocr Relat Cancer (2006) 13, p497
			√	Normal adjacent thyroid tissue		Thyroid anaplastic carcinoma	Oncogene (2007) 26, p7590
miR-152			√	Normal		Pancreas	PNAS (2006) 103, p2257
miR-154*			√	Normal tissue		CNS	Cancer Res (2007) 67, p2456
miR-155		√	√	Normal pancreas	Paired	Pancreas	J Clin Oncol (2006) 24, p4677
			√	Normal		Pancreas	PNAS (2006) 103, p2257
miR-15a		√		Buccal mucosa	Paired	Leukemia	PNAS (2002) 99, p15524
	√		√	Normal blood	Unpaired	Leukemia	PNAS (2004) 101, p11755
		√	√	Normal blood	Unpaired	Leukemia	PNAS (2005) 102, p13944
		√		Normal pituitary	Unpaired	Pituitary adenomas	J Cell Physiol (2005) 204, p280
			√	Normal tissue		Colon	Cancer Res (2007) 67, p2456
miR-15b			√	Normal adjacent tissue or contralateral normal thyroid lobe		Papillary thyroid carcinoma	Endocr Relat Cancer (2006) 13, p497
miR-16		√		Buccal mucosa	Paired	Leukemia	PNAS (2002) 99, p15524
	√	√		Normal blood	Unpaired	Leukemia	PNAS (2004) 101, p11755
		√	√	Normal blood	Unpaired	Leukemia	PNAS (2005) 102, p13944
		√		Normal pituitary	Unpaired	Pituitary adenomas	J Cell Physiol (2005) 204, p280
			√	Normal		Breast	PNAS (2006) 103, p2257 [miR-16-2]
miR-17-3p			√	Normal tissue		CNS	Cancer Res (2007) 67, p2456
miR-181a		√	√	Normal brain	purchased	Glioblastoma	Biochem Biophys Res Commu (2005) 334, p1351
			√	Normal tissue		CNS	Cancer Res (2007) 67, p2456
miR-181b		√	√	Normal brain	purchased	Glioblastoma	Biochem Biophys Res Commu (2005) 334, p1351
	√		√	Bon marrow		Leukemia	Oncogene (2007) 14, p4148
miR-181c		√	√	Normal brain	purchased	Glioblastoma	Biochem Biophys Res Commu (2005) 334, p1351
			√	Corresponding non-cancerous lung tissue	Paired	Lung	Cancer Cell (2006) 9, p189
			√	Normal tissue		CNS	Cancer Res (2007) 67, p2456
miR-181d			√	Normal tissue		CNS	Cancer Res (2007) 67, p2456

microRNAs (alphabetic order)	Method of detection: Charaterized by PCR	Method of detection: Charaterized by Northern	Method of detection: Identified by microRNA Array	Normal control: Tissue	Normal control: Paired	Cancer Type	Reference
miR-191			√	Normal tissue		CNS	Cancer Res (2007) 67, p2456
miR-192			√	Normal tissue		CNS	Cancer Res (2007) 67, p2456
			√	Adjacent non-tumorous tissue	Paired	Colon	JAMA (2008) 299, p425
miR-194			√	Normal breast tissue	Unpaired	Breast	Cancer Res (2005) 65, p7065
			√	Normal tissue		CNS	Cancer Res (2007) 67, p2456
miR-195		√	√	Adjacent normal tissue	Paired	Hepatocellular carcinoma	Oncogene (2006) 25, p2537
miR-196b			√	Normal tissue		Hematologic	Cancer Res (2007) 67, p2456
miR-197			√	Normal tissue		CNS	Cancer Res (2007) 67, p2456
			√	Normal tissue from posterior myometrium		Uterine leiomyoma	Genes Chr Cancer (2007) 46, p336
miR-198			√	Corresponding non-cancerous lung tissue	Paired	Lung	Cancer Cell (2006) 9, p189
miR-199a		√	√	Adjacent Normal tissue	Paired	Hepatocellular carcinoma	Oncogene (2006) 25, p2537
			√	Corresponding non-cancerous lung tissue	Paired	Lung	Cancer Cell (2006) 9, p189
			√	Normal adjacent tissue or contralateral Normal thyroid lobe		Papillary thyroid carcinoma	Endocr Relat Cancer (2006) 13, p497
miR-199b			√	Normal ovarian tissue	Unpaired	Ovarian	Cancer Res (2007) 18, p8699
miR-200a		√	√	Adjacent Normal tissue	Paired	Hepatocellular carcinoma	Oncogene (2006) 25, p2537
miR-203			√	Normal adjacent thyroid tissue		Thyroid anaplastic carcinoma	Oncogene (2007) 26, p7590
**miR-204**			**√**	**Normal breast tissue**	**Unpaired**	**Breast**	**Cancer Res (2005) 65, p7065**
miR-205			√	Normal		Breast	PNAS (2006) 103, p2257
miR-206		√	√	Normal blood	Unpaired	Leukemia	PNAS (2004) 101, p11755
miR-21	√		√	Normal blood	Unpaired	Leukemia	PNAS (2004) 101, p11755
miR-211			√	Normal ovarian tissue	Unpaired	Ovarian	Cancer Res (2007) 18, p8699
miR-212	√	√	√	Normal tissue from posterior myometrium		Uterine leiomyoma	Genes Chromosomes Cancer (2007) 46, p336
			√	Normal tissue		CNS	Cancer Res (2007) 67, p2456
			√	Normal		Stomach	PNAS (2006) 103, p2257
miR-213			√	Normal tissue		CNS	Cancer Res (2007) 67, p2456
miR-214			√	Normal ovarian tissue	Unpaired	Ovarian	Cancer Res (2007) 18, p8699
			√	Normal tissue		Colon	Cancer Res (2007) 67, p2456
miR-215			√	Adjacent non-tumorous tissue	Paired	Colon	JAMA (2008) 299, p425
miR-216			√	Corresponding non-cancerous lung tissue	Paired	Lung	Cancer Cell (2006) 9, p189

microRNAs (alphabetic order)	Method of detection: Charaterized by PCR	Method of detection: Charaterized by Northern	Method of detection: Identified by microRNA Array	Normal control: Tissue	Normal control: Paired	Cancer Type	Reference
miR-218			√	Corresponding non-cancerous lung tissue	Paired	Lung	Cancer Cell (2006) 9, p189
miR-218			√	Normal		Prostate	PNAS (2006) 103, p2257 [miR-218-2]
miR-218			√	Normal		Stomach	PNAS (2006) 103, p2257 [miR-218-2]
miR-219			√	Corresponding non-cancerous lung tissue	Paired	Lung	Cancer Cell (2006) 9, p189
miR-219			√	Normal tissue		CNS	Cancer Res (2007) 67, p2456
miR-219			√	Adjacent normal thyroid tissue	Paired	Papillary thyroid carcinoma	PNAS (2005) 102, p19075 [miR-219-1]
miR-220			√	Corresponding non-cancerous lung tissue	Paired	Lung	Cancer Cell (2006) 9, p189
miR-223		√	√	Normal blood	Unpaired	Leukemia	PNAS (2004) 101, p11755
miR-223	√	√	√	Normal bone marrow control		Leukemia	PNAS (2007) 11, p19971
miR-224			√	Corresponding non-cancerous lung tissue	Paired	Lung	Cancer Cell (2006) 9, p189
miR-224			√	Normal ovarian tissue	Unpaired	Ovarian	Cancer Res (2007) 18, p8699
miR-224			√	Normal adjacent thyroid tissue		Thyroid anaplastic carcinoma	Oncogene (2007) 26, p7590
miR-224			√	Normal		Breast	PNAS (2006) 103, p2257
miR-24			√	Normal		Prostate	PNAS (2006) 103, p2257 [miR-24-1]
miR-25			√	Normal adjacent thyroid tissue		Thyroid anaplastic carcinoma	Oncogene (2007) 26, p7590
miR-26a		√		Normal blood	Unpaired	Leukemia	PNAS (2004) 101, p11755
miR-26a	√		√	Normal adjacent thyroid tissue		Thyroid anaplastic carcinomas	Oncogene (2007) 26, p7590
miR-26a			√	Corresponding non-cancerous lung tissue	Paired	Lung	Cancer Cell (2006) 9, p189
miR-26a			√	Adjacent normal thyroid tissue	Paired	Papillary thyroid carcinoma	PNAS (2005) 102, p19075 [miR-26a-1]
miR-26b			√	Normal tissue		CNS	Cancer Res (2007) 67, p2456
miR-27b			√	Corresponding non-cancerous lung tissue	Paired	Lung	Cancer Cell (2006) 9, p189
miR-28			√	Normal tissue		Hematologic	Cancer Res (2007) 67, p2456
miR-29b	√		√	Normal tissue from posterior myometrium		Uterine leiomyoma	Genes Chromosomes Cancer (2007) 46, p336
miR-29b			√	corresponding non-cancerous lung tissue	Paired	Lung	Cancer Cell (2006) 9, p189

microRNAs (alphabetic order)	Method of detection: Charaterized by PCR	Method of detection: Charaterized by Northern	Method of detection: Identified by microRNA Array	Normal control: Tissue	Normal control: Paired	Cancer Type	Reference
			√	Normal adjacent thyroid tissue		Thyroid anaplastic carcinoma	Oncogene (2007) 26, p7590
miR-301			√	Adjacent non-tumorous tissue	Paired	Colon	JAMA (2008) 299, p425
miR-30a-3p			√	Adjacent non-tumorous tissue	Paired	Colon	JAMA (2008) 299, p425
miR-30a-5p	√		√	Normal adjacent thyroid tissue		Thyroid anaplastic carcinomas	Oncogene (2007) 26, p7590
			√	Corresponding non-cancerous lung tissue	Paired	Lung	Cancer Cell (2006) 9, p189
			√	Normal		Lung	PNAS (2006) 103, p2257
miR-30b			√	Normal tissue		CNS	Cancer Res (2007) 67, p2456
miR-30c			√	Normal tissue		CNS	Cancer Res (2007) 67, p2456
			√	Normal		Breast	PNAS (2006) 103, p2257
miR-30d	√		√	Normal blood	Unpaired	Leukemia	PNAS (2004) 101, p11755
	√		√	Normal adjacent thyroid tissue		Thyroid anaplastic carcinomas	Oncogene (2007) 26, p7590
			√	Normal tissue		CNS	Cancer Res (2007) 67, p2456
			√	Normal		Lung	PNAS (2006) 103, p2257
miR-32		√	√	Normal tissue from posterior myometrium		Uterine leiomyoma	Genes Chromosomes Cancer (2007) 46, p336
			√	Corresponding non-cancerous lung tissue	Paired	Lung	Cancer Cell (2006) 9, p189
			√	Normal adjacent thyroid tissue		Thyroid anaplastic carcinoma	Oncogene (2007) 26, p7590
miR-320			√	Nonmalignant cholangiocytes		Cholangiocarcinoma	Gastroenterology (2006) 130, p2113
			√	Nonmalignant cholangiocytes		Cholangiocarcinoma	Gastroenterology (2006) 130, p2113
miR-321			√	Normal tissue		CNS	Cancer Res (2007) 67, p2456
miR-323			√	Normal tissue		CNS	Cancer Res (2007) 67, p2456
miR-324-3p			√	Normal tissue		CNS	Cancer Res (2007) 67, p2456
miR-324-5p			√	Normal tissue		CNS	Cancer Res (2007) 67, p2456
			√	Adjacent non-tumorous tissue	Paired	Colon	JAMA (2008) 299, p425
miR-326			√	Normal pancreas	Paired	Pancreas	J Clin Oncol (2006) 24, p4677
miR-328			√	Normal tissue		CNS	Cancer Res (2007) 67, p2456
miR-33			√	Corresponding non-cancerous lung tissue	Paired	Lung	Cancer Cell (2006) 9, p189

microRNAs (alphabetic order)	Method of detection: Charaterized by PCR	Method of detection: Charaterized by Northern	Method of detection: Identified by microRNA Array	Normal control: Tissue	Normal control: Paired	Cancer Type	Reference
miR-330			√	Normal tissue		CNS	Cancer Res (2007) 67, p2456
miR-331			√	Normal tissue		CNS	Cancer Res (2007) 67, p2456
			√	Adjacent non-tumorous tissue	Paired	Colon	JAMA (2008) 299, p425
miR-338			√	Normal tissue		CNS	Cancer Res (2007) 67, p2456
miR-339			√	Normal pancreas	Paired	Pancreas	J Clin Oncol (2006) 24, p4677
miR-33b			√	Normal		Stomach	PNAS (2006) 103, p2257
miR-342			√	Adjacent non-tumorous tissue	Paired	Colon	JAMA (2008) 299, p425
miR-345			√	Normal tissue		CNS	Cancer Res (2007) 67, p2456
			√	Adjacent Normal thyroid tissue	Paired	Papillary thyroid carcinoma	PNAS (2005) 102, p19075
miR-346			√	Normal tissue		CNS	Cancer Res (2007) 67, p2456
miR-34a	√			Normal adrenal tissue		Neuroblastoma	Oncogene (2007) 26, p5017
			√	Normal tissue		CNS	Cancer Res (2007) 67, p2456
miR-34b			√	Adjacent non-tumorous tissue	Paired	Colon	JAMA (2008) 299, p425
miR-34c			√	Adjacent non-tumorous tissue	Paired	Colon	JAMA (2008) 299, p425
miR-361			√	Normal tissue		CNS	Cancer Res (2007) 67, p2456
miR-370			√	Normal tissue		CNS	Cancer Res (2007) 67, p2456
miR-374			√	Normal ovarian tissue	Unpaired	Ovarian	Cancer Res (2007) 18, p8699
miR-378			√	Normal tissue		Colon	Cancer Res (2007) 67, p2456
miR-424			√	Normal tissue		Colon	Cancer Res (2007) 67, p2456
miR-425			√	Normal tissue		CNS	Cancer Res (2007) 67, p2456
miR-9			√	Corresponding non-cancerous lung tissue	Paired	Lung	Cancer Cell (2006) 9, p189
			√	Normal ovarian tissue	Unpaired	Ovarian	Cancer Res (2007) 18, p8699
			√	Adjacent normal thyroid tissue	Paired	Papillary thyroid carcinoma	PNAS (2005) 102, p19075 [miR-9-3]
			√	Normal		Colon	PNAS (2006) 103, p2257 [miR-9-3]
miR-92			√	Normal adjacent thyroid tissue		Thyroid anaplastic carcinoma	Oncogene (2007) 26, p7590
miR-95			√	Corresponding non-cancerous lung tissue	Paired	Lung	Cancer Cell (2006) 9, p189
miR-96			√	Normal		Stomach	PNAS (2006) 103, p2257
miR-98			√	Normal tissue		CNS	Cancer Res (2007) 67, p2456
miR-99			√	Normal ovarian tissue	Unpaired	Ovarian	Cancer Res (2007) 18, p8699

microRNAs (alphabetic order)	Method of detection			Normal control		Cancer Type	Reference
	Charaterized by		Identified by microRNA Array	Tissue	Paired		
	PCR	Northern					
miR-99a			√	Normal adjacent thyroid tissue		Thyroid anaplastic carcinoma	Oncogene (2007) 26, p7590
mR-422a			√	Normal tissue		Colon	Cancer Res (2007) 67, p2456

Supporting Table 5. Summary of clinical features of 10 low passages HNSCC cell lines established from human HNSCC tumors with persistent disease or local failure after treatment (related to Figure 1C, Figure 2 and Figure 4). (Ref: Brachman, DG et al., Cancer Res. 52: 4832-4836, 1992).

LF: local failure and all died of the disease; NED: no evidence of disease; Outcome: interval since treatment initiation.

**Tumor**	**Primary site**	**TNM stage**	**Outcome**
SCC25	Floor of mounth	T2N1M0	persistent
SCC35	Pyriform sinus	T4N0M0	LF, 25mo
SCC58	Base of tongue	T4N2bM0	NED
SCC61	Oral tongue	T4N2bM0	persistent
Nu61	Derived from SCC61	N/A	N/A
SCC135	Hard palate	T1N0M0	LF, 6mo
SCC151	Oral tongue	T3N0M0	LF, 3mo
SQ20B	Larynx	T2N0M0	LF, 4mo
SQ38	Pyriform sinus	T2N0M0	LF, 7mo
JSQ3	Nasal vestibule	T3N0M0	LF, 4mo

Supporting Table 6. Microarray analysis of mRNA expression status of 34 predicted miR-204 targets in GSE6631 (related to Figure 2A). Intersection of 1,088 putative gene targeted by miR-204 in the miRNOME with 382 differentially upregulated mRNA transcripts in GSE6631 microarray yielded 34 predicted miR-204 targets associated with malignant transformation in HNSCC. False discovery rate (FDR) was derived from significant analysis of microarrays (SAM) (**Methods**).

Gene symbol	Gene name	Fold Change	FDR *
ARPC1B	actin related protein 2/3 complex, subunit 1B	2.89	0
ATP2B1	ATPase, Ca++ transporting, plasma membrane 1	2.54	0
AURKB	aurora kinase B	4.29	0
BIRC2	baculoviral IAP repeat-containing 2	2.91	0.060
BMP1	bone morphogenetic protein 1	3.03	0
CBX1	chromobox homolog 1	2.13	0
CDC25B	cell division cycle 25 homolog B	3.76	0
CDH11	cadherin 11, type 2, OB-cadherin	6.81	0
CHST2	carbohydrate sulfotransferase 2	4.21	0
CTSC	cathepsin C	5.30	0
EFNB1	ephrin-B1	2.54	0
ERF	Ets2 repressor factor	2.15	0
FAP	fibroblast activation protein, alpha	24.84	0
FBN2	fibrillin 2	5.05	0
GPX7	glutathione peroxidase 7	2.24	0.060
HMGA2	high mobility group AT-hook 2	2.18	0
HOXB7	homeobox B7	2.56	0
IGF2BP3	insulin-like growth factor 2 mRNA binding protein 3	6.92	0
INHBA	inhibin, beta A	2.24	0
ITGB4	integrin, beta 4	4.57	0
KLHDC3	kelch domain containing 3	3.50	0
LTBP1	latent transforming growth factor beta binding protein 1	5.71	0
MMP3	matrix metallopeptidase 3	13.46	0
MMP9	matrix metallopeptidase 9	16.04	0
MRPS12	mitochondrial ribosomal protein S12	2.20	0
MYO10	myosin X	3.64	0
NCDN	neurochondrin	2.40	0.060
NUAK1	NUAK family, SNF1-like kinase, 1	2.85	0
PRAME	preferentially expressed antigen in melanoma	2.47	0.060
PTDSS1	phosphatidylserine synthase 1	2.04	0
SHC1	SHC transforming protein 1	2.36	0
SLC12A4	solute carrier family 12, member 4	2.58	0
SOX4	SRY-box 4	3.38	0
SPARC	secreted protein, acidic, cysteine-rich	6.77	0

Supporting Table 7. Functional analysis of predicted HNSCC miR-204 targets by Gene Ontology (Methods, Figures 2B-C, 3C, and S8). To provide insights into biological functions and processes potentially regulated by miR-204 in HNSCC, we conducted statistical analyses based on the functional assignments of genes in Gene Ontology (GO) to infer deregulated functions associated with altered miR-204 expression in the miRNOME as well as in the HNSCC. Statistical enrichment of specific “Molecular functions” and “Biological processes” found in GO was calculated within the 1088 total putative targets in the miRNOME (**Methods**). Except KLHDC3 and NCDN, other 32 of the 34 predicted miR-204 targets are functionally related. We chose to further characterize the expression of the miR-204 targets in the 5 GO functional modules (highlighted in yellow) in HNSCC tumors (**Figure 2B**) and cell lines (**Figures 2C**). * Double enrichment (**Methods**)

	GO ID	GO function description	Double enrichment*			HNSCC miR-204 targets in GO modules
			Genes (#)	*P*	Adjusted *P*	
Biological processes	GO:0006508	┐ proteolysis	5	5.89E-04	3.88E-03	BMP1,CTSC,FAP,MMP3,MMP9
	GO:0006412	┐ translation	3	2.96E-03	9.18E-03	IGF2BP3,INHBA,MRPS12
	GO:0022402	┐ cell cycle process	5	1.05E-02	2.18E-02	AURKB,CDC25B,ERF,INHBA,SHC1
	GO:0022403	├ cell cycle phase	4	7.98E-04	4.20E-03	AURKB,CDC25B,SHC1, INHBA
	GO:0000074	└ regulation of progression through cell cycle	4	1.40E-02	2.60E-02	CDC25B,ERF,INHBA,SHC1
	GO:0032501	┐ multicellular organismal process	13	6.47E-03	1.59E-02	SPARC,MMP3,ITGB4,INHBA,HOXB7,HMGA2,FBN2,EFNB1,CHST2,CDH11,BMP1,BIRC2
	GO:0032502	┐ developmental process │	14	2.90E-03	9.18E-03	BIRC2,BMP1,CDH11,CHST2,EFNB1,FBN2,HMGA2,HOXB7,IGF2BP3,INHBA,ITGB4,MMP9,SHC1,SPARC
	GO:0009653	├┬ anatomical structure morphogenesis	6	6.58E-03	1.61E-02	BIRC2,EFNB1,FBN2,IGF2BP3,INHBA,SHC1
	GO:0000902	│└─ cell morphogenesis	3	1.12E-02	2.25E-02	EFNB1,SHC1,INHBA
	GO:0048856	└┬ anatomical structure development	10	2.94E-03	9.18E-03	BIRC2,FBN2,IGF2BP3
	GO:0009888	├ tissue development	3	1.28E-03	5.78E-03	BMP1,CDH11,SPARC
	GO:0048513	├ organ development	6	1.21E-02	2.36E-02	BMP1,CDH11,SPARC,MMP9,INHBA,BIRC2
	GO:0048731	└ system development	7	2.39E-02	3.97E-02	BIRC2,BMP1,CDH11,EFNB1,INHBA,MMP9,SPARC
	GO:0048522	┐ positive regulation of cellular process	6	3.52E-03	1.06E-02	CDC25B,EFNB1,INHBA,BIRC2,MMP9,SHC1
	GO:0008284	┐ positive regulation of cell proliferation	3	5.76E-04	3.87E-03	CDC25B,EFNB1,SHC1
	GO:0022610	┐ biological adhesion	5	1.95E-03	8.36E-03	ARPC1B,ITGB4,EFNB1,BMP1,CDH11
	GO:0007155	└─ cell adhesion	5	1.95E-03	8.36E-03	ARPC1B,ITGB4,EFNB1,BMP1,CDH11
	GO:0032989	┐ cellular structure morphogenesis	3	1.12E-02	2.25E-02	EFNB1,INHBA,SHC1

	GO ID	GO function description	Double enrichment*			HNSCC miR-204 targets in GO modules
			Genes (#)	***P***	Adjusted ***P***	
	GO:0007167	┐enzyme linked receptor protein signaling pathway	3	2.96E-03	9.18E-03	SPARC,SHC1,INHBA
	GO:0008283	┐cell proliferation	4	8.04E-03	1.81E-02	SHC1,CDC25B,EFNB1,ERF
	GO:0002376	┐immune system process	4	1.65E-02	2.99E-02	INHBA,MMP9,CTSC,EFNB1
Molecular functions	GO:0003824	┐catalytic activity	13	2.77E-02	4.35E-02	ATP2B1,AURKB,BMP1,CDC25B,CHST2,CTSC,FAP,GPX7,LTBP1,MMP3,MMP9,NUAK1,PTDSS1
Molecular functions	GO:0004674	├ protein serine/threonine kinase activity	3	1.56E-02	2.83E-02	AURKB,NUAK1,LTBP1
Molecular functions	GO:0016787	└┬ hydrolase activity	7	2.18E-02	3.65E-02	ATP2B1,BMP1,CDC25B,CTSC,FAP,MMP3,MMP9
Molecular functions	GO:0008233	└┬ peptidase activity	5	1.43E-04	6.22E-04	BMP1,CTSC,FAP,MMP3,MMP9
Molecular functions	GO:0004175	└ endopeptidase activity	5	1.42E-05	6.79E-05	BMP1,CTSC,FAP,MMP3,MMP9
Molecular functions	GO:0043167	┐ion binding	13	1.67E-02	2.99E-02	ATP2B1,AURKB,BIRC2,BMP1,CDH11,CTSC,FBN2,LTBP1,MMP3,MMP9,NUAK1,SLC12A4,SPAR
Molecular functions	GO:0005515	┐protein binding	23	5.97E-04	2.44E-03	ARPC1B,ATP2B1,AURKB,BIRC2,BMP1,CBX1,CDC25B,CDH11,EFNB1,ERF,FAP,HMGA2,HOXB7,INHBA,ITGB4,LTBP1,MMP9,MRPS12,MYO10,NUAK1,PRAME,SHC1,SPARC
Molecular functions	GO:0005102	└ receptor binding	4	6.54E-03	1.51E-02	BMP1,EFNB1,INHBA,SHC1
Molecular functions	GO:0005198	┐structural molecule activity	3	1.12E-02	2.25E-02	ARPC1B,FBN2,MRPS12

Supporting Table 8. Description of the 3'-UTR binding sites of 34 predicted mRNA targets of miR-204 in the upregulated gene expression set of GSE6631. We examined the sequence alignment between the miR-204 and the 3'-UTR region of these gene targets, and ranked them according to (i) the count of databases in which they were predicted, (ii) the number of binding sites present, and (iii) the prediction score and the free energy score. While all 34 genes contain at least one miR-204 binding site, the top 10 ranked genes have multiple sites. These observations describe how miR-204 may directly regulate the expression of all 34 predicted targets.

<table>
<tr><th>Gene Symbol</th><th>PicTar (kcal/mol)</th><th>TargetScan Score</th><th>miRanda (kcal/mol)</th><th>miRBase energy</th><th>3' UTR alignment</th><th>The Num. of binding sites</th></tr>
<tr><td>MYO10</td><td>-16.5</td><td>-0.3</td><td>-18.3</td><td>-</td><td>5'-UGAGCAUUAAAAAGCAAAGGGAA-3'</td><td>3</td></tr>
<tr><td></td><td></td><td></td><td></td><td></td><td>|||| |||||||</td><td>(PicTar)</td></tr>
<tr><td></td><td></td><td></td><td></td><td></td><td>3'- UCCGUAUCCUACUGUUUCCCUU-5'</td><td></td></tr>
<tr><td>ATP2B1</td><td>-31.8</td><td>-0.33</td><td>-</td><td>-</td><td>5'---CAUAGGGAAUAGAGAAAAGGGAA-3'</td><td>6</td></tr>
<tr><td></td><td></td><td></td><td></td><td></td><td>|||||| ||||||||</td><td>(PicTar)</td></tr>
<tr><td></td><td></td><td></td><td></td><td></td><td>3'UCCGUAUCCUACUG----UUUCCCUU-5'</td><td></td></tr>
<tr><td>HMGA2</td><td>-17.4</td><td>-0.39</td><td>-</td><td>-</td><td>5'---AUGUUCAUUUUUCAA-AAAGGGAA-3'</td><td>5</td></tr>
<tr><td></td><td></td><td></td><td></td><td></td><td>||| | ||||||||</td><td>(PicTar)</td></tr>
<tr><td></td><td></td><td></td><td></td><td></td><td>3'-----UCCGUAUCCUACUGUUUCCCUU-5'</td><td></td></tr>
<tr><td>SOX4</td><td>-20.5</td><td>-0.66</td><td>-</td><td>-</td><td>5'-GUGCAAUUACAGCAAAAAGGGAU-3'</td><td>5</td></tr>
<tr><td></td><td></td><td></td><td></td><td></td><td>||| | ||||||||</td><td>(PicTar)</td></tr>
<tr><td></td><td></td><td></td><td></td><td></td><td>3'-UCCGUAUCCUACUG-UUUCCCUU-5'</td><td></td></tr>
<tr><td>CDH11</td><td>-21.4</td><td>-0.15</td><td>-</td><td>-</td><td>5'-GCUGAACUGACAAUGAAGGGAAA-3'</td><td>4</td></tr>
<tr><td></td><td></td><td></td><td></td><td></td><td>| || | ||||||||</td><td>(PicTar)</td></tr>
<tr><td></td><td></td><td></td><td></td><td></td><td>3'-UCCGUAUCCUACUGUUUCCCUU-5'</td><td></td></tr>
<tr><td>SHC1</td><td>-20.4</td><td>-0.37</td><td>-</td><td>-</td><td>5'-GGUCUCACUUAUACCAAAGGGAA-3'</td><td>4</td></tr>
<tr><td></td><td></td><td></td><td></td><td></td><td>|| | |||||||||</td><td>(PicTar)</td></tr>
<tr><td></td><td></td><td></td><td></td><td></td><td>3'-_UCCGUAUCCUACUGUUUCCCUU-5'</td><td></td></tr>
<tr><td>FBN2</td><td>-17.4</td><td>-0.61</td><td>-</td><td>-</td><td>5'-AAACUAUGUAAACAAAAAGGGAA…3'</td><td>3</td></tr>
<tr><td></td><td></td><td></td><td></td><td></td><td>|| || || ||||||||</td><td>(PicTar)</td></tr>
<tr><td></td><td></td><td></td><td></td><td></td><td>3'UCCGUAUCCUACUG--UUUCCCUU-5'</td><td></td></tr>
<tr><td>IGF2BP3</td><td>-15.7</td><td>-0.03</td><td>-</td><td>-</td><td>5'-UAACAUGAGUAACCU-AAGGGAAG-3'</td><td>3</td></tr>
<tr><td></td><td></td><td></td><td></td><td></td><td>|||||||||</td><td>(PicTar)</td></tr>
<tr><td></td><td></td><td></td><td></td><td></td><td>3'-- UCCGUAUCCUACUGUUUCCCUU-5'</td><td></td></tr>
<tr><td>SPARC</td><td>-22.7</td><td>-</td><td>-</td><td>-25.5</td><td>5'-taaatTAGGAAGACAAAGGGAA-3'</td><td>2</td></tr>
<tr><td></td><td></td><td></td><td></td><td></td><td>||||| |||||||||||</td><td>(PicTar)</td></tr>
<tr><td></td><td></td><td></td><td></td><td></td><td>3'-uccguAUCCUACUGUUUCCCUU-5'</td><td></td></tr>
<tr><td>CDC25B</td><td>-15.4</td><td>-0.05</td><td>-</td><td>-</td><td>5'-GUUUUGUUGCCCCAGAAAGGGAU-3'</td><td>2</td></tr>
<tr><td></td><td></td><td></td><td></td><td></td><td>| | | ||||||||</td><td>(PicTar)</td></tr>
<tr><td></td><td></td><td></td><td></td><td></td><td>3'-_UCCGUAUCCUACUGUUUCCCUU-5'</td><td></td></tr>
<tr><td>NUAK1</td><td>-</td><td>-</td><td>-11.36</td><td>-</td><td>5'-tGGC-TACCACGGGAAAGGGAA-3'</td><td>4</td></tr>
<tr><td></td><td></td><td></td><td></td><td></td><td>||| || | |: ||||||||</td><td>(miRnada)</td></tr>
<tr><td></td><td></td><td></td><td></td><td></td><td>3'-uCCGUAUCCUACUGUUUCCCUU-5'</td><td></td></tr>
<tr><td>NCDN</td><td>-28.9</td><td>-</td><td>-</td><td>-</td><td>5'-accCAGGGGCGGGCAGAGAGGGAA-3'</td><td>2</td></tr>
<tr><td></td><td></td><td></td><td></td><td></td><td>|| :|| |:| |:||||||</td><td>(PicTar)</td></tr>
</table>

<table>
<tr><th>Gene Symbol</th><th>PicTar (kcal/mol)</th><th>TargetScan Score</th><th>miRanda (kcal/mol)</th><th>miRBase energy</th><th>3' UTR alignment</th><th>The Num. of binding sites</th></tr>
<tr><td></td><td></td><td></td><td></td><td></td><td>3'-uccGUAUCCUACUG--UUUCCCUU-5'</td><td></td></tr>
<tr><td>PRAME</td><td>-</td><td>-</td><td>-</td><td>-18.83</td><td>5'-tcagtgAGGAAAAAAAGGGGAA-3'</td><td>2</td></tr>
<tr><td></td><td></td><td></td><td></td><td></td><td> |||| | ||:|||||</td><td>(miRBase)</td></tr>
<tr><td></td><td></td><td></td><td></td><td></td><td>3'-uccguaUCCUACUGUUUCCCUU-5'</td><td></td></tr>
<tr><td>CBX1</td><td>-</td><td>-</td><td>-17.6</td><td>-</td><td>5'-tccCAGTGGAGGGGAAAGGGGA-3'</td><td>2</td></tr>
<tr><td></td><td></td><td></td><td></td><td></td><td>   ...||..|||.|:.||||||:|</td><td>(miRnada)</td></tr>
<tr><td></td><td></td><td></td><td></td><td></td><td>3'-uccGUAUCCUACUGUUUCCCUU-5'</td><td></td></tr>
<tr><td>ERF</td><td>-</td><td>-</td><td>-</td><td>-30.05</td><td>5'-GGGCAAAGTGGTGGTAGGGGGAG-3'</td><td>1</td></tr>
<tr><td></td><td></td><td></td><td></td><td></td><td>   :|||| || |:||::|::||||:</td><td>(miRBase)</td></tr>
<tr><td></td><td></td><td></td><td></td><td></td><td>3'-UCCGUAUC-CUACUGUUUCCCUU-5'</td><td></td></tr>
<tr><td>INHBA</td><td>-</td><td>-</td><td>-</td><td>-25.64</td><td>5'-AGGAATTGGGAGGGAAAGGGAG-3'</td><td>1</td></tr>
<tr><td></td><td></td><td></td><td></td><td></td><td> ||| || ||: |: |||||||:</td><td>(miRBase)</td></tr>
<tr><td></td><td></td><td></td><td></td><td></td><td>3'-UCCGUAUCCUACUGUUUCCCUU-5'</td><td></td></tr>
<tr><td>CHST2</td><td>-</td><td>-</td><td>-</td><td>-24.7</td><td>5'-GGGTGGGGGTGGAGAAAGGGGA-3'</td><td>1</td></tr>
<tr><td></td><td></td><td></td><td></td><td></td><td>   :||:: :|| || ||||||:|</td><td>(miRBase)</td></tr>
<tr><td></td><td></td><td></td><td></td><td></td><td>3'-UCCGUAUCCUACUGUUUCCCUU-5'</td><td></td></tr>
<tr><td>AURKB</td><td>-</td><td>-</td><td>-</td><td>-22.16</td><td>5'-ATGTATAGGGGAAAGAAGGGA-3'</td><td>1</td></tr>
<tr><td></td><td></td><td></td><td></td><td></td><td> | |:|||||: | :||||||</td><td>(miRBase)</td></tr>
<tr><td></td><td></td><td></td><td></td><td></td><td>3'-UCCGUAUCCUACUGUUUCCCUu-5'</td><td></td></tr>
<tr><td>HOXB7</td><td>-</td><td>-</td><td>-</td><td>-22.09</td><td>5'-AGGAAGAGACATGAGAAAGGGAG-3'</td><td>1</td></tr>
<tr><td></td><td></td><td></td><td></td><td></td><td> ||| | || |||| |||||||:</td><td>(miRBase)</td></tr>
<tr><td></td><td></td><td></td><td></td><td></td><td>3'-UCCGUAUC-CUACUGUUUCCCUU-5'</td><td></td></tr>
<tr><td>SLC12A4</td><td>-</td><td>-</td><td>-</td><td>-22.03</td><td>5'-cacCATCAGA-GACGAAGGGAA-3'</td><td>1</td></tr>
<tr><td></td><td></td><td></td><td></td><td></td><td> ||| || |||:|||||||</td><td>(miRBase)</td></tr>
<tr><td></td><td></td><td></td><td></td><td></td><td>3'-UCCGUAUCCUACUGUUUCCCUU-5'</td><td></td></tr>
<tr><td>MRPS12</td><td>-</td><td>-</td><td>-</td><td>-20.42</td><td>5'-AGTCCTGGGGGTGCAAAGGGtg-3'</td><td>1</td></tr>
<tr><td></td><td></td><td></td><td></td><td></td><td> || | |:||: :|||||||</td><td>(miRBase)</td></tr>
<tr><td></td><td></td><td></td><td></td><td></td><td>3'-UCCGUAUCCUACUGUUUCCCuu-5'</td><td></td></tr>
<tr><td>PTDSS1</td><td>-</td><td>-</td><td>-</td><td>-19.6</td><td>5'-CAGCGCGGTGTGTTGAAGGGAA-3'</td><td>1</td></tr>
<tr><td></td><td></td><td></td><td></td><td></td><td> ||: :| :|| ::|||||||</td><td>(miRBase)</td></tr>
<tr><td></td><td></td><td></td><td></td><td></td><td>3'-UCCGUAUCCUACUGUUUCCCUU-5'</td><td></td></tr>
<tr><td>ITGB4</td><td>-</td><td>-</td><td>-</td><td>-19.48</td><td>5'-TCTCCTGGGAGGCATGAAGGGGG-3'</td><td>1</td></tr>
<tr><td></td><td></td><td></td><td></td><td></td><td> | |:||| | |::|||||::</td><td>(miRBase)</td></tr>
<tr><td></td><td></td><td></td><td></td><td></td><td>3'-UCCGUAUCCUAC-UGUUUCCCUU-5'</td><td></td></tr>
<tr><td>KLHDC3</td><td>-</td><td>-</td><td>-</td><td>-19.47</td><td>5'-TCTGCTGGGAAGG-GAAGGGAA-3'</td><td>1</td></tr>
<tr><td></td><td></td><td></td><td></td><td></td><td> |:||| |: :|||||||</td><td>(miRBase)</td></tr>
<tr><td></td><td></td><td></td><td></td><td></td><td>3'-UCCGUAUCCUACUGUUUCCCUU-5'</td><td></td></tr>
<tr><td>GPX7</td><td>-</td><td>-</td><td>-</td><td>-18.53</td><td>5'-tcaaATGGTGCTTCAAAGGGAG-3'</td><td>1</td></tr>
<tr><td></td><td></td><td></td><td></td><td></td><td> ||:| : ||||||||:</td><td>(miRBase)</td></tr>
<tr><td></td><td></td><td></td><td></td><td></td><td>3'-uccgUAUCCUACUGUUUCCCUU-5'</td><td></td></tr>
<tr><td>CTSC</td><td>-</td><td>-</td><td>-</td><td>-18.02</td><td>5'-ucCGUAUCCUACUGUUUCCCUU-3'</td><td>1</td></tr>
<tr><td></td><td></td><td></td><td></td><td></td><td> |||| | || ||:|||||</td><td>(miRBase)</td></tr>
<tr><td></td><td></td><td></td><td></td><td></td><td>3'-ctGCATCAGTTGTAAAGGGGAA-5'</td><td></td></tr>
<tr><td>LTBP1</td><td>-</td><td>-</td><td>-16.5</td><td>-</td><td>5'-GU-GUAAAGGA-----AAAGGGAG-3'</td><td>1</td></tr>
<tr><td></td><td></td><td></td><td></td><td></td><td>   : |:| |||| |||||||:</td><td>(miRnada)</td></tr>
</table>

<table>
<tr><th>Gene Symbol</th><th>PicTar (kcal/mol)</th><th>TargetScan Score</th><th>miRanda (kcal/mol)</th><th>miRBase energy</th><th>3' UTR alignment</th><th>The Num. of binding sites</th></tr>
<tr><td></td><td></td><td></td><td></td><td></td><td>3'-UC.CGUAUCCUACUG.UUUCCCUU-5'</td><td></td></tr>
<tr><td>MMP9</td><td>-</td><td>-</td><td>-</td><td>-15.15</td><td>5'-ttctgttctggaggAAAGGGAG-3'</td><td>1</td></tr>
<tr><td></td><td></td><td></td><td></td><td></td><td>|||||||:</td><td>(miRBase)</td></tr>
<tr><td></td><td></td><td></td><td></td><td></td><td>3'-uccguauccuacugUUUCCCUU-5'</td><td></td></tr>
<tr><td>MMP3</td><td>-</td><td>-</td><td>-</td><td>-14.15</td><td>5'-atctttcccAAGAGAAGGGGAA-3'</td><td>1</td></tr>
<tr><td></td><td></td><td></td><td></td><td></td><td>| || ||:|||||</td><td>(miRBase)</td></tr>
<tr><td></td><td></td><td></td><td></td><td></td><td>3'-uccguauccUACUGUUUCCCUU-5'</td><td></td></tr>
<tr><td>ARPC1B</td><td>-</td><td>-</td><td>-</td><td>-14.1</td><td>5'-ggctgctcccTCAAAAAGGGAG-3'</td><td>1</td></tr>
<tr><td></td><td></td><td></td><td></td><td></td><td>| | |||||||:</td><td>(miRBase)</td></tr>
<tr><td></td><td></td><td></td><td></td><td></td><td>3'-uccguauccuACUGUUUCCCUU-5'</td><td></td></tr>
<tr><td>FAP</td><td>-</td><td>-</td><td>-</td><td>-13.32</td><td>5'-CTGTGAAAGAAGAGAAAAGGGAG-3'</td><td>1</td></tr>
<tr><td></td><td></td><td></td><td></td><td></td><td>|:: | || || |||||||:</td><td>(miRBase)</td></tr>
<tr><td></td><td></td><td></td><td></td><td></td><td>3'-UCCGUAUCCUACU-GUUUCCCUU-5'</td><td></td></tr>
<tr><td>BIRC2</td><td>-</td><td>-</td><td>-</td><td>-12.16</td><td>5'-AGCCATCTAAAGTAAAAAGGGAA-3'</td><td>1</td></tr>
<tr><td></td><td></td><td></td><td></td><td></td><td>|| ||| | | | ||||||||</td><td>(miRBase)</td></tr>
<tr><td></td><td></td><td></td><td></td><td></td><td>3'-UCCGUAUCCUAC-UGUUUCCCUU-5'</td><td></td></tr>
<tr><td>BMP1</td><td>-</td><td>-</td><td>-10.6</td><td>-</td><td>5'-tGTTGTGAAGTAAAAGAGGGAc-3'</td><td>1</td></tr>
<tr><td></td><td></td><td></td><td></td><td></td><td>.|.::|:..:|.|.|:|||||.</td><td>(miRnada)</td></tr>
<tr><td></td><td></td><td></td><td></td><td></td><td>3'-uCCGUAUCCUACUGUUUCCCUu-5'</td><td></td></tr>
<tr><td>EFNB1</td><td>-</td><td>-</td><td>-</td><td>-</td><td>5'AGGCAGGGCTGGACACTGATGGACAGCAGGCAGGGAG 3'</td><td>1</td></tr>
<tr><td></td><td></td><td></td><td></td><td></td><td>||||| |:| ||| ||| : |||||:</td><td>(miRBase)</td></tr>
<tr><td></td><td></td><td></td><td></td><td></td><td>3'UCCGU----AUC-----CUA-CUG-----UUUCCCUU 5'</td><td></td></tr>
</table>

Supporting Table 9. Three cross references. This table describes three cross-reference tables, ensemblXref3, kgXref, and a core data from HUGO's HGNC were used, when required, to convert each target gene ID to its corresponding HGNC gene symbol (**Methods**).

Database	Version	Downloaded file	Downloaded date	Usage	microRNA dataset	URL
ensemblXref	hg18	ensemblXref3	July-19-2007	mapping Uniprot entry name to approved gene symbol	miRBase	http://hgdownload.cse.ucsc.edu/goldenPath/hg18/database/
				mapping Uniprot entry name to approved gene symbol	miRanda	
				mapping ensembl Id to Uniprot entry name	miRanda	
"All data export"		All data	July-13-2007	mapping refseq ID to approved gene symbol	picTar	http://www.genenames.org/data/gdlw_index.html
				mapping Uniprot entry name to approved gene symbol	miRanda	
				mapping Uniprot entry name to approved gene symbol	miRBase	
				mapping Uniprot entry name to approved gene symbol	miRanda	
kgXref	hg18	kgXref	July-19-2007	mapping Uniprot entry name to approved gene symbol	miRBase	http://hgdownload.cse.ucsc.edu/goldenPath/hg18/database/
				mapping Uniprot entry name to approved gene symbol	miRanda	
				mapping refseq ID to approved gene symbol	picTar	

Supporting Table 10. Datasets used in the protein-protein interaction network (related to Figure 3). Seven protein interactions network datasets were downloaded and standardized to two-column lists of pair-wise interactions between SwissProt accession IDs, with an additional column providing the source dataset and references to the literature when available.

Database	File name	Filtering	Used original ID to be converted swissProt accession number	Used the cross reference to convert original ID to swissProt accession number	Type of interaction (from d/l files)	Download date	URL
BioGRID	BIOGRID-ORGANISM-2.0.25.tab	Yes	HGNC Unique ID	HGNC	-	March 17, 2007	http://www.thebiogrid.org/downloads.php
Reactome	homo_sapiens.interactions.txt	Yes	swissProt accession number	-	4	October 27, 2006	http://reactome.org/download/index.html
DIP	Hsapi20070107	No	swissProt accession number	-	-	January 7, 2007	http://dip.doe-mbi.ucla.edu/dip/Download.cgi
MINT	Mammalia-mitab	Yes	swissProt accession number	-	9	December 5, 2006	http://mint.bio.uniroma2.it/mint/download.do
HPRD	HPRD_Release_6_10012006	Yes	Entrez Gene identifier	HGNC	-	December 6, 2006	http://www.hprd.org/download
BIND	20060525.nrints	Yes	BIND identifier	binddbxref.1	-	October 10, 2006	http://bond.unleashedinformatics.com
KEGG	Release 4.0 of hsaXXX.xml (XXX = all numbered data)	No	swissProt accession number	genes_uniprot.list	-	October, 2006	http://www.genome.jp/kegg/

Supporting Table 11. Upregulated genes in HNSCC microarrays (GSE6631) analyzed by single protein interaction network (related to **Figure 3**). The unadjusted *P*-value is assigned to each HNSCC protein by measuring the frequency at which the observed count of protein interaction for that HNSCC protein occurred in the empirical distribution of 10,000 permutation re-sampling of the network (Single Protein Network Modeling in **Methods**). 260 of the upregulated genes could be translated to protein interactions in the protein network (PPIN in Methods). Of these, 56 proteins with unadjusted *P*-value <0.05 were prioritized (named proteins in the network presented in **Figure 3**), and their other interactors are detailed in **Table S12** (unnamed grey dots in **Figure 3).**

Up-regulated gene in HNSCC	Corresponding Swissprot entry name in PPIN	Corre-sponding Swissprot accession number in PPIN	Observed number of protein inter-action partners	Un-adjusted prob-ability	Gene name
COL1A1	CO1A1_HUMAN	P02452	11	<0.0001	Collagen alpha-1(I) chain precursor (Alpha-1 type I collagen)
COL4A1	CO4A1_HUMAN	P02462	8	<0.0001	Collagen alpha-1(IV) chain precursor (Arresten)
FN1	FINC_HUMAN	P02751	11	<0.0001	Fibronectin precursor (FN) (Cold-insoluble globulin) (CIG)
COL1A2	CO1A2_HUMAN	P08123	10	<0.0001	Collagen alpha-2(I) chain precursor (Alpha-2 type I collagen)
COL4A2	CO4A2_HUMAN	P08572	8	<0.0001	Collagen alpha-2(IV) chain precursor [Contains: Canstatin]
SPARC	SPRC_HUMAN	P09486	6	<0.0001	SPARC precursor (Secreted protein acidic and rich in cysteine
MMP9	MMP9_HUMAN	P14780	8	<0.0001	Matrix metalloproteinase-9 precursor (EC 3.4.24.35) (MMP-9) (92 kD
BGN	PGS1_HUMAN	P21810	7	<0.0001	Biglycan precursor (Bone/cartilage proteoglycan I) (PG-S1)
BSG	BASI_HUMAN	P35613	4	<0.0001	Basigin precursor (Leukocyte activation antigen M6) (Collagenas
CKS1B	CKS1_HUMAN	P61024	7	<0.0001	Cyclin-dependent kinases regulatory subunit 1 (CKS-1)
COL7A1	CO7A1_HUMAN	Q02388	5	<0.0001	Collagen alpha-1(VII) chain precursor (Long-chain collagen) (L
LAMB3	LAMB3_HUMAN	Q13751	4	<0.0001	Laminin subunit beta-3 precursor (Laminin 5 beta 3) (Laminin B1
LAMC2	LAMC2_HUMAN	Q13753	5	<0.0001	Laminin subunit gamma-2 precursor (Laminin 5 gamma 2 subunit
TGFBI	BGH3_HUMAN	Q15582	6	<0.0001	Transforming growth factor-beta-induced protein ig-h3 precursor (Bet
CDC2	CDC2_HUMAN	P06493	18	0.0001	Cell division control protein 2 homolog (EC 2.7.11.22) (EC 2.7.11.23
GINS1	PSF1_HUMAN	Q14691	5	0.0001	DNA replication complex GINS protein PSF1
APP	A4_HUMAN	P05067	10	0.0002	Amyloid beta A4 protein precursor (APP) (ABPP) (Alzheimer diseas
ITGA5	ITA5_HUMAN	P08648	6	0.0002	Integrin alpha-5 precursor (Fibronectin receptor subunit alpha
SPP1	OSTP_HUMAN	P10451	4	0.0003	Osteopontin precursor (Bone sialoprotein-1) (Secreted phosphoprotei
BMP1	BMP1_HUMAN	P13497	4	0.0003	Bone morphogenetic protein 1 precursor (EC 3.4.24.19) (BMP-1
NID1	NID1_HUMAN	P14543	7	0.0005	Nidogen-1 precursor (Entactin)

Up-regulated gene in HNSCC	Corresponding Swissprot entry name in PPIN	Corre-sponding Swissprot accession number in PPIN	Observed number of protein inter-action partners	Un-adjusted prob-ability	Gene name
PLEC1	PLEC1_HUMAN	Q15149	5	0.0011	Plectin-1 (PLTN) (PCN) (Hemidesmosomal protein 1) (HD1) (Plectin-11)
LAMA3	LAMA3_HUMAN	Q16787	4	0.0011	Laminin subunit alpha-3 precursor (Epiligrin 170 kDa subunit) (E170
ITGAV	ITAV_HUMAN	P06756	6	0.0016	Integrin alpha-V precursor (Vitronectin receptor subunit alpha) (CD5
ITGA3	ITA3_HUMAN	P26006	5	0.0017	Integrin alpha-3 precursor (Galactoprotein B3) (GAPB3) (VLA-3 alph
CDC20	CDC20_HUMAN	Q12834	10	0.0017	Cell division cycle protein 20 homolog (p55CDC)
ITGB4	ITB4_HUMAN	P16144	4	0.0019	Integrin beta-4 precursor (GP150) (CD104 antigen)
CD44	CD44_HUMAN	P16070	6	0.0033	CD44 antigen precursor (Phagocytic glycoprotein I) (PGP-1) (HUTCH-I
UBE2C	UBE2C_HUMAN	O00762	9	0.0036	Ubiquitin-conjugating enzyme E2 C (EC 6.3.2.19) (Ubiquitin-protei
CCNA2	CCNA2_HUMAN	P20248	6	0.0048	Cyclin-A2 (Cyclin-A)
CCNE1	CCNE1_HUMAN	P24864	5	0.0048	G1/S-specific cyclin-E1
MCM3	MCM3_HUMAN	P25205	6	0.0048	DNA replication licensing factor MCM3 (DNA polymerase alph
HIST1H3A	H31_HUMAN	P68431	4	0.005	Histone H3.1 (H3/a) (H3/b) (H3/c) (H3/d) (H3/f) (H3/h) (H3/i) (H3/j
TNC	TENA_HUMAN	P24821	4	0.0065	Tenascin precursor (TN) (Tenascin-C) (TN-C) (Hexabrachion
CDKN3	CDKN3_HUMAN	Q16667	4	0.007	Cyclin-dependent kinase inhibitor 3 (EC 3.1.3.48) (EC 3.1.3.16) (CDK2
LUM	LUM_HUMAN	P51884	3	0.01	Lumican precursor (Keratan sulfate proteoglycan lumican) (KSP
CKS2	CKS2_HUMAN	P33552	3	0.0112	Cyclin-dependent kinases regulatory subunit 2 (CKS-2)
PLAUR	UPAR_HUMAN	Q03405	5	0.0137	Urokinase plasminogen activator surface receptor precursor (uPAR) (U
EGFR	EGFR_HUMAN	P00533	11	0.0146	Epidermal growth factor receptor precursor (EC 2.7.10.1) (Recepto
CCNB2	CCNB2_HUMAN	O95067	3	0.0186	G2/mitotic-specific cyclin-B2
SERPINE2	GDN_HUMAN	P07093	3	0.0188	Glia-derived nexin precursor (GDN) (Protease nexin I) (PN-1) (Proteas
MCM7	MCM7_HUMAN	P33993	6	0.0256	DNA replication licensing factor MCM7 (CDC47 homolog) (P1.1-MCM3)
BUB1B	BUB1B_HUMAN	O60566	7	0.0261	Mitotic checkpoint serine/threonine-protein kinase BUB1 bet
PLAU	UROK_HUMAN	P00749	4	0.03	Urokinase-type plasminogen activator precursor (EC 3.4.21.73) (uPA
AURKA	STK6_HUMAN	O14965	7	0.0305	Serine/threonine-protein kinase 6 (EC 2.7.11.1) (Aurora kinase A
SHC1	SHC1_HUMAN	P29353	11	0.0309	SHC-transforming protein 1 (SH2 domain protein C1) (Src homology
SLC16A3	MOT4_HUMAN	O15427	2	0.0314	Monocarboxylate transporter 4 (MCT 4) (MCT 3) (Solute carrier famil
SLC7A5	LAT1_HUMAN	Q01650	2	0.0329	Large neutral amino acids transporter small subunit 1 (L-type amin
PSMB2	PSB2_HUMAN	P49721	8	0.033	Proteasome subunit beta type 2 (EC 3.4.25.1) (Proteasome component C7
CDC25B	MPIP2_HUMAN	P30305	4	0.0333	M-phase inducer phosphatase 2 (EC 3.1.3.48) (Dual specificit
KRT17	K1C17_HUMAN	Q04695	2	0.0341	Keratin, type I cytoskeletal 17 (Cytokeratin-17) (CK-17) (Keratin-17

Up-regulated gene in HNSCC	Corresponding Swissprot entry name in PPIN	Corre-sponding Swissprot accession number in PPIN	Observed number of protein inter-action partners	Un-adjusted prob-ability	Gene name
PKMYT1	PMYT1_HUMAN	Q99640	3	0.0363	Membrane-associated tyrosine- and threonine-specific cdc2-inhibitor
STAT1	STAT1_HUMAN	P42224	6	0.0425	Signal transducer and activator of transcription 1-alpha/bet
GAA	LYAG_HUMAN	P10253	3	0.0433	Lysosomal alpha-glucosidase precursor (EC 3.2.1.20) (Acid maltase
AURKB	AURKB_HUMAN	Q96GD4	7	0.0445	Serine/threonine-protein kinase 12 (EC 2.7.11.1) (Aurora-B) (Aurora
MCM6	MCM6_HUMAN	Q14566	5	0.0448	DNA replication licensing factor MCM6 (p105MCM)
MFAP2	MFAP2_HUMAN	P55001	2	0.0504	Microfibrillar-associated protein 2 precursor (MFAP-2) (Microfibril
SLC16A1	MOT1_HUMAN	P53985	2	0.0518	Monocarboxylate transporter 1 (MCT 1) (Solute carrier family 16 membe
PTK2	FAK1_HUMAN	Q05397	6	0.0522	Focal adhesion kinase 1 (EC 2.7.10.2) (FADK 1) (pp125FAK) (Protein
CBLB	CBLB_HUMAN	Q13191	3	0.0539	E3 ubiquitin-protein ligase CBL-B (EC 6.3.2.-) (Signal transductio
PSMD2	PSMD2_HUMAN	Q13200	8	0.0541	26S proteasome non-ATPase regulatory subunit 2 (26S proteasom
CUX1	CUTL1_HUMAN	P39880	3	0.0667	Homeobox protein cut-like 1 (CCAAT displacement protein) (CDP)
TGFB1	TGFB1_HUMAN	P01137	5	0.0727	Transforming growth factor beta-1 precursor (TGF-beta-1) [Contains
LAMC1	LAMC1_HUMAN	P11047	2	0.0754	Laminin subunit gamma-1 precursor (Laminin B2 chain)
MCM2	MCM2_HUMAN	P49736	5	0.0755	DNA replication licensing factor MCM2 (Minichromosome maintenanc
MDK	MK_HUMAN	P21741	3	0.0758	Midkine precursor (MK) (Neurite outgrowth-promoting protein
SPAG5	SPAG5_HUMAN	Q96R06	2	0.0818	Sperm-associated antigen 5 (Astrin) (Mitotic spindle-associate
LAMB1	LAMB1_HUMAN	P07942	2	0.0821	Laminin subunit beta-1 precursor (Laminin B1 chain)
SERPINE1	PAI1_HUMAN	P05121	3	0.085	Plasminogen activator inhibitor 1 precursor (PAI-1) (Endothelia
CD151	CD151_HUMAN	P48509	2	0.0922	CD151 antigen (Platelet-endothelial tetraspan antigen 3) (PETA-3
CALD1	CALD1_HUMAN	Q05682	2	0.0975	Caldesmon (CDM)
CCNB1	CCNB1_HUMAN	P14635	5	0.1035	G2/mitotic-specific cyclin-B1
INHBA	INHBA_HUMAN	P08476	3	0.1263	Inhibin beta A chain precursor (Activin beta-A chain) (Erythroi
FSTL3	FSTL3_HUMAN	O95633	2	0.1293	Follistatin-related protein 3 precursor (Follistatin-like 3
CBX1	CBX1_HUMAN	P83916	3	0.1341	Chromobox protein homolog 1 (Heterochromatin protein 1 homolog beta
GMPS	GUAA_HUMAN	P49915	3	0.1395	GMP synthase [glutamine-hydrolyzing] (EC 6.3.5.2) (Glutamin
CENPA	CENPA_HUMAN	P49450	2	0.1429	Histone H3-like centromeric protein A (Centromere protein A) (CENP-A
RB1	RB_HUMAN	P06400	9	0.1443	Retinoblastoma-associated protein (PP110) (P105-RB) (RB)
PTPRZ1	PTPRZ_HUMAN	P23471	2	0.1513	Receptor-type tyrosine-protein phosphatase zeta precurso
APBB2	APBB2_HUMAN	Q92870	2	0.1513	Amyloid beta A4 precursor protein-binding family B member 2 (Fe65-lik
TXNRD1	TRXR1_HUMAN	Q16881	2	0.1523	Thioredoxin reductase 1, cytoplasmic precursor (EC 1.8.1.9) (TR

Up-regulated gene in HNSCC	Corresponding Swissprot entry name in PPIN	Corre-sponding Swissprot accession number in PPIN	Observed number of protein inter-action partners	Un-adjusted prob-ability	Gene name
FAP	SEPR_HUMAN	Q12884	2	0.1559	Seprase (EC 3.4.21.-) (Fibroblast activation protein alpha) (Integra
COL6A1	CO6A1_HUMAN	P12109	2	0.1602	Collagen alpha-1(VI) chain precursor
THBS2	TSP2_HUMAN	P35442	2	0.1751	Thrombospondin-2 precursor
TPX2	TPX2_HUMAN	Q9ULW0	2	0.1754	Targeting protein for Xklp2 (Restricted expression proliferation
THY1	THY1_HUMAN	P04216	2	0.2006	Thy-1 membrane glycoprotein precursor (Thy-1 antigen) (CD90 antigen
PDGFA	PDGFA_HUMAN	P04085	2	0.202	Platelet-derived growth factor A chain precursor (PDGF A-chain
CALR	CALR_HUMAN	P27797	3	0.2081	Calreticulin precursor (CRP55) (Calregulin) (HACBP) (ERp60) (grp60)
FCGR2A	FCG2A_HUMAN	P12318	2	0.2204	Low affinity immunoglobulin gamma Fc region receptor II-a precurso
EXT1	EXT1_HUMAN	Q16394	2	0.2292	Exostosin-1 (EC 2.4.1.224) (EC 2.4.1.225) (Glucuronosyl-N
COL3A1	CO3A1_HUMAN	P02461	2	0.2303	Collagen alpha-1(III) chain precursor
IMPDH1	IMDH1_HUMAN	P20839	3	0.2421	Inosine-5'-monophosphate dehydrogenase 1 (EC 1.1.1.205) (IM
MMP1	MMP1_HUMAN	P03956	2	0.2455	Interstitial collagenase precursor (EC 3.4.24.7) (Matri
IGF1R	IGF1R_HUMAN	P08069	4	0.2674	Insulin-like growth factor 1 receptor precursor (EC 2.7.10.1
AP2B1	AP2B1_HUMAN	P63010	2	0.2685	AP-2 complex subunit beta-1 (Adapter-related protein complex 2 beta-
CBX3	CBX3_HUMAN	Q13185	2	0.2709	Chromobox protein homolog 3 (Heterochromatin protein 1 homolog gamma
IGFBP7	IBP7_HUMAN	Q16270	2	0.2799	Insulin-like growth factor-binding protein 7 precursor (IGFBP-7) (IBP
EXT2	EXT2_HUMAN	Q93063	2	0.2832	Exostosin-2 (EC 2.4.1.224) (EC 2.4.1.225) (Glucuronosyl-N
MMP14	MMP14_HUMAN	P50281	2	0.2883	Matrix metalloproteinase-14 precursor (EC 3.4.24.80) (MMP-14
PDGFRB	PGFRB_HUMAN	P09619	4	0.3125	Beta-type platelet-derived growth factor receptor precurso
SLC3A2	4F2_HUMAN	P08195	2	0.3215	4F2 cell-surface antigen heavy chain (4F2hc) (Lymphocyte activatio
RRM2	RIR2_HUMAN	P31350	3	0.3685	Ribonucleoside-diphosphate reductase M2 subunit (EC 1.17.4.1
ITGA6	ITA6_HUMAN	P23229	2	0.4216	Integrin alpha-6 precursor (VLA-6) (CD49f antigen) [Contains: Integri
MYBL2	MYBB_HUMAN	P10244	2	0.4248	Myb-related protein B (B-Myb)
RFC5	RFC5_HUMAN	P40937	3	0.4676	Replication factor C subunit 5 (Replication factor C 36 kDa subunit
TAF6	TAF6_HUMAN	P49848	3	0.4976	Transcription initiation factor TFIID subunit 6 (Transcriptio
GTF2E1	T2EA_HUMAN	P29083	3	0.5548	Transcription initiation factor IIE subunit alpha (TFIIE-alpha
MSN	MOES_HUMAN	P26038	2	0.5633	Moesin (Membrane-organizing extension spike protein)
IRS1	IRS1_HUMAN	P35568	3	0.6408	Insulin receptor substrate 1 (IRS-1)
AHR	AHR_HUMAN	P35869	2	0.6499	Aryl hydrocarbon receptor precursor (Ah receptor) (AhR)
SNAPC1	SNPC1_HUMAN	Q16533	2	0.6655	snRNA-activating protein complex subunit 1 (SNAPc subunit 1) (snRNA

Up-regulated gene in HNSCC	Corresponding Swissprot entry name in PPIN	Corre-sponding Swissprot accession number in PPIN	Observed number of protein inter-action partners	Un-adjusted prob-ability	Gene name
RFC4	RFC4_HUMAN	P35249	2	0.722	Replication factor C subunit 4 (Replication factor C 37 kDa subunit
GNAI1	GNAI1_HUMAN	P63096	2	0.7404	Guanine nucleotide-binding protein G(i), alpha-1 subunit (Adenylat
HPRT1	HPRT_HUMAN	P00492	3	0.753	Hypoxanthine-guanine phosphoribosyltransferase (EC 2.4.2.8) (HGPRT
AKR1B1	ALDR_HUMAN	P15121	2	0.7665	Aldose reductase (EC 1.1.1.21) (AR) (Aldehyde reductase)
HK2	HXK2_HUMAN	P52789	2	0.8157	Hexokinase-2 (EC 2.7.1.1) (Hexokinase type II) (HK II) (Muscle for
NME4	NDKM_HUMAN	O00746	3	0.8706	Nucleoside diphosphate kinase, mitochondrial precursor (EC 2.7.4.6
POLR2G	RPB7_HUMAN	P62487	5	0.891	DNA-directed RNA polymerase II subunit RPB7 (RNA polymerase II subuni
PRPF4	PRP4_HUMAN	O43172	3	0.9188	U4/U6 small nuclear ribonucleoprotein Prp4 (U4/U6 snRNP 60 kD
NCBP2	NCBP2_HUMAN	P52298	3	0.9735	Nuclear cap-binding protein subunit 2 (20 kDa nuclear cap-bindin
AGPS	ADAS_HUMAN	O00116	1	1	Alkyldihydroxyacetonephosphate synthase, peroxisomal precurso
PLOD2	PLOD2_HUMAN	O00469	1	1	Procollagen-lysine,2-oxoglutarate 5-dioxygenase 2 precurso
APOL1	APOL1_HUMAN	O14791	1	1	Apolipoprotein-L1 precursor (Apolipoprotein L-I) (Apolipoprotein L
CASK	CSKP_HUMAN	O14936	1	1	Peripheral plasma membrane protein CASK (EC 2.7.11.1) (hCASK
KIF3B	KIF3B_HUMAN	O15066	1	1	Kinesin-like protein KIF3B (Microtubule plus end-directed kinesi
ARPC1B	ARC1B_HUMAN	O15143	1	1	Actin-related protein 2/3 complex subunit 1B (ARP2/3 complex 41 kD
LAMA5	LAMA5_HUMAN	O15230	1	1	Laminin subunit alpha-5 precursor
P4HA2	P4HA2_HUMAN	O15460	1	1	Prolyl 4-hydroxylase subunit alpha-2 precursor (EC 1.14.11.2) (4-P
YKT6	YKT6_HUMAN	O15498	1	1	Synaptobrevin homolog YKT6 (EC 2.3.1.-)
CALU	CALU_HUMAN	O43852	1	1	Calumenin precursor (Crocalbin) (IEF SSP 9302)
NUAK1	NUAK1_HUMAN	O60285	1	1	NUAK family SNF1-like kinase 1 (EC 2.7.11.1) (AMPK-related protei
FZD6	FZD6_HUMAN	O60353	1	1	Frizzled-6 precursor (Fz-6) (hFz6)
TPST1	TPST1_HUMAN	O60507	1	1	Protein-tyrosine sulfotransferase 1 (EC 2.8.2.20) (Tyrosylprotei
TLR2	TLR2_HUMAN	O60603	1	1	Toll-like receptor 2 precursor (Toll/interleukin 1 receptor-lik
B4GALT2	B4GT2_HUMAN	O60909	1	1	Beta-1,4-galactosyltransferase 2 (EC 2.4.1.-) (Beta-1,4-GalTase 2
NUP155	NU155_HUMAN	O75694	1	1	Nuclear pore complex protein Nup155 (Nucleoporin Nup155) (155 kD
KLF7	KLF7_HUMAN	O75840	1	1	Krueppel-like factor 7 (Ubiquitous krueppel-like factor)
PAK3	PAK3_HUMAN	O75914	1	1	Serine/threonine-protein kinase PAK 3 (EC 2.7.11.1) (p21-activate
TRIO	TRIO_HUMAN	O75962	1	1	Triple functional domain protein (EC 2.7.11.1) (PTPRF-interactin
PFTK1	PFTK1_HUMAN	O94921	1	1	Serine/threonine-protein kinase PFTAIRE-1 (EC 2.7.11.22)
YIF1A	YIF1A_HUMAN	O95070	1	1	Protein YIF1A (YIP1-interacting factor homolog A) (54TMp)

Up-regulated gene in HNSCC	Corresponding Swissprot entry name in PPIN	Corre-sponding Swissprot accession number in PPIN	Observed number of protein inter-action partners	Un-adjusted prob-ability	Gene name
ZWINT	ZWINT_HUMAN	O95229	1	1	ZW10 interactor (ZW10-interacting protein 1) (Zwint-1)
ACTL6A	ACL6A_HUMAN	O96019	1	1	Actin-like protein 6A (53 kDa BRG1-associated factor A) (Actin-relate
ADA	ADA_HUMAN	P00813	1	1	Adenosine deaminase (EC 3.5.4.4) (Adenosine aminohydrolase)
CST3	CYTC_HUMAN	P01034	1	1	Cystatin-C precursor (Cystatin-3) (Neuroendocrine basic polypeptide
TFRC	TFR1_HUMAN	P02786	1	1	Transferrin receptor protein 1 (TfR1) (TR) (TfR) (Trfr) (CD71 antigen
TK1	KITH_HUMAN	P04183	1	1	Thymidine kinase, cytosolic (EC 2.7.1.21)
ISG15	UCRP_HUMAN	P05161	1	1	Interferon-induced 17 kDa protein precursor [Contains: Ubiquiti
COL5A2	CO5A2_HUMAN	P05997	1	1	Collagen alpha-2(V) chain precursor
NEFL	NFL_HUMAN	P07196	1	1	Neurofilament light polypeptide (NF-L) (Neurofilament triplet
H1F0	H10_HUMAN	P07305	1	1	Histone H1.0 (Histone H1(0)) (Histone H1')
RHOC	RHOC_HUMAN	P08134	1	1	Rho-related GTP-binding protein RhoC precursor (H9)
MMP3	MMP3_HUMAN	P08254	1	1	Stromelysin-1 precursor (EC 3.4.24.17) (Matrix metalloproteinase-3
MMP10	MMP10_HUMAN	P09238	1	1	Stromelysin-2 precursor (EC 3.4.24.22) (Matrix metalloproteinase-10
LGALS1	LEG1_HUMAN	P09382	1	1	Galectin-1 (Lectin galactoside-binding soluble 1) (Beta-galactoside
RBP1	RET1_HUMAN	P09455	1	1	Retinol-binding protein I, cellular (Cellular retinol-binding protein
TPM1	TPM1_HUMAN	P09493	1	1	Tropomyosin alpha-1 chain (Tropomyosin-1) (Alpha-tropomyosin)
HOXB7	HXB7_HUMAN	P09629	1	1	Homeobox protein Hox-B7 (Hox-2C) (HHO.C1)
IL8	IL8_HUMAN	P10145	1	1	Interleukin-8 precursor (IL-8) (CXCL8) (Monocyte-derived neutrophi
RRAS	RRAS_HUMAN	P10301	1	1	Ras-related protein R-Ras precursor (p23)
UMPS	PYR5_HUMAN	P11172	1	1	Uridine 5'-monophosphate synthase (UMP synthase) [Includes: Orotat
TOP2A	TOP2A_HUMAN	P11388	1	1	DNA topoisomerase 2-alpha (EC 5.99.1.3) (DNA topoisomerase II, alph
COL11A1	COBA1_HUMAN	P12107	1	1	Collagen alpha-1(XI) chain precursor
COL6A2	CO6A2_HUMAN	P12110	1	1	Collagen alpha-2(VI) chain precursor
PTHLH	PTHR_HUMAN	P12272	1	1	Parathyroid hormone-related protein precursor (PTH-rP) (PTHrP
ACTN1	ACTN1_HUMAN	P12814	1	1	Alpha-actinin-1 (Alpha-actinin cytoskeletal isoform) (Non-muscl
P4HA1	P4HA1_HUMAN	P13674	1	1	Prolyl 4-hydroxylase subunit alpha-1 precursor (EC 1.14.11.2) (4-P
ACP5	PPA5_HUMAN	P13686	1	1	Tartrate-resistant acid phosphatase type 5 precursor (EC 3.1.3.2) (TR
MTHFD2	MTDC_HUMAN	P13995	1	1	Bifunctional methylenetetrahydrofolate dehydrogenase/cyclohydrolase
VEGFA	VEGFA_HUMAN	P15692	1	1	Vascular endothelial growth factor A precursor (VEGF-A) (Vascula
TCF3	TFE2_HUMAN	P15923	1	1	Transcription factor E2-alpha (Immunoglobulin enhancer-binding facto

Up-regulated gene in HNSCC	Corresponding Swissprot entry name in PPIN	Corre-sponding Swissprot accession number in PPIN	Observed number of protein inter-action partners	Un-adjusted prob-ability	Gene name
GJA1	CXA1_HUMAN	P17302	1	1	Gap junction alpha-1 protein (Connexin-43) (Cx43) (Gap junction 43 kD
GM2A	SAP3_HUMAN	P17900	1	1	Ganglioside GM2 activator precursor (GM2-AP) (Cerebroside sulfat
IGFBP3	IBP3_HUMAN	P17936	1	1	Insulin-like growth factor-binding protein 3 precursor (IGFBP-3) (IBP
FST	FST_HUMAN	P19883	1	1	Follistatin precursor (FS) (Activin-binding protein)
EFNA1	EFNA1_HUMAN	P20827	1	1	Ephrin-A1 precursor (EPH-related receptor tyrosine kinase ligand 1
CDH3	CADH3_HUMAN	P22223	1	1	Cadherin-3 precursor (Placental-cadherin) (P-cadherin)
CAD	PYR1_HUMAN	P27708	1	1	CAD protein [Includes: Glutamine-dependent carbamoyl-phosphat
LOX	LYOX_HUMAN	P28300	1	1	Protein-lysine 6-oxidase precursor (EC 1.4.3.13) (Lysyl oxidase)
CCND2	CCND2_HUMAN	P30279	1	1	G1/S-specific cyclin-D2
HLA-F	HLAF_HUMAN	P30511	1	1	HLA class I histocompatibility antigen, alpha chain F precursor (HLA
PFN2	PROF2_HUMAN	P35080	1	1	Profilin-2 (Profilin II)
FBN2	FBN2_HUMAN	P35556	1	1	Fibrillin-2 precursor
MYH10	MYH10_HUMAN	P35580	1	1	Myosin-10 (Myosin heavy chain 10) (Myosin heavy chain, nonmuscle IIb
FEN1	FEN1_HUMAN	P39748	1	1	Flap endonuclease 1 (EC 3.1.-.-) (Flap structure-specific endonucleas
EIF2S3	IF2G_HUMAN	P41091	1	1	Eukaryotic translation initiation factor 2 subunit 3 (Eukaryoti
TNFSF9	TNFL9_HUMAN	P41273	1	1	Tumor necrosis factor ligand superfamily member 9 (4-1BB ligand) (4
MAGEA4	MAGA4_HUMAN	P43358	1	1	Melanoma-associated antigen 4 (MAGE-4 antigen) (MAGE-X2) (MAGE-41)
MMP13	MMP13_HUMAN	P45452	1	1	Collagenase 3 precursor (EC 3.4.24.-) (Matrix metalloproteinase-13
ABCG1	ABCG1_HUMAN	P45844	1	1	ATP-binding cassette sub-family G member 1 (White protein homolog
SLC6A8	SC6A8_HUMAN	P48029	1	1	Sodium- and chloride-dependent creatine transporter 1 (CT1) (Creatin
HSPA13	STCH_HUMAN	P48723	1	1	Stress 70 protein chaperone microsome-associated 60 kDa protei
MARCKSL1	MRP_HUMAN	P49006	1	1	MARCKS-related protein (MARCKS-like protein 1) (Macrophag
VEGFC	VEGFC_HUMAN	P49767	1	1	Vascular endothelial growth factor C precursor (VEGF-C) (Vascula
MRE11A	MRE11_HUMAN	P49959	1	1	Double-strand break repair protein MRE11A (MRE11 homolog 1) (MRE1
ERF	ERF_HUMAN	P50548	1	1	ETS domain-containing transcription factor ERF (Ets2 represso
PPT1	PPT1_HUMAN	P50897	1	1	Palmitoyl-protein thioesterase 1 precursor (EC 3.1.2.22) (PPT-1
CAV2	CAV2_HUMAN	P51636	1	1	Caveolin-2
MSH6	MSH6_HUMAN	P52701	1	1	DNA mismatch repair protein MSH6 (MutS-alpha 160 kDa subunit) (G/
HMGA2	HMGA2_HUMAN	P52926	1	1	High mobility group protein HMGI-C (High mobility group AT-hoo
CTSC	CATC_HUMAN	P53634	1	1	Dipeptidyl-peptidase 1 precursor (EC 3.4.14.1) (Dipeptidyl-peptidas

Up-regulated gene in HNSCC	Corresponding Swissprot entry name in PPIN	Corre-sponding Swissprot accession number in PPIN	Observed number of protein inter-action partners	Un-adjusted prob-ability	Gene name
MAPK10	MK10_HUMAN	P53779	1	1	Mitogen-activated protein kinase 10 (EC 2.7.11.24) (Stress-activate
BCAT1	BCAT1_HUMAN	P54687	1	1	Branched-chain-amino-acid aminotransferase, cytosolic (EC 2.6.1.42
RAD23A	RD23A_HUMAN	P54725	1	1	UV excision repair protein RAD23 homolog A (hHR23A)
CDH11	CAD11_HUMAN	P55287	1	1	Cadherin-11 precursor (Osteoblast-cadherin) (OB-cadherin) (OSF-4)
CDH13	CAD13_HUMAN	P55290	1	1	Cadherin-13 precursor (Truncated-cadherin) (T-cadherin) (T-cad
RRAS2	RRAS2_HUMAN	P62070	1	1	Ras-related protein R-Ras2 precursor (Ras-like protein TC21
RHOB	RHOB_HUMAN	P62745	1	1	Rho-related GTP-binding protein RhoB precursor (H6)
HIST1H2BC	H2B1C_HUMAN	P62807	1	1	Histone H2B type 1-C/E/F/G/I (H2B.a/g/h/k/l) (H2B.1 A) (H2B/a) (H2B/g
IFI35	IN35_HUMAN	P80217	1	1	Interferon-induced 35 kDa protein (IFP 35)
TMF1	TMF1_HUMAN	P82094	1	1	TATA element modulatory factor (TMF)
EFNB1	EFNB1_HUMAN	P98172	1	1	Ephrin-B1 precursor (EPH-related receptor tyrosine kinase ligand 2
RUNX1	RUNX1_HUMAN	Q01196	1	1	Runt-related transcription factor 1 (Core-binding factor, alpha
LMNB2	LMNB2_HUMAN	Q03252	1	1	Lamin-B2
COL10A1	COAA1_HUMAN	Q03692	1	1	Collagen alpha-1(X) chain precursor
SOX4	SOX4_HUMAN	Q06945	1	1	Transcription factor SOX-4
BAX	BAXB_HUMAN	Q07814	1	1	Apoptosis regulator BAX, cytoplasmic isoform beta
GALNT2	GALT2_HUMAN	Q10471	1	1	Polypeptide N-acetylgalactosaminyltransferase 2 (EC 2.4.1.41
PPFIA1	LIPA1_HUMAN	Q13136	1	1	Liprin-alpha-1 (Protein tyrosine phosphatase receptor type
FADD	FADD_HUMAN	Q13158	1	1	Protein FADD (FAS-associated death domain protein) (FAS-associatin
PAK2	PAK2_HUMAN	Q13177	1	1	Serine/threonine-protein kinase PAK 2 (EC 2.7.11.1) (p21-activate
STK3	STK3_HUMAN	Q13188	1	1	Serine/threonine-protein kinase 3 (EC 2.7.11.1) (STE20-like kinas
TUSC3	TUSC3_HUMAN	Q13454	1	1	Tumor suppressor candidate 3 (Protein N33)
BIRC2	BIRC2_HUMAN	Q13490	1	1	Baculoviral IAP repeat-containing protein 2 (Inhibitor of apoptosi
TPBG	TPBG_HUMAN	Q13641	1	1	Trophoblast glycoprotein precursor (5T4 oncofetal trophoblas
RIN1	RIN1_HUMAN	Q13671	1	1	Ras and Rab interactor 1 (Ras interaction/interference protein 1) (Ra
NID2	NID2_HUMAN	Q14112	1	1	Nidogen-2 precursor (NID-2) (Osteonidogen)
DSG2	DSG2_HUMAN	Q14126	1	1	Desmoglein-2 precursor (HDGC)
GNA13	GNA13_HUMAN	Q14344	1	1	Guanine nucleotide-binding protein alpha-13 subunit (G alpha-13)
ITPR3	ITPR3_HUMAN	Q14573	1	1	Inositol 1,4,5-trisphosphate receptor type 3 (Type 3 inositol 1,4,5
CUL7	CUL7_HUMAN	Q14999	1	1	Cullin-7 (CUL-7)

Up-regulated gene in HNSCC	Corresponding Swissprot entry name in PPIN	Corre-sponding Swissprot accession number in PPIN	Observed number of protein inter-action partners	Un-adjusted prob-ability	Gene name
TRAM2	TRAM2_HUMAN	Q15035	1	1	Translocation-associated membrane protein 2
PDK1	PDK1_HUMAN	Q15118	1	1	[Pyruvate dehydrogenase [lipoamide]] kinase isozyme 1, mitochondria
TRIP13	TRP13_HUMAN	Q15645	1	1	Thyroid receptor-interacting protein 13 (TRIP-13) (Thyroid hormon
BAK1	BAK_HUMAN	Q16611	1	1	Bcl-2 homologous antagonist/killer (Apoptosis regulator BAK) (Bcl-2
FSCN1	FSCN1_HUMAN	Q16658	1	1	Fascin (Singed-like protein) (55 kDa actin-bundling protein) (p55)
UBE2S	UBE2S_HUMAN	Q16763	1	1	Ubiquitin-conjugating enzyme E2 S (EC 6.3.2.19) (Ubiquitin-protei
HIST2H2BE	H2B2E_HUMAN	Q16778	1	1	Histone H2B type 2-E (H2B.q) (H2B/q) (H2B-GL105)
ALG3	ALG3_HUMAN	Q92685	1	1	Dolichyl-P-Man:Man(5)GlcNAc(2)-PP-dolichyl mannosyltransferas
GGH	GGH_HUMAN	Q92820	1	1	Gamma-glutamyl hydrolase precursor (EC 3.4.19.9) (Gamma-Glu-
PAWR	PAWR_HUMAN	Q96IZ0	1	1	PRKC apoptosis WT1 regulator protein (Prostate apoptosis response
GPX7	GPX7_HUMAN	Q96SL4	1	1	Glutathione peroxidase 7 precursor (EC 1.11.1.9) (CL683)
KIF2C	KIF2C_HUMAN	Q99661	1	1	Kinesin-like protein KIF2C (Mitotic centromere-associated kinesin
FEZ1	FEZ1_HUMAN	Q99689	1	1	Fasciculation and elongation protein zeta 1 (Zygin-1) (Zygin I)
SEMA3C	SEM3C_HUMAN	Q99985	1	1	Semaphorin-3C precursor (Semaphorin E) (Sema E)
KCNS3	KCNS3_HUMAN	Q9BQ31	1	1	Potassium voltage-gated channel subfamily S member 3 (Voltage-gate
BTBD2	BTBD2_HUMAN	Q9BX70	1	1	BTB/POZ domain-containing protein 2
UCK2	UCK2_HUMAN	Q9BZX2	1	1	Uridine-cytidine kinase 2 (EC 2.7.1.48) (UCK 2) (Uridin
SMURF2	SMUF2_HUMAN	Q9HAU4	1	1	E3 ubiquitin-protein ligase SMURF2 (EC 6.3.2.-) (Smad ubiquitinatio
MYO10	MYO10_HUMAN	Q9HD67	1	1	Myosin-X (Unconventional myosin-10)
PDLIM7	PDLI7_HUMAN	Q9NR12	1	1	PDZ and LIM domain protein 7 (LIM mineralization protein) (LMP
HOMER3	HOME3_HUMAN	Q9NSC5	1	1	Homer protein homolog 3 (Homer-3)
SCHIP1	SCHI1_HUMAN	Q9P0W5	1	1	Schwannomin-interacting protein 1 (SCHIP-1)
STK17A	ST17A_HUMAN	Q9UEE5	1	1	Serine/threonine-protein kinase 17A (EC 2.7.11.1) (DAP kinase-relate
PROCR	EPCR_HUMAN	Q9UNN8	1	1	Endothelial protein C receptor precursor (Endothelial cell protein
NCDN	Q9Y4D9_HUMAN	Q9Y4D9	1	1	Neurochondrin (Fragment)
MYO5A	MYO5A_HUMAN	Q9Y4I1	1	1	Myosin-Va (Dilute myosin heavy chain, non-muscle) (Myosin-12) (Myosi
LOXL2	LOXL2_HUMAN	Q9Y4K0	1	1	Lysyl oxidase homolog 2 precursor (EC 1.4.3.-) (Lysyl oxidase-lik
LSM4	LSM4_HUMAN	Q9Y4Z0	1	1	U6 snRNA-associated Sm-like protein LSm4 (Glycine-rich protein) (GRP)
CLIC4	CLIC4_HUMAN	Q9Y696	1	1	Chloride intracellular channel protein 4 (Intracellular chloride io

Supporting Table 12. Detailed references for the protein-protein interactions between the top 56 prioritized proteins and their interactors among the 260 proteins derived from the upregulated HSNCC genes in GSE6631 (Network shown in **Figure 3**, count of interactors in **Table S11**, **Methods**).

First biomolecule of the interaction		Second biomolecule of the interaction		Reference(s) in support of the Protein Interaction (Pubmed ids and Source dataset preceded by @)
Protein (Uniprot)	Gene (HGNC)	Protein (Uniprot)	Gene (HGNC)	
O00762	UBE2C	O14965	AURKA	@REACTOME
O00762	UBE2C	O60566	BUB1B	11535616,11285280,11742988 @REACTOME
O00762	UBE2C	P06493	CDC2	10679238,11285280,11285280,11859075,14657031 @REACTOME
O00762	UBE2C	P14635	CCNB1	11285280,11859075,14657031 @REACTOME
O00762	UBE2C	P49721	PSMB2	11285280,12070128,15029244 @REACTOME
O00762	UBE2C	Q12834	CDC20	11285280,11535616,11742988,12070128 @REACTOME
O00762	UBE2C	Q13200	PSMD2	11285280,12070128,15029244 @REACTOME
O00762	UBE2C	Q96GD4	AURKB	@REACTOME
O14965	AURKA	P49721	PSMB2	@REACTOME
O14965	AURKA	Q12834	CDC20	10377410 @BIOGRID; @REACTOME
O14965	AURKA	Q13200	PSMD2	@REACTOME
O14965	AURKA	Q96GD4	AURKB	@REACTOME
O14965	AURKA	Q9ULW0	TPX2	12177045 @BIOGRID
O15427	SLC16A3	P35613	BSG	10921872 @BIOGRID
O60566	BUB1B	P06493	CDC2	10679238,11285280,11285280 @REACTOME
O60566	BUB1B	P49721	PSMB2	11285280 @REACTOME
O60566	BUB1B	P63010	AP2B1	12419313,16189514 @BIOGRID
O60566	BUB1B	Q12834	CDC20	11030144,11274370,11535616,11907259 @BIOGRID;11285280,11535616,11742988 @REACTOME
O60566	BUB1B	Q13200	PSMD2	11285280 @REACTOME
O95067	CCNB2	P06493	CDC2	1717476 @REACTOME;9926943 @BIOGRID; 9926943 @HPRD; @REACTOME
O95067	CCNB2	P30305	CDC25B	@REACTOME
P00533	EGFR	P08648	ITGA5	10888683 @BIOGRID
P00533	EGFR	P09619	PDGFRB	9506992 @BIOGRID
P00533	EGFR	P16070	CD44	12093135 @BIOGRID;12093135 @HPRD
P00533	EGFR	P24821	TNC	11470832 @BIOGRID
P00533	EGFR	P29353	SHC1	12577067 @BIOGRID;9544989,8887653,12577067 @HPRD;9544989 @BIOGRID
P00533	EGFR	P42224	STAT1	12070153 @BIOGRID
P00533	EGFR	Q04695	KRT17	12577067 @BIOGRID
P00533	EGFR	Q05397	PTK2	10806474 @BIOGRID
P00533	EGFR	Q13191	CBLB	10086340 @BIOGRID

First biomolecule of the interaction		Second biomolecule of the interaction		Reference(s) in support of the Protein Interaction (Pubmed ids and Source dataset preceded by @)
Protein (Uniprot)	Gene (HGNC)	Protein (Uniprot)	Gene (HGNC)	
P00533	EGFR	Q15149	PLEC1	12577067 @BIOGRID; 12577067 @HPRD
P00749	PLAU	P05121	SERPINE1	2161846 @BIOGRID;2544876 @REACTOME
P00749	PLAU	P14543	NID1	1499567 @BIOGRID;1499567 @HPRD
P00749	PLAU	Q03405	PLAUR	11501527,16189514,3031025 @BIOGRID;1846368,2156852,3023326,2156852,3023326,1846368 @REACTOME
P01137	TGFB1	P05067	APP	16189514 @BIOGRID
P01137	TGFB1	P06756	ITGAV	11970960 @BIOGRID
P01137	TGFB1	P09486	SPARC	15034927,16189514 @BIOGRID
P01137	TGFB1	P21810	BGN	10383378,16189514,8093006 @BIOGRID
P02452	COL1A1	P02751	FN1	8468356 @BIOGRID
P02452	COL1A1	P08123	COL1A2	9028946 @REACTOME; @REACTOME
P02452	COL1A1	P08648	ITGA5	16189514,1693626 @BIOGRID
P02452	COL1A1	P09486	SPARC	16189514,7034958 @BIOGRID
P02452	COL1A1	P14543	NID1	9733643 @BIOGRID
P02452	COL1A1	P14780	MMP9	16189514,9878537 @BIOGRID
P02452	COL1A1	P16070	CD44	16189514,1730778 @BIOGRID
P02452	COL1A1	P21810	BGN	16189514,7852349 @BIOGRID
P02452	COL1A1	Q02388	COL7A1	9169408 @BIOGRID
P02452	COL1A1	Q15582	TGFBI	11867580,12034705,16189514 @BIOGRID
P02461	COL3A1	P09486	SPARC	16189514,2745554 @BIOGRID
P02462	COL4A1	P02751	FN1	3997552 @BIOGRID
P02462	COL4A1	P05067	APP	9136074 @BIOGRID
P02462	COL4A1	P07093	SERPINE2	8006028 @BIOGRID
P02462	COL4A1	P08572	COL4A2	12011424 @BIOGRID
P02462	COL4A1	P14780	MMP9	9878537 @BIOGRID
P02462	COL4A1	Q02388	COL7A1	9169408 @BIOGRID
P02462	COL4A1	Q15582	TGFBI	11867580 @BIOGRID
P02751	FN1	P08123	COL1A2	8468356 @BIOGRID
P02751	FN1	P08572	COL4A2	3997552 @BIOGRID
P02751	FN1	P08648	ITGA5	11801679,9211865 @BIOGRID
P02751	FN1	P14780	MMP9	11134254 @BIOGRID
P02751	FN1	P24821	TNC	7499434 @BIOGRID
P02751	FN1	P26006	ITGA3	9733622 @BIOGRID
P02751	FN1	Q02388	COL7A1	7963647,9169408 @BIOGRID

First biomolecule of the interaction		Second biomolecule of the interaction		Reference(s) in support of the Protein Interaction (Pubmed ids and Source dataset preceded by @)
Protein (Uniprot)	Gene (HGNC)	Protein (Uniprot)	Gene (HGNC)	
P02751	FN1	Q15582	TGFBI	11867580,12034705 @BIOGRID
P03956	MMP1	P35613	BSG	10706100,16189514 @BIOGRID
P04085	PDGFA	P09486	SPARC	1311092 @BIOGRID
P04216	THY1	P06756	ITGAV	15897908 @HPRD
P05067	APP	P08123	COL1A2	7688497 @BIOGRID
P05067	APP	P08572	COL4A2	9136074 @BIOGRID
P05067	APP	P14543	NID1	11376898 @BIOGRID; @HPRD
P05067	APP	P21810	BGN	16189514,7793988 @BIOGRID;7793988 @HPRD
P05067	APP	P27797	CALR	11378243,16189514 @BIOGRID
P05067	APP	P29353	SHC1	11877420,9045663 @HPRD;11877420,16189514,9045663 @BIOGRID
P05067	APP	Q92870	APBB2	14527950,16189514,8855266 @BIOGRID;8855266,14527950 @HPRD
P05121	SERPINE1	Q03405	PLAUR	2544876 @REACTOME
P06400	RB1	P20248	CCNA2	@BIND
P06400	RB1	P24864	CCNE1	@BIND
P06400	RB1	P33993	MCM7	16189514,9566894 @BIOGRID; @BIND
P06400	RB1	P61024	CKS1B	@BIND
P06400	RB1	Q16667	CDKN3	@BIND
P06493	CDC2	P14635	CCNB1	10362260,1833185,10973963 @DIP;10395539,1384126,1717476,1828290,7799941,8440392,12411508,10827953,8119945,9670027,9679058 @REACTOME;2570636 @BIOGRID
P06493	CDC2	P20248	CCNA2	10924145 @BIOGRID;1833185 @DIP; @REACTOME
P06493	CDC2	P24864	CCNE1	1388288 @BIOGRID;1833068 @DIP
P06493	CDC2	P30305	CDC25B	10827953,12411508,8119945,8440392,1828290 @REACTOME;9585407,9733650,9268380,9141461,8440392,12107172 @HPRD
P06493	CDC2	P33552	CKS2	2227411 @HPRD
P06493	CDC2	P39880	CUX1	11584018 @HPRD
P06493	CDC2	P49721	PSMB2	11285280 @REACTOME
P06493	CDC2	P61024	CKS1B	16189514 @BIOGRID
P06493	CDC2	Q12834	CDC20	10679238,1128528011285280,12791267 @REACTOME
P06493	CDC2	Q13200	PSMD2	11285280 @REACTOME
P06493	CDC2	Q15149	PLEC1	8626512 @HPRD
P06493	CDC2	Q16667	CDKN3	8127873 @BIOGRID;8242750 @DIP
P06493	CDC2	Q96R06	SPAG5	11549262 @BIOGRID
P06493	CDC2	Q99640	PKMYT1	10373560,10504341,9001210,9268380,12912980 @HPRD;10504341 @BIOGRID; @REACTOME
P06756	ITGAV	P10451	SPP1	10835423 @BIOGRID

First biomolecule of the interaction		Second biomolecule of the interaction		Reference(s) in support of the Protein Interaction (Pubmed ids and Source dataset preceded by @)
Protein (Uniprot)	Gene (HGNC)	Protein (Uniprot)	Gene (HGNC)	
P06756	ITGAV	P14543	NID1	9686320 @HPRD
P06756	ITGAV	Q05397	PTK2	11927607 @BIOGRID
P07093	SERPINE2	P08572	COL4A2	8006028 @BIOGRID
P08069	IGF1R	P29353	SHC1	8776723 @BIOGRID;8776723 @HPRD; @BIND
P08123	COL1A2	P09486	SPARC	7034958 @BIOGRID
P08123	COL1A2	P14780	MMP9	9878537 @BIOGRID
P08123	COL1A2	P16070	CD44	1730778 @BIOGRID
P08123	COL1A2	P21810	BGN	7852349 @BIOGRID
P08123	COL1A2	P51884	LUM	10734230 @BIOGRID
P08123	COL1A2	Q15582	TGFBI	11867580,12034705 @BIOGRID
P08195	SLC3A2	Q01650	SLC7A5	11311135,9829974 @BIOGRID
P08572	COL4A2	P14780	MMP9	9878537 @BIOGRID
P08572	COL4A2	P21810	BGN	11259413 @BIOGRID
P08572	COL4A2	Q15582	TGFBI	11867580 @BIOGRID
P08648	ITGA5	P10451	SPP1	16189514,7592829 @BIOGRID
P08648	ITGA5	P24821	TNC	9314546 @BIOGRID
P09619	PDGFRB	P29353	SHC1	16189514,8195171 @BIOGRID; @BIND
P10244	MYBL2	P20248	CCNA2	10871850 @BIOGRID
P10253	GAA	P15121	AKR1B1	@KEGG
P10253	GAA	P52789	HK2	@KEGG
P10451	SPP1	P16070	CD44	10657301,12377945,16189514 @BIOGRID
P11047	LAMC1	P14543	NID1	9733643 @HPRD
P12318	FCGR2A	P29353	SHC1	10382761,8940017 @HPRD;10382761,16189514,8940017 @BIOGRID
P13497	BMP1	Q13751	LAMB3	10806203 @HPRD
P13497	BMP1	Q13753	LAMC2	10806203 @BIOGRID;10806203 @HPRD
P13497	BMP1	Q16787	LAMA3	10806203 @BIOGRID;10806203 @HPRD
P14543	NID1	Q13753	LAMC2	11733994 @HPRD
P14635	CCNB1	P30305	CDC25B	10827953,12411508,8119945,8440392,1828290 @REACTOME
P14635	CCNB1	Q12834	CDC20	11285280,12791267 @REACTOME
P14780	MMP9	P16070	CD44	10652271,16189514 @BIOGRID
P14780	MMP9	P35442	THBS2	10900205 @BIOGRID
P16144	ITGB4	P23229	ITGA6	9660880@ BIOGRID
P16144	ITGB4	P29353	SHC1	11044453 @BIOGRID
P16144	ITGB4	Q15149	PLEC1	11328943 @BIOGRID

First biomolecule of the interaction		Second biomolecule of the interaction		Reference(s) in support of the Protein Interaction (Pubmed ids and Source dataset preceded by @)
Protein (Uniprot)	Gene (HGNC)	Protein (Uniprot)	Gene (HGNC)	
P20248	CCNA2	P61024	CKS1B	@BIND
P20248	CCNA2	Q12834	CDC20	10679238 @BIOGRID
P21741	MDK	P42224	STAT1	9452495,16189514 @BIOGRID
P21810	BGN	P55001	MFAP2	11723132 @HPRD
P24864	CCNE1	P61024	CKS1B	@BIND
P24864	CCNE1	Q99640	PKMYT1	@REACTOME
P25205	MCM3	P33993	MCM7	16189514,7601140,8631321,10464337 @BIOGRID;10884341,12364596 @REACTOME
P25205	MCM3	P42224	STAT1	11248027,16189514 @BIOGRID
P25205	MCM3	P49736	MCM2	10884341,12364596 @REACTOME;16189514 @BIOGRID
P25205	MCM3	Q14566	MCM6	10884341,12364596 @REACTOME;16189514 @BIOGRID
P25205	MCM3	Q14691	GINS1	@REACTOME
P26006	ITGA3	P27797	CALR	11327697 @BIOGRID
P26006	ITGA3	P48509	CD151	14676841 @HPRD
P26006	ITGA3	Q16787	LAMA3	9950675,11278628 @BIOGRID
P26038	MSN	Q15149	PLEC1	11441066 @BIOGRID;11441066 @HPRD
P29353	SHC1	P35568	IRS1	7499194 @BIOGRID
P29353	SHC1	Q05397	PTK2	11980671,16189514 @BIOGRID
P29353	SHC1	Q05682	CALD1	10559276,16189514 @BIOGRID
P29353	SHC1	Q13191	CBLB	9614102,16189514 @BIOGRID
P33552	CKS2	P61024	CKS1B	2227411 @HPRD
P33993	MCM7	P49736	MCM2	10748114,12207017,16189514,10567526,8798650 @BIOGRID;10884341,12364596 @REACTOME
P33993	MCM7	Q14566	MCM6	10567526,10748114,12207017,12694531,8798650 @BIOGRID; @REACTOME
P33993	MCM7	Q14691	GINS1	@REACTOME
P35613	BSG	P53985	SLC16A1	10921872 @BIOGRID
P42224	STAT1	Q03405	PLAUR	@BIND
P42224	STAT1	Q05397	PTK2	11278462,16189514 @BIOGRID
P49450	CENPA	P68431	HIST1H3J	15282608 @BIOGRID
P49721	PSMB2	Q12834	CDC20	11285280, 12070128,15469984 @REACTOME
P49721	PSMB2	Q13200	PSMD2	@REACTOME
P49721	PSMB2	Q96GD4	AURKB	@REACTOME
P49736	MCM2	Q14566	MCM6	10884341,12364596 @REACTOME;12694531,16189514 @BIOGRID;@ BIND
P49736	MCM2	Q14691	GINS1	@REACTOME
P50281	MMP14	P51884	LUM	15466200 @BIOGRID
P61024	CKS1B	Q16667	CDKN3	@BIND

First biomolecule of the interaction		Second biomolecule of the interaction		Reference(s) in support of the Protein Interaction (Pubmed ids and Source dataset preceded by @)
Protein (Uniprot)	Gene (HGNC)	Protein (Uniprot)	Gene (HGNC)	
P68431	HIST1H3J	P83916	CBX1	11882902 @BIOGRID;11882902 @HPRD
P68431	HIST1H3J	Q96GD4	AURKB	10949293 @HPRD
Q02388	COL7A1	Q13753	LAMC2	9989793 @BIOGRID
Q03405	PLAUR	Q12884	FAP	12376466 @BIOGRID
Q12834	CDC20	Q13200	PSMD2	11285280,12070128,15469984 @REACTOME
Q12834	CDC20	Q96GD4	AURKB	@REACTOME
Q13200	PSMD2	Q96GD4	AURKB	@REACTOME
Q13751	LAMB3	Q13753	LAMC2	10964684 @HPRD
Q13751	LAMB3	Q16787	LAMA3	7775432 @HPRD
Q14566	MCM6	Q14691	GINS1	@REACTOME

Supporting Table 13. Primer sets used in real-time qRT-PCR for the determination of miR-204 target mRNA expression (related to Figure 2B-C, and Figures S4-S5).

Gene	GenBank accession	Forward	Reverse
ARPC1B	BC002988	cggatCACATCAAGAAGCCCATCcG	GCAGCCAGCAGCACATTGTT
ATP2B1	NM_001682	ATTGATGACACTGATGCCGAAGA	cgtaacGGGTGGAGGACTGGAGTTAcG
BMP1	AK291620	cggtaACGTCCCTGGACCTGTACcG	CCATCTCGGACCTCCACATAGTC
CDC25B	NM_021873	ACGAGACCGTGCTGTCAACG	cggcGGAAGAACTCCTTGTAGCcG
CDH11	NM_001797	CAGTACACGTTGATGGCTCAGG	cggagGGTCTCGTGCAGGAACTCcG
CTSC	BC109386	CCAACTGCACCTATCTTGACCTG	cgatgtATAACCGAGCAGTTGACATcG
EBN2	NM_004519	CCCTACTCGGACCGAATCTCC	cgcgGAGGTGTGTCACTGTCTCGcG
EFNB1	NM_004429	GCACCCTGTCTACATCGTCCA	cggcACCTTGTAGTAGATGTTCGCcG
FAP	NM_004460	AATTCAACTGTGATGGCAAGAGC	cggaacCACATTATCATCTGCTGTTCcG
HMGA2	NM_003483	cggctGTCTCTTCCCTTTCCAAGCcG	GGGTTGGGATCAGGAGTTGC
IGF2BP3	NM_006547	cgggCCATGACTCCTCCCTACCcG	TGGCACCGACTGATAGAGCTG
INHBA	NM_002192	ATTGGAAGGAGGGCAGAAATGA	cggagGGCAAACGTGATGATCTCcG
ITGB4	NM_001005619	CAGAGAAGCAGGTGGAACAGAGG	cggcGCAGTGAGGGTGTAGTAGCcG
LTBP1	NM_000627	cggtcCCCTACTTCATCCAAGACcG	CCGCATTCCTCAGCCTGTAAC
MMP3	NM_002422	GTTCCGCCTGTCTCAAGATGAT	cggaaGTTCTGGAGGGACAGGTTCcG
MMP9	AF538844	cggttAGTCAAGATGACATCTGAACcG	CCCTTGCACAGGAAGAAGCTC
MY010	AF060223	cgggtTACTTCATGCAGCTTCAGACCcG	GCAGCACTTGTAGAAGGTGGTCA
SHC1	NM_183001	GCTGCATCCCAACGACAAAG	cggtaTCCACACAACCCATGTACcG
SOX4	NM_003107	ATGTCCCTGGGCAGCTTCAGT	cgggCGAAGTTAAAATCCAGGTCCcG
SPARC	NM_003118	GAATTTGATGATGGTGCAGAGGA	cggaaGTTCTGGCAGGGATTTTCcG
TBP	NM_003194	cgaagtTGACCTAAAGACCATTGCACTTcG	CGTGGTTCGTGGCTCTCTTATC
TRPM3	NM_206948	cggacTCATTGGAAGAGATGTTGTCcG	CAGTGAGCTTGCTCATGGGATT
AURKB	NM_004217	GCTTTGCTATGAGCTGCTGGTG	cgcatTAGGTCCACCTTGACGATGcG
BIRC2	NM_001166	cgggTGTCTACATATTCAACTTTCCCcG	ACCAGCACGAGCAAGACTCCT
HOXB7	NM_004502	cggagCCTTTGAGCAGAACCTCTCcG	GTCAGTTCCTGAGCTTCGCATC
ERF	NM_006494	CCGCCACCACAGATCAAGGT	cgagtcCACCTCTACCTCCTCCGACTcG

Supporting Table 14. List of Abbreviations referred in the manuscript.

Abbreviation	Expanded description
APRC1B	Actin related Protein 2/3 complex, subunit 1B
AURKB	Aurora Kinase B
BIND	Biomolecular Interaction Network Database
BioGRID	General Repository for Interaction Datasets
BMC	Basement Membrane Complex
BMP1	Bone Morphogenetic Protein 1
BP	Biological Processes
BSA	Bovine Serum Albumin
CAGR	Cancer Associated Genomic Regions
CDC25B	Cell Division Cycle 25 homolog B
CDH11	Cadherin 11, type 2, OB-cadherin
Ct	PCR Cycle Treshold
CTSC	Carbohydrate Sulfotransferase 2
DIP	Database of Interacting Proteins
ECM	Extracellular Matrix
EGFR	Epidermal Growth Factor Receptor
FAP	Fibroblast Activation Protein, Alpha
FDR	False Discovery Rate
GFP	Green Fluoresence Protein
GO	Gene Ontology
GUSB	Glucuronidase, Beta
HNSCC	Squamous Cell Carcinoma of the Head and Neck
HPRD	Human Proteome Reference Database
HPRT1	Hypoxanthine Phosphoribosyltransferase 1
HUGO	Human Genome Organization
IACUC	Institutional Animal Care and Use Committee (according to U.S. federal law)
ID	Identifier
IMRE	Imputed microRNA Regulation based on Weighted Rank Expression and Putative microRNA Targets
IQR	Inter Quartile Range
ITGB4	Integrin, Beta 4
JSQ3	Head and Neck Cancer Cell Line
KEGG	Kyoto Encyclopedia of Genes and Genomes
LOH	Loss of Heterozygosity
LSM	Lexico-Semantic Mapping
MF	Molecular Functions
MINT	Molecular INTeraction database
miRNOME	Five complementary microRNA target datasets integrated together
MMP9	Matrix Metallopeptidase 9
MTT	MTT Cell Proliferation asssay
OMIM	Online Mendelian Inheritance in Man
OncoMIM	Cancer-related Clinical Terms and MIM Genes
OPP	Optimized Partial Mapping

**Abbreviation**	**Expanded description**
PCR	Polymerase Chain Reaction
PPIA	Peptidylprolyl Isomerase A (cyclophilin A)
PPIN	Protein-Protein Interaction Network
qPCR	Quantitative PCR
RM	Regional Minimum
RoL	Review of Literature
RRM	Refinded Regional Minimum
SAM	Significant Analysis of Microarray
SDRRM	Significant Descendants of Refined Regional Minimum
SE	Standard Error
SHC1	SHC transforming protein 1
SNOMED	Systematized Nomenclature of Medicine
SQ38	Head and Neck Cancer Cell Line [Cancer Res, 1992; 52(19):5299]
TRPM3	Transient Receptor Potential Melastatin 3 cation channel
WRE	Weighted Rank Expression
