# Supplementary material for: Network Modeling Identifies Molecular Functions Targeted by miR-204 to Suppress Head and Neck Tumor Metastasis
Source: PLoS Comput Biol. 2010 Apr 1;6(4):e1000730. doi: 10.1371/journal.pcbi.1000730 (PMC2848541; doi:10.1371/journal.pcbi.1000730)
Supplement: Protocol S1 — Supplementary Protocols (sections A–D) (0.10 MB PDF) [file pcbi.1000730.s003.pdf]

Protocol S1

Section A. Generation of OncoMIM Gene-phenotype database (Material and Methods, Table 3 in Text S2, Supporting Figure 8 in Text S1, and **Dataset S1).**

We conducted a statistical enrichment analysis of the inheritable cancer genes found in the OMIM human disease gene database [Online Mendelian Inheritance in Man, http://www.ncbi.nlm.nih.gov/omim/ (downloaded Dec. 1, 2006)]. We downloaded files "omim.txt.Z" and "genemap" files at http://www.ncbi.nlm.nih.gov/Omim/omimfaq.html#download on May. 17, 2007. Thereafter, we extracted the unique identifier of each disease gene or clinical finding (**MIM**), title term, alternate title terms, clinical synopsis terms, or the allelic variant terms when they were available. OMIM clinical terms are textual, unstructured, and do not have unique identifiers themselves. In order to standardize these OMIM "terms", we computationally mapped them to 17,182 identifiers from the Systematized Nomenclature of Medicine {SNOMED CT version July 31, 2005 [1] (see Lexical terminology mapping, below)}. The SNOMED ontology allowed also to recover relationship between related cancer phenotypes and use enrichment statistics that take advantage of these classifications as they do in Gene Ontology (see **Equation 4** in **Material and Methods**, **Protocol S1/Section B**, and **Supporting Figure 8 in Text S1**). These clinical OMIM terms were thus encoded in SNOMED's anatomies, morphologies, clinical findings and disease. In addition, the following 17 relationships were extracted from SNOMED to define disease-anatomical, disease-morphology and parent-child relationships: "IS-A", "Associated morphology", "Specimen source morphology", "Specimen source topography", "Part of, Associated finding", "Component", "After", "Finding site", "Direct morphology", "Has focus", "Procedure site", "Has definitional manifestation", "Direct Procedure site", "Due to", and "Associated with". The resulting dataset, OncoMIM, consists of well-organized cancer-related clinical terms and MIM genes and is provided as **Dataset S1**. We utilized the Lexico-Semantic Mapping (**LSM**) technology that we developed to identify associations between human anatomical entities in the SNOMED and the OMIM [2-4]. The mapping process was achieved through the following steps: ***(i) the development of a lexical filter over OMIM terms***. After systematic examination by clinicians of the description and synonyms of the SNOMED, and the title and clinical synopsis of the OMIM, semantic filters were applied before conducting lexical mapping to filter out erroneous mapping between the SNOMED and OMIM. The categories of the filters include OMIM titles containing no clinical description (i.e. zinc finger protein), general SNOMED entities irrelevant to the study, SNOMED entities pertaining to animal anatomy (i.e. "non-human body structure"), and ambiguous SNOMED entities (i.e. "unspecified nutritional deficiency"). (***ii***) ***Normalization of text***. After the filtering process, the terms were standardized using the Norm algorithm [5]: the titles, alternate titles, and clinical synopsis of OMIM as well as the anatomies, morphologies, clinical findings, and diseases of the SNOMED. ***(iii) Lexicon terminology mapping*****.** Two types of lexicon string mapping were performed. First, identical term mapping was

conducted between OMIM's terms and those of SNOMED. Second, optimized partial mapping (**OPP**) was conducted (OPP Software is provided upon request). Our algorithm identifies the entire normalized terms in relevant SNOMED CT categories (anatomy, morphology, clinical finding, and disease) that can be mapped to the entire OMIM term or a part of this term. After merging the results of these two types of mappings, we classified 7,589 distinct clinical OMIM terms in 2,553 SNOMED entities categorized as 'Anatomy and Morphology', and 7,649 distinct clinical OMIM terms in 6,558 distinct SNOMED entities categorized as 'Clinical finding and Disease'. Using SNOMED's classification, we identified 610 distinct OMIM genes that are associated with 438 and 606 distinct cancer-related identifiers in SNOMED's morphologies and diseases classification, respectively (subsumed by SNOMED ID#108369006 Neoplasm morphology and SNOMED ID#55342001 Neoplastic disease). Using the hierarchical classification of SNOMED, we further categorized these genes in each relevant cancer category. The OncoMIM dataset containing MIM codes to SNOMED codes can be downloaded from **Dataset S1**.

References of Protocol S1 Section A

1. Spackman KA, Campbell KE, Cote RA (1997) SNOMED RT: a reference terminology for health care. Proc AMIA Annu Fall Symp: 640-644.
2. Friedman C, Borlawsky T, Shagina L, Xing HR, Lussier YA (2006) Bio-Ontology and text: bridging the modeling gap. Bioinformatics 22: 2421-2429.
3. Lussier Y, Borlawsky T, Rappaport D, Liu Y, Friedman C (2006) PhenoGO: assigning phenotypic context to gene ontology annotations with natural language processing. Pac Symp Biocomput: 64-75.
4. Lussier YA, Li J (2004) Terminological mapping for high throughput comparative biology of phenotypes. Pac Symp Biocomput: 202-213.
5. Browne AC, Divita G, Aronson AR, McCray AT (2003) UMLS language and vocabulary tools. AMIA Annu Symp Proc: 798.

<u>Section B</u>. Refinement of the hierarchical *P*-values in enrichments of GO or of SNOMED terms (Table 1 and Table 7 in Text S2, Supporting Figure 8 in Text S1).

In this study, we conducted enrichment statistics over two ontologies: Gene Ontology and SNOMED. These ontologies can be represented as directed acyclic direct graphs composed of nodes (classes of genes in the ontology such as a GO term or a SNOMED term) and edges (relationships to classes in the ontology) [1]. We developed a refinement algorithm to identify and filter out false positive p-values derived from enrichment studies in ontologies (hierarchical classifications) due to the inheritance of genes in ancestry classes of a significantly enriched class, a rarely mentioned problem of enrichment statistics that has also been reported by others [2,3]. In this manuscript, this algorithm identified from 0% to 59.3% of false positive enrichment results, with an average of 30.2% per enrichment (data not shown).

Equations S1, S2 and **S3** describe our refinement algorithm over the statistically significant results in an enrichment study. The ontologies are viewed as directed acyclic graphs where nodes are the entities (e.g. Gene Ontology terms or SNOMED concepts) and edges of the graphs are hierarchical relationships between these entities. In our enrichment studies, genes are classified to these entities/nodes. Nodes must meet the following two inclusion criteria: (i) the adjusted *P*-value of their gene enrichment (**Equation 4**, **Material and Methods**) is significant (adjusted $P \leq 0.05$), and (ii) the number of genes classified to this entity/node ≥ 3.

Definitions: V is the set of nodes, and E is the set of edges. Each node, $v_i \in V$, is assigned two types of descriptors of its relation with its neighboring nodes: descriptors of v_i are (i) one or more edges notes as $e_{i,j} \in E$ (when v_i is parent of v_j) or $e_{j,i} \in E$ (when v_j is parent of v_i), and (ii) an adjusted *P*-value from the enrichment study symbolized as p_i (**Equation 4**, **Material and Methods**). Each node, v_i, is also defined in terms of " sets" of hierarchical relationships (capital letters) as follow: (i) A_i for all parent nodes (1st degree <u>a</u>ncestors), (ii) C_i for all <u>c</u>hildren, and (iii) D_i for all <u>d</u>escendents. These hierarchical sets are respectively described as $A_i = \{v_j \in V \,|\, \exists e_{j,i} \in E\}$, $C_i = \{v_j \in V \,|\, \exists e_{i,j} \in E\}$ and $D_i = \{v_j \in V \,|\, \exists e_{i,k_1}, e_{k_1,k_2}, \ldots, e_{k_{n-1},k_n}, e_{k_n,j} \in E\}$. The nodes that have the most statistically significant adjusted *P*-values (lower values) as compared to their hierarchic neighbors were identified as "Regional Minimum", noted V_{RM}, as defined in **Equation S1**. Among Regional Minimum nodes, we further excluded parents that have the same adjusted *P*-values as their children to conserve the most informative nodes: Refined Regional Minimum (V_{RRM}, **Equation S2**). The subsumed significant associations (Significant Descendants of Refined Regional Minimum or SDRRM) are defined in **Equation S3**. Finally, **Equation S4** defines the subset of included nodes (retained nodes) after refinement: those found in either **Equation S2** or **Equation S3**.

$$V_{RM} = \{ v_i \in V \mid \forall v_j \in A_i \cup C_i, p_j \geq p_i \}$$ (**Equation S1**)

$$V_{RRM} = \{ v_i \in V \mid \exists v_j \in V_{RM}, v_j \in A_i, v_j \in V_{RM} \}$$ (**Equation S2**)

$$V_{SDRRM} = \{ v_i \in V \mid \exists v_j \in V_{RRM}, v_i \in D_j, v_i \notin V_{RRM}, v_i \notin V_{RM} \}$$ (**Equation S3**)

$$V_{included} = \{ v_i \in V \mid v_i \in V_{RRM} \cup VSD_{SDRRM} \}$$ (**Equation S4**)

References of Protocol S1 Section B

1. Rhee SY, Wood V, Dolinski K, Draghici S (2008) Use and misuse of the gene ontology annotations. Nat Rev Genet 9: 509-515.
2. Barry WT, Nobel AB, Wright FA (2005) Significance analysis of functional categories in gene expression studies: a structured permutation approach. Bioinformatics 21: 1943-1949.
3. Prufer K, Muetzel B, Do HH, Weiss G, Khaitovich P, et al. (2007) FUNC: a package for detecting significant associations between gene sets and ontological annotations. BMC Bioinformatics 8: 41.

Section C. Details of Functional enrichment analysis of miR-204 targets and PPIN using Gene Ontology (Material and Methods, Figures 2B-C, Figure 3C, Table 7 in Text S2).

To provide insights into biological functions and processes potentially regulated by miR-204 in HNSCC, we conducted standard statistical enrichment analyses based on the functional assignments of gene in Gene Ontology (GO) [1] to infer significantly deregulated functions associated with altered miR-204 target expression in the HNSCC according to their presence in the miRNOME and/or the PPINs. Among a total of 1,088 genes predicted as targets of miR-204 in the miRNOME, 34 genes were also identified in the differentially up-regulated gene list (fold change>2) of the HNSCC GSE6631 transcription array. To characterize the functional relationships between 34 predicted targets of miR-204, we established the significant statistical enrichment of gene in to GO dataset with the following parameters of Onto-Express [2]: GO categories (i) "biological process" (GO_BP), and (ii) "molecular function" (GO_MF), cumulative hypergeometric distribution, *P-value* adjusted for multiplicity with FDR, and computational refinement of false positive results computationally inherited in GO (**Protocol S1/Section B**). In addition we also filtered out false positive enrichment results according to the previously described method that we developed (**see Protocol S1/Section B**, **Supporting Figure 8 in Text S1**). To examine the shared biological functions among 34 predicted miR-204 targets, we employed two functional enrichment analyses that prioritized the biological processes and molecular functions of (i) 34 miR-204 targets up regulated in the HNSCC transcription array GSE6631 with the background of 1,088 miR-204 targets from miRNOME, and (ii) 1,088 putative miR-204 targets from the miRNOME with the background of the whole human genome. We focused our evaluation on enriched biological processes and molecular functions (GO terms) that contain at least 3 upregulated miR-204 targets in HNSCC and retained the GO terms enriched in both above-mentioned enrichment analyses (**Table 7 in Text S2**). Additionally, these dually enriched GO terms also had to meet the inclusion criteria of the refinement statistics (see **Protocol S1/Section B**) to filter out false positive results. 30 of the 42 GOs met these criteria (**Figure 2B-C**, and **Table 7 in Text S2**). Similarly, the double GO enrichment method was applied to determine enriched molecular functions and biological processes in the 56 up-regulated HNSCC genes from GSE6631 that are significantly connected (prioritized) in the protein-protein interaction network. For these analyses, two enrichment statistics were calculated: (i) the 56 genes prioritized in PPIN against the 260 PPIN genes up-regulated in HNSCC transcription array GSE6631 as the background, and (ii) the 260 PPIN genes upregulated in HNSCC transcription array GSE6631 against the whole human genome as the background. GO terms significantly enriched in both analyses and with at least 3 genes were considered as valid (**Figure 3C**).

References of Protocol S1 Section C

1. Ashburner M, Ball CA, Blake JA, Botstein D, Butler H, et al. (2000) Gene ontology: tool for the unification of biology. The Gene Ontology Consortium. Nat Genet 25: 25-29.
2. Khatri P, Draghici S, Ostermeier GC, Krawetz SA (2002) Profiling gene expression using onto-express. Genomics 79: 266-270.

<u>Section D</u>. Details of datasets used to construct the protein-protein interaction network (Figure 3, Table 10 in Text S2).

The protein-protein interaction network (**PPIN**) was generated by integrating seven protein interactions and signaling datasets. Protein interactions from each dataset were standardized to a two-column list of pair wise interactions between SwissProt accession IDs, with an additional column providing the source dataset and references to the literature when available. An overview of the seven datasets used in this study is provided in **Table 10 in Text S2**. As a similar database in recent, Jagadish and colleagues have integrated different sources of protein interaction data to construct protein-protein network [1-3].

Protein interaction data was downloaded from BIND [4] open access (now BOND: http://bond.unleashedinformatics.com) on October 10, 2006 along with the BIND database cross reference file. The interaction list was filtered using associated annotation to exclude Homo sapiens interactions inferred from yeast two-hybrid experiments. BIND identifiers in the interaction file were used to map to SwissProt accession IDs using the BIND-supplied database cross-reference file (downloaded October 10, 2006). Homo sapiens protein interactions were downloaded from the BioGRID [5] (http://www.thebiogrid.org/downloads.php, version 2.0.25) on March 14, 2007 and the header describing the columns of the file was removed. The “all data” dataset of the HUGO Gene Nomenclature Committee (HGNC; **Table 9 in Text S2**) [6] was used to translate the proteins identified in the first two columns of the tab-delimited BioGRID file into SwissProt accession numbers. Using the information in the “experimental systems” column, the dataset was filtered to exclude interactions inferred from Yeast two-hybrid system and from “dosage rescue”. Homo sapiens specific data from the Database of Interacting Proteins [7] (DIP, file Hsapi20070107, http://dip.doe-mbi.ucla.edu/dip/Download.cgi) was downloaded on January 7, 2007 and parsed to extract pairs of SwissProt identifiers. Data was downloaded from the Human Proteome Reference Database [8] (HPRD) (http://www.hprd.org/download) on December 6, 2006 and translated to SwissProt accession numbers using a cross mapping from the HGNC from Entrez Gene identifiers in the original data. Data was then filtered to remove interactions derived from yeast two hybrid experiments. This resulted in a two- column list of pair wise interactions between SwissProt accession IDs. The KEGG Pathway Database (release 40.0, October 2006) was downloaded from The Kyoto Encyclopedia of Genes and Genomes (KEGG) [9] website (http://www.genome.jp/kegg/) and integrated to the PPIN. The file containing protein interactions for mammals was downloaded from the MINT website [10] (http://mint.bio.uniroma2.it/mint/download.do) on Dec. 5, 2006. Annotations pertaining to yeast two-hybrid or to co-localization and visualization technologies methods were excluded. Interacting human proteins (NCBI Taxonomy ID 9,606) were retained for the PPIN. The second dataset of protein interaction in Homo sapiens was downloaded from Reactome.org [11] (http://reactome.org/download/index.html) on October 27, 2006. “Reaction” and “direct complex” type of interactions were retained. SwissProt accession

identifier data was extracted from the columns one and four to create a two-column file of pairwise interactions between SwissProt accession IDs.

References of Protocol S1 Section D

1. Tarcea VG, Weymouth T, Ade A, Bookvich A, Gao J, et al. (2009) Michigan molecular interactions r2: from interacting proteins to pathways. Nucleic Acids Res 37: D642-646.
2. Gao J, Ade AS, Tarcea VG, Weymouth TE, Mirel BR, et al. (2009) Integrating and annotating the interactome using the MiMI plugin for cytoscape. Bioinformatics 25: 137-138.
3. Jayapandian M, Chapman A, Tarcea VG, Yu C, Elkiss A, et al. (2007) Michigan Molecular Interactions (MiMI): putting the jigsaw puzzle together. Nucleic Acids Res 35: D566-571.
4. Bader GD, Donaldson I, Wolting C, Ouellette BF, Pawson T, et al. (2001) BIND--The Biomolecular Interaction Network Database. Nucleic Acids Res 29: 242-245.
5. Stark C, Breitkreutz BJ, Reguly T, Boucher L, Breitkreutz A, et al. (2006) BioGRID: a general repository for interaction datasets. Nucleic Acids Res 34: D535-539.
6. Eyre TA, Ducluzeau F, Sneddon TP, Povey S, Bruford EA, et al. (2006) The HUGO Gene Nomenclature Database, 2006 updates. Nucleic Acids Res 34: D319-321.
7. Xenarios I, Rice DW, Salwinski L, Baron MK, Marcotte EM, et al. (2000) DIP: the database of interacting proteins. Nucleic Acids Res 28: 289-291.
8. Peri S, Navarro JD, Amanchy R, Kristiansen TZ, Jonnalagadda CK, et al. (2003) Development of human protein reference database as an initial platform for approaching systems biology in humans. Genome Res 13: 2363-2371.
9. Kanehisa M, Goto S (2000) KEGG: kyoto encyclopedia of genes and genomes. Nucleic Acids Res 28: 27-30.
10. Chatr-aryamontri A, Ceol A, Palazzi LM, Nardelli G, Schneider MV, et al. (2007) MINT: the Molecular INTeraction database. Nucleic Acids Res 35: D572-574.
11. Vastrik I, D'Eustachio P, Schmidt E, Joshi-Tope G, Gopinath G, et al. (2007) Reactome: a knowledge base of biologic pathways and processes. Genome Biol 8: R39.
